# Efficient generation of complete sequences of MDR-encoding plasmids by rapid assembly of MinION barcoding sequencing data

**DOI:** 10.1093/gigascience/gix132

**Published:** 2018-01-09

**Authors:** Ruichao Li, Miaomiao Xie, Ning Dong, Dachuan Lin, Xuemei Yang, Marcus Ho Yin Wong, Edward Wai-Chi Chan, Sheng Chen

**Affiliations:** 1 Shenzhen Key Lab for Food Biological Safety Control, Food Safety and Technology Research Center, Hong Kong PolyU Shen Zhen Research Institute, Shenzhen, P. R. China; 2 The State Key Lab of Chirosciences, Department of Applied Biology and Chemical Technology, The Hong Kong Polytechnic University, Hung Hom, Kowloon, Hong Kong SAR

**Keywords:** multidrug resistance (MDR) plasmids, *de novo* assembly, nanopore sequencing, long reads

## Abstract

**Background:**

Multidrug resistance (MDR)–encoding plasmids are considered major molecular vehicles responsible for transmission of antibiotic resistance genes among bacteria of the same or different species. Delineating the complete sequences of such plasmids could provide valuable insight into the evolution and transmission mechanisms underlying bacterial antibiotic resistance development. However, due to the presence of multiple repeats of mobile elements, complete sequencing of MDR plasmids remains technically complicated, expensive, and time-consuming.

**Results:**

Here, we demonstrate a rapid and efficient approach to obtaining multiple MDR plasmid sequences through the use of the MinION nanopore sequencing platform, which is incorporated in a portable device. By assembling the long sequencing reads generated by a single MinION run according to a rapid barcoding sequencing protocol, we obtained the complete sequences of 20 plasmids harbored by multiple bacterial strains. Importantly, single long reads covering a plasmid end-to-end were recorded, indicating that *de novo* assembly may be unnecessary if the single reads exhibit high accuracy.

**Conclusions:**

This workflow represents a convenient and cost-effective approach for systematic assessment of MDR plasmids responsible for treatment failure of bacterial infections, offering the opportunity to perform detailed molecular epidemiological studies to probe the evolutionary and transmission mechanisms of MDR-encoding elements.

## Introduction

The emergence and increasing prevalence of antimicrobial resistance (AMR) among bacterial pathogens pose increasing public health challenges worldwide by drastically reducing the number of antimicrobials that can be effectively used in treatment of bacterial infections [1, 2]. Identification of the key mechanisms responsible for AMR transmission is crucial to combat the threats imposed by AMR. Plasmids, especially MDR-encoding plasmids, are now considered a major vector that facilitates AMR transmission among bacteria via horizontal transfer [3, 4]. Delineating the full length of plasmids and genetic structures of other MDR mobile elements is vital for understanding how such elements undergo evolutionary changes and horizontal transmission and adapt to new hosts [4]. However, due to the presence of numerous insertion sequences and other repetitive elements in MDR plasmids, it is often difficult and time-consuming to obtain the complete plasmid sequences by next-generation sequencing with short reads and polymerase chain reaction (PCR) mapping by Sanger sequencing. With the development of long read sequencing technology, tracking plasmid diversity by full assembly of plasmids has become possible [5]. To date, single-molecule, real-time sequencing (SMRT) can generate full-sequence plasmids. However, the huge cost and laborious library preparation procedure of this technology renders it inaccessible for most laboratories.

Recently, another long read sequencing technology based on the use of a portable MinION device has become available from Oxford Nanopore Technologies (ONT). Although the accuracy of reads generated by this technique is generally lower than that of short reads, it exhibits a promising capability to generate complete chromosome and plasmid sequences [6, 7]. With the advance of library preparation techniques and data analysis tools, we found that this technology is feasible for MDR plasmid sequencing. Here, we evaluated the feasibility of decoding the complete sequences of multiple MDR plasmids using MinION Nanopore sequencing technology through a run with a reusable flow cell within a short time frame. This workflow shall enable laboratories equipped with only basic molecular biology techniques to perform detailed MDR plasmid analysis.

## Data Description

Raw long sequencing data collected after a MinION run were de-multiplexed by Albacore basecalling software (v1.0.3) to generate fast5 files allocated into 12 samples. The Poretools tool suite was used to extract reads with fasta format and proceded to *de novo* assembly and hybrid assembly with Canu (v1.3) and Unicycler (v0.3). The end result was 20 complete plasmids and 1 near-complete plasmid that were efficiently obtained with the data from a single MinION run. The detailed procedures for data analysis are described in the Methods.

## Results

### MinION workflow overview

**Figure 1:**
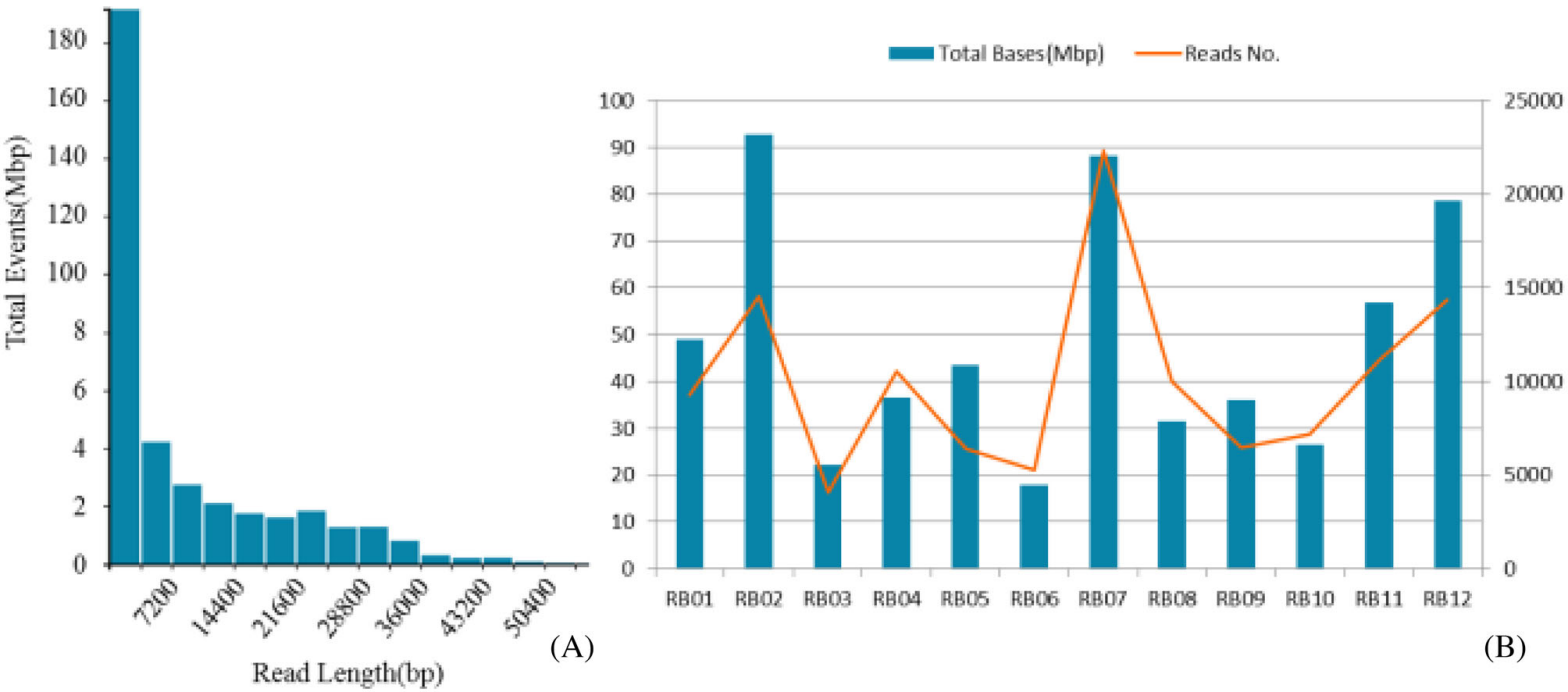
Statistics of an 8-hour MinION nanopore sequencing run using the Rapid Barcoding Sequencing Kit. **(A)**, Distribution of read length and data volume generated by the MinION run in 8 hours. **(B)**, Total base length and read number of the 12 samples after de-multiplexing.

Twelve MDR plasmids harboring samples were prepared according to the MinION library construction protocols, followed by library sequencing. After 8 hours of sequencing run, a total of 287 725 reads ranging from dozens to tens of thousands of bases in length were obtained, covering a total of 493 Mbp (Fig. 1A). It was estimated that the data should be enough for *de novo* assembly; hence the run was stopped manually to save active nanopores for future use. The raw data were subjected to several stages of processing, including basecalling, de-multiplexing, fasta sequence extraction, and *de novo* assembly, as stated in the Methods section. Upon de-multiplexing, a total of 121 584 reads were allocated into the 12 samples, which ranged from 5273 to 22 319 in read number and 18 to 93 Mbp in total length (Fig. 1B). The reads that were unsuccessfully basecalled and unclassified reads generated during the de-multiplexing process were excluded from the assembly analysis. By optimizing the parameters of the *de novo* assembly tool, we obtained the complete sequences of the MDR plasmids recovered from 11 samples, except RB08, which was severely contaminated by chromosomal DNA.

### Evaluation of plasmid assembly efficiency

**Figure 2:**
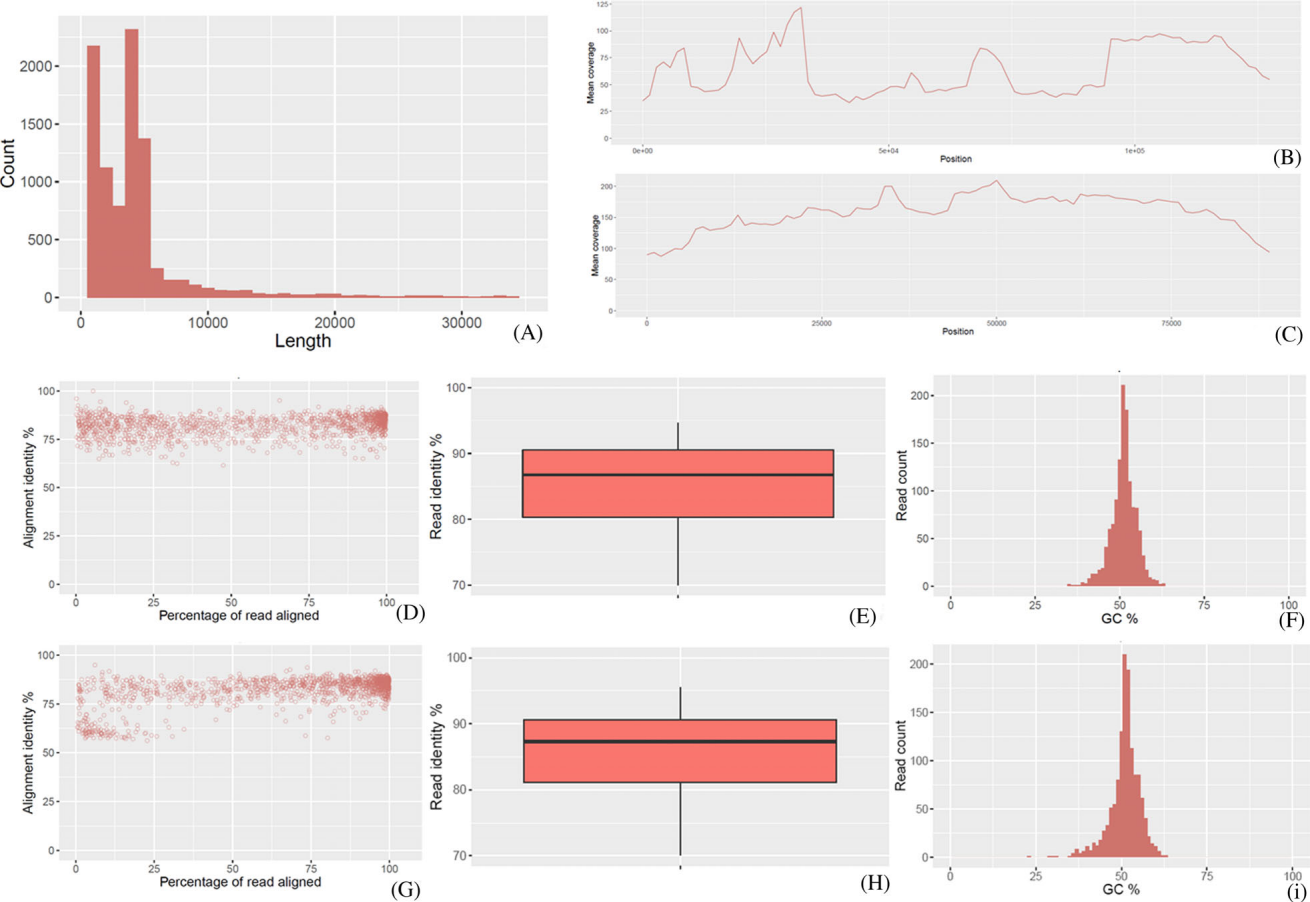
Evaluation of MinION nanopore sequencing long reads quality with nanonet. **(A)**, Read counts along with reads length for sample RB01. All the raw reads could be retrieved from the Supplementary Data. **(B)**, Nanopore read coverage with RB01-LZ135-CTX-128 976 as reference. **(C)**, Nanopore read coverage with RB01-LZ135-NDM-90 845 as reference. (**D–F**), Alignment identity and **GC** distribution for reads aligned with RB01-LZ135-CTX-128 976. (**G–I**), Alignment identity and GC distribution for reads aligned with RB01-LZ135-NDM-90 845.

Apart from sample RB08, *de novo* assembly was successfully performed on 11 MDR plasmids harboring samples by Canu. High-quality assembled sequences were obtained using Unicycler by combining with short read data. One to 5 plasmids, which ranged from 46 to 238 Kb in length, were found in each sample, with a total of 20 complete and 1 near-complete plasmids being obtained from 11 samples (Table 1). To evaluate the accuracy of *de novo* assembly of rapid 1D sequencing data generated by the MinION platform, the RB01 sample was selected for comparison between pair-end Illumina sequencing data and nanopore sequencing data. The sequences of 2 plasmids, RB01-LZ135-CTX-128 976 and RB01-LZ135-NDM-90 845, were selected for evaluation of the nanopore reads’ quality (Fig. 2). Without size selection during library preparation, the read lengths ranged from 18 to 97 206 bp, and the N50 was 6473 bp. Based on the alignment of reads to 2 reference plasmids, the MinION nanopore long reads’ accuracy was about 87%.

**Table 1:** Basic data of 12 MDR plasmids harboring samples used in the single multiplexed MinION run

Samples	Marker genes	Species	Plasmid profile, kb^a^	7.5 μL, ng^b^	Volume, μL^c^	Quantity, ng^d^
RB01	*bla*_NDM-5_	*Escherichia coli*	150,100	750	0.8	60
RB02	*bla*_NDM-5_	*Escherichia coli*	160, 135, 100, 60, 40	2010	0.4	160.8
RB03	*bla*_NDM-1_	*Escherichia coli*	330, 60	259.5	1.1	20.76
RB04	*bla*_NDM-1_	*Escherichia coli*	110, 130, 230	937.5	0.7	75
RB05	*bla*_CTX-M-15_	*Escherichia coli*	150	484.5	0.8	38.76
RB06	*bla*_CTX-M-15_	*Escherichia coli*	250	270	1	21.6
RB07	*bla*_CTX-M-15_	*Vibrio parahaemolyticus*	120	654	0.8	52.32
RB08	*bla*_CTX-M-3_, *bla*_TEM-1_	*Salmonella* typhimurium	340	885	0.8	70.8
RB09	*bla*_KPC-2_	*Escherichia coli*	70	639	0.8	51.12
RB10	*bla*_KPC-2_	*Escherichia coli*	100, 130	346.5	1.1	27.72
RB11	*bla*_KPC-2_	*Klebsiella pneumoniae*	240	1125	0.8	90
RB12	*bla*_KPC-2_	*Escherichia coli*	120, 100	495	0.9	39.6

^a^Plasmid profile was determined by S1 nuclease PFGE; the sizes of the plasmids were roughly estimated based on S1-PFGE.

^b^The input quantities of plasmid DNA in 7.5 μL during library preparation.

^c^The volume of each sample in the 10-μL pooled library.

^d^The actual quantity of DNA of each sample used in MinION sequencing.

**Figure 3:**
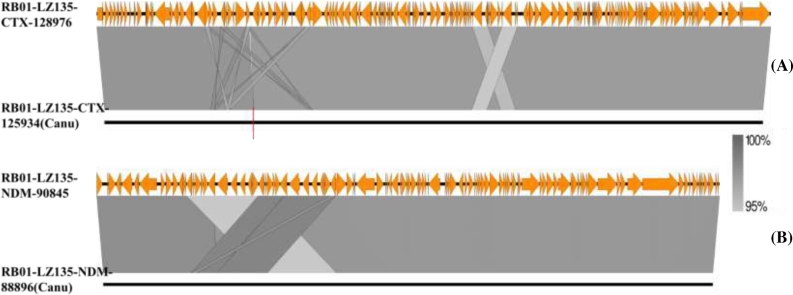
Linear alignment of reference plasmids and corresponding plasmids assembled by Canu based only on MinION nanopore long reads of sample RB01. **(A)**, Alignment of RB01-LZ135-CTX-128 973 and RB01-LZ135-CTX-125 934 (Canu). A large deletion that existed in the plasmid RB01-LZ135-CTX-125 934 is marked by a red vertical line. **(B)**, Alignment of RB01-LZ135-NDM-90 845 and RB01-LZ135-NDM-88 896 (Canu). The crossed alignment region indicates that the *bla*_NDM-5_region was duplicated. The 2 plasmid sequences assembled by Canu could be retrieved from the Supplementary Data. The 2 reference plasmids were deposited in the NCBI database.

Complete plasmid sequences obtained from *de novo* assembly by Canu based on long reads were compared with the reference plasmids (assembled by Unicycler) by BLASTN. The overall identity of the completed plasmids by Canu was 97% identical to the reference plasmids; the difference was mainly due to fabricated deletions in plasmids assembled by Canu, resulting in an overall sequence 3043 bp and 1949 bp shorter than RB01-LZ135-CTX-128 976 and RB01-LZ135-NDM-90 845 respectively. No major structural variations were observed between the 2 different *de novo* assembly methods (Fig. 3), indicating that nanopore long reads can be used to accurately resolve the mosaic structures frequently found in plasmids.

Using only Nanopore data to complete plasmids of interest was recommended when no short read data were available. The hybrid assembly used Unicycler, the accurate way to obtain complete genome sequences. For sequences that cannot be resolved by Unicycler, Canu can be an option. Detailed comparison results using hybrid assembly by Unicycler and Nanopore data–based assembly by Canu are described in Table S1. It is suggested that the advantages of assembly using Canu include high efficiency, real-time monitoring, and cost-effectiveness, while the disadvantage is its lower accuracy when compared with the hybrid assembly approach using Unicycler. With the development of ONT technologies that significantly improve the accuracy of single reads, assembly using Canu is likely the best choice going forward.

### Characterization of MDR plasmids

**Figure 4:**
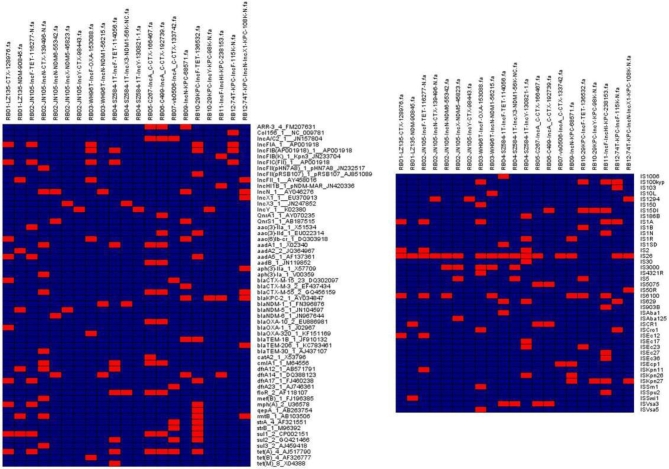
Distribution of resistance genes, replicon genes, and insertion sequences among 21 plasmids. Red boxes indicate the presence of corresponding genes, and blue boxes indicate absence of the corresponding genes. The 21 plasmid sequences can be retrieved from the Supplementary Data. It should be noted that 1 plasmid, RB04-SZ584-1T-IncX3-NDM1-56K-NC, was not fully completed.

The number of resistance genes detectable among the 20 complete and 1 near-complete plasmids sequenced in this study ranged from 0 to 12, insertion sequences from 1 to 10, and replicon genes from 1 to 4 (Table 2, Fig. 4). This implied that the plasmids tested in this study had complex structures, the complete sequences of which were usually difficult to obtain by short read sequencing technology due to the presence of numerous repetitive sequences.

**Table 2:** Overview of structures and genetic characteristics of 21 MDR plasmids recovered from 11 samples

Plasmids^a^	Size, bp	Structural status	No. of resistance genes	No. of insertion sequences	No. of replicon genes
RB01-LZ135-CTX-128976	128976	Circular	8	5	2
RB01-LZ135-NDM-90845	90845	Circular	5	2	1
RB02-JN105-IncF-TET-116277-N	116277	Circular	6	6	2
RB02-JN105-IncN-CTX-139496-N	142307	Circular	9	2	2
RB02-JN105-IncN-NDM6-55342	55342	Circular	3	3	1
RB02-JN105-IncX-NDM5-45823	45823	Circular	1	4	1
RB02-JN105-IncY-CTX-98443	98443	Circular	0	1	1
RB03-WH96T-IncF-OXA-153088	153088	Circular	3	9	4
RB03-WH96T-IncN-NDM1-56215	56215	Circular	2	4	1
RB04-SZ584-1T-IncF-TET-114056	114065	Circular	7	6	2
RB04-SZ584-1T-IncX3-NDM1-56K-NC^b^	55919	Linear	2	4	1
RB04-SZ584-1T-IncY-130821	130821	Circular	0	9	1
RB05-C267-IncA/C-CTX-166467	166467	Circular	10	3	1
RB06-C499-IncA/C-CTX-192739	192739	Circular	11	3	1
RB07-vb0506-IncA/C-CTX-133742	133742	Circular	6	2	1
RB09-IncN-KPC-68571	68571	Circular	7	6	1
RB10-29KPC-IncF-TET-136532	136532	Circular	12	6	3
RB10-29KPC-IncY-KPC-98K-N	95908	Circular	1	2	1
RB11-IncF-IncHI-KPC-238153	238153	Circular	2	10	2
RB12-74T-KPC-IncF-115K-N	115689	Circular	0	6	4
RB12-74T-KPC-IncN-IncX1-KPC-108K-N	107969	Circular	5	4	3

^a^Plasmid names ending with the letter N indicated that the plasmids could be assembled by Canu, based on MinION nanopore reads, but could not be assembled using the hybrid assembly strategy with Unicycler. Plasmid names ending with NC indicated incomplete assembly due to low coverage of reads resulting from the low copy number of large plasmids.

^b^Although this plasmid was not fully completed, it was still used to do further analysis together with other plasmids.

To demonstrate the ability of nanopore long reads to resolve the complex structures of MDR plasmids, sample RB01 was investigated in detail. Upon *de novo* assembly, 2 complete plasmids were obtained and designated as RB01-LZ135-CTX-128 976 and RB01-LZ135-NDM-90 845, respectively. This sample originated from a clinical carbapenem-resistant *E. coli* strain harboring the *bla*_CTX-M-15_ and*bla*_NDM-5_ genes, which was reported previously [8].

**Figure 5:**
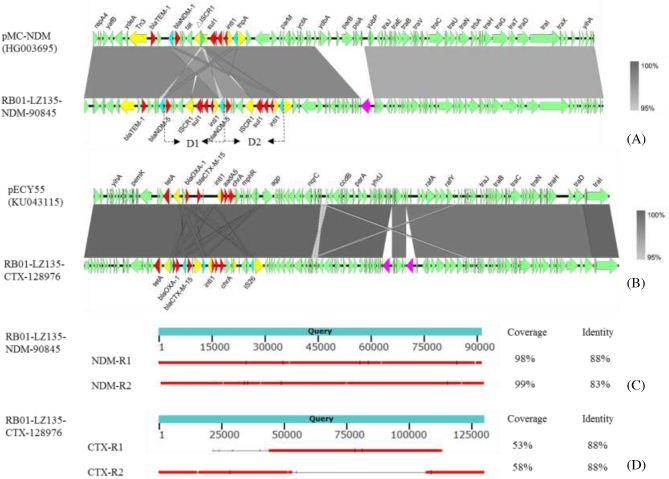
Alignment of plasmids in RB01 with similar structures in the NCBI database and MinION nanopore long read alignment with complete plasmids. **(A)**, Alignment between pMC-NDM and RB01-LZ135-NDM-90 845. The resistance genes are highlighted in red, transposase genes in yellow, IS*26* in cyan, group II intron gene in pink, and other CDS in light green. The sequence contained a large duplication region (ca.10kbp) designated as D1 and D2, each harboring a class 1 integron and a*bla*_NDM-1_ cluster. **(B)**, Alignment between pECY55 and RB01-LZ135-CTX-128 976. The CDS were labeled according to the labeling scheme in Figure 5A. The same group II intron genes were inserted and duplicated in RB01-LZ135-CTX-128 976 compared with pECY55. **(C)**, BLASTN of 2 MinION long reads against RB01-LZ135-NDM-90 845. The results indicated that the whole plasmid could be sequenced end to end. **(D)**, BLASTN of 2 MinION long reads against RB01-LZ135-CTX-128 976. The results indicated that 2 MinION long reads could cover the entire plasmid. The 4 long reads could be retrieved from the Supplementary Data.

In the IncFII type plasmid RB01-LZ135-NDM-90 845, which was 90 845 bp in length, there was an MDR mosaic region composed of a Tn*3* transposon containing the *bla*_TEM-1_ and *rmtB* genes, and IS*26*-IS*Aba125*-*bla*_NDM-5_-*ble*_MBL_-*traF*-*tat*-IS*CR1*-*sul1*-*qacEdelta1*-*aadA2*-*dfrA12*-*intI1*-IS*26*. Intriguingly, the latter fragment was duplicated in a tandem repeat format (Fig. 5A). Online BLASTN of this *bla*_NDM-5_-bearing plasmid in the NCBI database showed that it was highly similar to the plasmid pMC-NDM, which was recovered from a metallo-beta-lactamase-producing *E. coli* strain in Poland (GenBank no. HG003695), with 99% identity at 97% coverage. The 2 major differences include existence of tandem repeats and a region replacement (Fig. 5A).The *bla*_CTX-M-15_-bearing plasmid RB01-LZ135-CTX-128 976 was 128 976 bp in length and had a conserved structure similar to that found in plasmid pECY55, which was harbored by a previously reported *E. coli* strain (GenBank no. KU043115), with 99% identity at 97% coverage. The MDR region harboring *tetA, aac(6’)-Ib-cr, bla*_OXA-1_, *bla*_CTX-M-15_, *dfrA17, aadA5, sul1, chrA*, and *mph*(A) was shared by these 2 plasmids, and 2 group II introns were found inserted in the backbone compared with pECY55 (Fig. 5B). Detailed analysis of the longest reads after BWA MEM alignment showed that 2 long reads spanned the plasmid RB01-LZ135-NDM-90 845 end to end, and another 2 long reads could be aligned to generate plasmid RB01-LZ135-CTX-128 976 (Fig. 5C and D). This is the first case in which the whole plasmid sequence could be generated by only 1 single read.

## Discussion

The advent of next-generation sequencing technologies revolutionizes the study mode in genomic research [9]. Specifically, it has tremendously facilitated molecular epidemiology studies and research on the diversity and evolution of MDR-encoding elements from both clinical and basic research perspectives [4, 10]. Although it is feasible to assess the distribution of resistance genes among single bacterial or metagenomic samples with traditional short read data, constructing the entire plasmid and chromosome maps that depict the specific location of resistance genes is of vital importance in investigating the evolutionary features of such genes and tracking the evolution and transmission routes of MDR plasmids [4, 11]. The availability of long read sequencing technologies such as SMRT and MinION nanopore sequencing has shed light on the development of efficient approaches to assemble complete genomes with numerous repetitive elements [12, 13]. Owing to the high cost and complex library preparation of SMRT technology, it cannot be commonly utilized in clinical settings and basic molecular laboratories, although this technology has been commercially available for more than 5 years. On the contrary, the recently available portable MinION nanopore sequencing technology offers the opportunity to be used anywhere as long as a laptop computer is available. In this study, we evaluated the possibility of MinION nanopore sequencing technology to resolve the mosaic MDR plasmids with the latest R9.4 chemistry.

**Figure 6:**
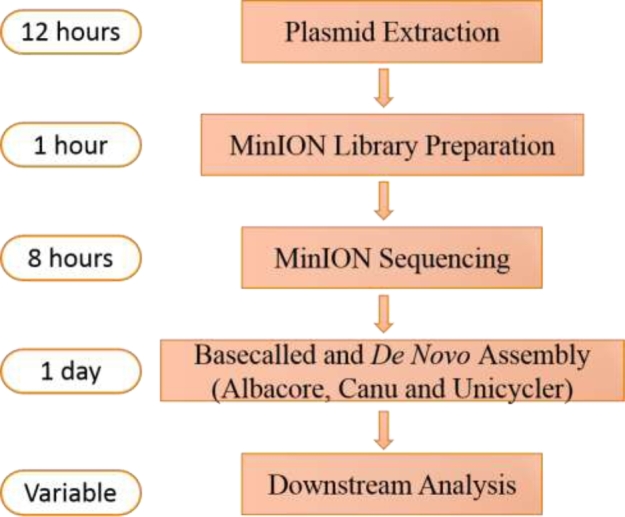
Workflow and time span overview of the MinION nanopore sequencing and assembly process. This workflow was based on the rapid barcoding sequencing kit, which could pool 12 samples in a single run. The time for basecalling and *de novo* assembly depended on the computational performance of the computer utilized, and Illumina short reads were needed if Unicycler was used to obtain high-quality assembled plasmids.

With the rapid barcoding sequencing kit, the complete sequences of 20 complete (and 1 near-complete) plasmids harbored by 11 samples could be successfully generated within a few days (Fig. 6). Although *de novo* assembly of only nanopore long reads by Canu exhibited a relatively low quality of only 97% identity to the reference sequences, the assembled plasmids were found to possess high-quality structural skeletons with correct arrangements of various mobile elements. With Illumina short read data, accurate complete sequences of plasmids could be obtained by Unicycler, which involved 3 steps: contig construction with short reads, scaffolding of contigs with long reads, and polishing with short reads [14]. Importantly, analysis of the 2 MDR plasmids in sample RB01 indicated that single long reads could cover a complete plasmid; this finding inferred that the entire plasmid can be sequenced without interruption. In this case, *de novo* assembly was not necessary since several long reads may cover the whole plasmid. The first antibiotic resistance island that was resolved by MinION nanopore sequencing was reported in 2015 [7]. To the best of our knowledge, this is the first report of complete MDR plasmid sequencing without the need to assemble sheared fragments. It should be noted that although only a few long reads were found to cover the entire plasmid, they were sufficient to cover all the repetitive sequences in the MDR plasmids. With further improvement in MinION sequencing, a plasmid being sequenced end-to-end as a single molecule will become possible in the near future.

Another advantage of MinION sequencing is that it allows halting of an ongoing sequencing run when sufficient data have been achieved, saving time and most importantly the flow cell, which accounts for a significant portion of the cost of MinION sequencing. As a result, the flow cell can be reused several times until most of nanopores have lost activity. In this work, we finished the run in 8 hours, during which the MinION generated sufficient data for assembling the complete plasmid sequences. Furthermore, the same flow cell was reused in another run, and the data generated were of similar quality to that of the first run. The standard MinKNOW protocol involves running the flow cells for 48 hours. If 1 flow cell can accommodate 3 runs, each lasting for 8, 10, and 12 hours, respectively, it indicates that 36 MDR plasmids harboring samples can be sequenced in 1 flow cell using the rapid barcode kit, leading to significant reduction in the cost of producing complete plasmid sequences. Furthermore, the real-time hybrid genome assembly approach was reported with the npScarf tool, which can overcome oversequencing issues and shorten the analysis timeline [13]. This real-time analysis workflow has the potential to be combined with the plasmid assembly workflow described in this study.

As an extra chromosomal element, plasmids play a dominant role in the dissemination of antibiotic resistance genes, virulence genes, and other functional genes [15, 16]. Obtaining complete plasmid sequences in a wide range of clinical isolates collected over a prolonged period enables in-depth studies of plasmid evolution and adaptation, the underlying mechanisms of transmission of resistance genes, as well as tracking major antibiotic-resistant pathogenic bacterial strains [5, 16, 17]. The workflow presented in this work offers for the first time the opportunity to perform these studies in a rapid, cost-effective, and user-friendly manner.

## Methods

### Bacterial MDR plasmids extraction

To evaluate the efficiency of MDR plasmid sequencing by MinION platform, we selected 12 MDR plasmid-bearing strains including *E. coli, S. typhimurium, V. parahaemolyticus*, and *K. pneumoniae* for plasmid extraction (Table 1). Overnight cultures (100 mL) were harvested and subjected to plasmid extraction using the QIAGEN Plasmid Midi Kit. The extracted plasmids were dissolved in ultrapure distilled water, and concentrations were measured by Qubit 3.0 Fluorometer with a dsDNA BR Assay Kit. The plasmids were stored in –20°C until library preparation.

### MinION library preparation and sequencing

Library preparation was performed using the Rapid Barcoding Sequencing Kit (SQK-RBK001) according to the standard protocol provided by the manufacturer (Oxford Nanopore). Briefly, 7.5-μL plasmid templates were combined with a 2.5-μL Fragmentation Mix Barcode (1 barcode for each sample). The mixtures were incubated at 30°C for 1 minute and at 75°C for 1 minute. The barcoded libraries were pooled together with designated ratios in 10 μL (Table 1); 1 μL of RAD (Rapid 1D Adapter) was added to the pooled library and mixed gently; 0.2 μL of Blunt/TA Ligase Master Mix was added and incubated for 5 minutes at room temperature. The constructed library was loaded into the Flow Cell R9.4 (FLO-MIN106) on a MinION device and run with the SQK-RBK001_plus_Basecaller script of MinKNOW1.5.12 software. The run was stopped after 8 hours, and the flow cell was washed by a Wash Kit (EXP-WSH002) and stored in 4°C for later use.

### Illumina sequencing

To obtain high-quality short read data, paired-end (2 × 150 bp) libraries were prepared by the focused acoustic shearing method with the NEBNext Ultra DNA Library Prep Kit and the Multiplex Oligos Kit for Illumina (NEB). The libraries were quantified by employing quantitative PCR with P5-P7 primers, and they were pooled together and sequenced on the NextSeq 500 platform according to the manufacturer's protocol (Illumina).

### Basecalling, de-mutiplexing, assembly of complete plasmid sequences, and data analysis

Although a local basecaller script was used during the run, there was still a small amount of reads that were not basecalled due to the generation of raw data in a rapid mode. Albacore basecalling software (v1.0.3) was used to generate fast5 files harboring the 1D DNA sequence from fast5 files with only raw data in the tmp folder. Also, the read_fast5_basecaller.py script in Albacore was used to de-multiplex the 12 samples from basecalled fast5 files (except the files in fail folder) based on the 12 barcodes in SQK-RBK001. The Poretools toolkit was utilized to extract all the DNA sequences from fast5 to fasta format among the 12 samples, respectively (Poretools, RRID:SCR_015879) [18]. The Canu assembly tool (v1.3; Canu, RRID:SCR_015880) [14] was used to perform *de novo* assembly of complete plasmid sequences based on nanopore 1D long reads in 3 consecutive stages including correction, trimming, and assembly [14]. Due to the possibility of the contamination of bacterial chromosomal DNA in the plasmid samples and the large variation of the size of the plasmids, the parameter of genomeSize was set at 0.5, 1, 2, and 4 m, respectively, to optimize the assembly results to obtain circular plasmid sequences of interest. The sizes and the numbers of plasmids determined by S1–pulsed-field gel electrophoresis (PFGE) were used to confirm the assembled results. High-quality complete plasmids were constructed by hybrid *de novo* assembly of Illumina short reads and nanopore long reads data using the Unicycler v0.3 tool [19]. NanoOK was adopted to evaluate the quality of nanopore long reads [20]. BWA MEM was used to align long reads against reference plasmids (BWA, RRID:SCR_010910) [21].

To assess the distribution of resistance genes, mobile elements, and replicon genes, the corresponding databases were downloaded [21–23] and BLASTN was performed among the finished plasmids (BLASTN, RRID:SCR_001598). The result was visualized by the tool Genesis [24]. Easyfig was utilized to compare the detailed structures of the MDR plasmids (Easyfig, RRID:SCR_013169) [25].

## Availability of supporting data

Raw MinION and Illumina sequencing data are available in the NCBI database via the BioProject number PRJNA398365. The 20 complete and 1 near-complete plasmid sequences of the 12 samples were included as Supplementary Data in the *GigaScience* database, *Giga*DB. The 2 plasmids in sample RB01 were deposited in NCBI with the accession numbers MF353155 and MF353156. The 2 plasmids assembled by only MinION nanopore long reads in sample RB01 are also included as Supplementary Data for reference in *Giga*DB.

## Abbreviations

AMR: antimicrobial resistance; BLAST: Basic Local Alignment Search Tool; MDR: multidrug resistance; NCBI: National Center for Biotechnology Information; ONT: Oxford Nanopore Technologies; PCR: polymerase chain reaction; PFGE: pulsed-field gel electrophoresis; SMRT: single-molecule, real-time sequencing.

## Additional files

Supplementary Table S1. Comparison between plasmid sequences of first 4 samples assembled by Unicycler using both Illumina and Nanopore data and by Canu using only Nanopore data.

Supplementary data 1-RB01 plasmids by Canu.fa

Supplementary data 2-twenty one plasmids.fa

Supplementary data 3-four long reads.fa

## Author contributions

R.L. conceived and initiated the study. M.X., N.D., and D.L. performed bacterial isolation and plasmids extraction. R.L., X.Y., and M.H.W. performed MinION and Illumina sequencing and data analysis. R.L. wrote the first draft of the manuscript. E.W.C. revised the manuscript. S.C. supervised the whole project and edited the manuscript.

## Competing interests

The authors declare no competing financial interests.

## Supplementary Material

GIGA-D-17-00150_Original_Submission.pdf

GIGA-D-17-00150_Revision_1.pdf

GIGA-D-17-00150_Revision_2.pdf

Response_to_Reviewer_Comments_Original_Submission.pdf

Response_to_Reviewer_Comments_Revision_1.pdf

Reviewer_1_Report_(Original_Submission) -- Son Hoang Nguyen 21 Jul 2017 Reviewed

Reviewer_1_Report_(Revision_1) -- Son Hoang Nguyen 10 Oct 2017 Reviewed

Reviewer_2_Report_(Original_Submission) -- Timothy Dallman 08 Aug 2017 Reviewed

Supplemental material
