# Erratum to: Efficient generation of complete sequences of MDR-encoding plasmids by rapid assembly of MinION barcoding sequencing data

**DOI:** 10.1093/gigascience/giz031

**Published:** 2019-03-14

**Authors:** Ruichao Li, Miaomiao Xie, Ning Dong, Dachuan Lin, Xuemei Yang, Marcus Ho Yin Wong, Edward Wai-Chi Chan, Sheng Chen

**Affiliations:** 1 Shenzhen Key Lab for Food Biological Safety Control, Food Safety and Technology Research Center, Hong Kong PolyU Shen Zhen Research Institute, Shenzhen, P. R. China; 2 The State Key Lab of Chirosciences, Department of Applied Biology and Chemical Technology, The Hong Kong Polytechnic University, Hung Hom, Kowloon, Hong Kong SAR

## Correction

In the final publication of “P Efficient generation of complete sequences of MDR-encoding plasmids by rapid assembly of MinION barcoding sequencing data,” by Ruichao Li et al. [1], the data citation linking the article to its supporting data in the *GigaScience* GigaDB repository was not included [2]. Hosted here were the twenty plasmid sequences of the twelve samples, the two plasmids assembled by only MinION nanopore long reads in sample RB01, alongside custom scripts. The data citation has now been included and the corrected article is available in *GigaScience*, volume 7, issue 3.

The Publisher regrets this error.
