# Supplementary material for: Efficient generation of complete sequences of MDR-encoding plasmids by rapid assembly of MinION barcoding sequencing data
Source: Gigascience. 2018 Jan 9;7(3):gix132. doi: 10.1093/gigascience/gix132 (PMC5848804; doi:10.1093/gigascience/gix132)
Supplement: GIGA-D-17-00150_Original_Submission.pdf [file gix132_giga-d-17-00150_original_submission.pdf]

GigaScience

Efficient generation of complete sequences of MDR-encoding plasmids by rapid assembly of MinION barcoding sequencing data

--Manuscript Draft--

**Manuscript Number:**	GIGA-D-17-00150
**Full Title:**	Efficient generation of complete sequences of MDR-encoding plasmids by rapid assembly of MinION barcoding sequencing data
**Article Type:**	Research
**Funding Information:**	973 (2013CB127200) — Dr Sheng CHEN<br>CRF (C7038-15G) — Dr Sheng CHEN
**Abstract:**	Background：Multidrug resistance (MDR)-encoding plasmids are considered major molecular vehicles responsible for transmission of antibiotic resistance genes among bacteria of the same or different species. Delineating the complete sequence of such plasmids could provide valuable insight into the evolution and transmission mechanisms underlying bacterial antibiotic resistance development. However, due to the presence of multiple repeats of IS elements, complete sequencing of MDR plasmids remains technically complicated, expensive and time consuming.<br><br>Results：Here, we demonstrate a rapid and efficient approach to obtain multiple MDR plasmid sequences through the use of the MinION nanopore sequencing platform, which is incorporated in a portable MinION device. By assembling the long sequencing reads generated by a single MinION run according to a rapid barcoding sequencing protocol, we obtained the complete sequence of 21 plasmids harbored by bacterial strains recovered from multiple samples. Importantly, single long reads covering a plasmid end-to-end were recorded, indicating that de novo assembly may be unnecessary if the single reads exhibit high accuracy.<br><br>Conclusions：This workflow represents a novel, convenient and cost effective approach for systematic assessment of the full range of MDR plasmids responsible for causing failure in antimicrobial treatment of bacterial infections, offering for the first time the opportunity to perform detailed molecular epidemiological studies to probe the evolutionary and transmission routes / mechanisms of MDR-encoding elements.
**Corresponding Author:**	Sheng CHEN<br>Hong Kong Polytechnic University<br>Hung Hom, HONG KONG
**Corresponding Author Secondary Information:**	
**Corresponding Author's Institution:**	Hong Kong Polytechnic University
**Corresponding Author's Secondary Institution:**	
**First Author:**	Ruichao Li
**First Author Secondary Information:**	
**Order of Authors:**	Ruichao Li<br>Miaomiao Xie<br>Ning Dong<br>Dachuan Lin<br>Xuemei Yang<br>Marcus Wong<br>Edward Chan

Powered by Editorial Manager® and ProduXion Manager® from Aries Systems Corporation

	Sheng CHEN
**Order of Authors Secondary Information:**	
**Opposed Reviewers:**	
**Additional Information:**	
**Question**	**Response**
Are you submitting this manuscript to a special series or article collection?	No
**Experimental design and statistics**<br><br>Full details of the experimental design and statistical methods used should be given in the Methods section, as detailed in our Minimum Standards Reporting Checklist. Information essential to interpreting the data presented should be made available in the figure legends.<br><br>Have you included all the information requested in your manuscript?	Yes
**Resources**<br><br>A description of all resources used, including antibodies, cell lines, animals and software tools, with enough information to allow them to be uniquely identified, should be included in the Methods section. Authors are strongly encouraged to cite Research Resource Identifiers (RRIDs) for antibodies, model organisms and tools, where possible.<br><br>Have you included the information requested as detailed in our Minimum Standards Reporting Checklist?	Yes
**Availability of data and materials**<br><br>All datasets and code on which the conclusions of the paper rely must be either included in your submission or deposited in publicly available repositories (where available and ethically appropriate), referencing such data using a unique identifier in the references and in the "Availability of Data and Materials" section of your manuscript.<br><br>Have you have met the above requirement as detailed in our Minimum Standards Reporting Checklist?	Yes

Powered by Editorial Manager® and ProduXion Manager® from Aries Systems Corporation

Powered by Editorial Manager® and ProduXion Manager® from Aries Systems Corporation

Manuscript

Click here to download Manuscript MinION barcoding sequencing data Giga.docx

Efficient generation of complete sequences of MDR-encoding plasmids by rapid assembly of MinION barcoding sequencing data

Ruichao Li[1,2], Miaomiao Xie[1], Ning Dong[1], Dachuan Lin[1,2], Xuemei Yang[1], Marcus Ho Yin Wong[1], Edward Wai-Chi Chan[2], Sheng Chen[1,2]*

[1] Shenzhen Key Lab for Food Biological Safety Control, Food Safety and Technology Research Center, Hong Kong PolyU Shen Zhen Research Institute, Shenzhen, P. R. China;

[2]The State Key Lab of Chirosciences, Department of Applied Biology and Chemical Technology, The Hong Kong Polytechnic University, Hung Hom, Kowloon, Hong Kong SAR;

Running title: Rapid assembly of MDR plasmids by MinION sequencing data

Keywords: Multi-drug resistance (MDR) plasmids, de novo assembly, nanopore sequencing, long reads

Abstract

Background: Multidrug resistance (MDR)-encoding plasmids are considered major molecular vehicles responsible for transmission of antibiotic resistance genes among bacteria of the same or different species. Delineating the complete sequence of such plasmids could provide valuable insight into the evolution and transmission mechanisms underlying bacterial antibiotic resistance development. However, due to the presence of multiple repeats of IS elements, complete sequencing of MDR plasmids remains technically complicated, expensive and time consuming.

Results: Here, we demonstrate a rapid and efficient approach to obtain multiple MDR plasmid sequences through the use of the MinION nanopore sequencing platform, which is incorporated in a portable MinION device. By assembling the long sequencing reads generated by a single MinION run according to a rapid barcoding sequencing protocol, we obtained the complete sequence of 21 plasmids harbored by bacterial strains recovered from multiple samples. Importantly, single long reads covering a plasmid end-to-end were recorded, indicating that de novo assembly may be unnecessary if the single reads exhibit high accuracy.

Conclusions: This workflow represents a novel, convenient and cost effective approach for systematic assessment of the full range of MDR plasmids responsible for causing failure in antimicrobial treatment of bacterial infections, offering for the first time the opportunity to perform detailed molecular epidemiological studies to probe the evolutionary and transmission routes / mechanisms of MDR-encoding elements.

Introduction

The emergence and increasing prevalence of antimicrobial resistance (AMR) among bacterial pathogens pose increasing public health challenges worldwide by drastically reducing the number of antimicrobials that can be effectively used in treatment of bacterial infections[1,2]. Identification of key AMR drivers is crucial to combat the threats imposed by AMR. Plasmids, especially the multidrug resistance-encoding (MDR) plasmids, are now considered a major vector that facilitates AMR transmission among bacteria via horizontal transfer[3,4]. Delineating the full length of plasmids and genetic structure of other AMR-encoding mobile elements are vital for understanding how such elements undergo evolutionary changes and horizontal transmission, and adapt to new host[4]. However, due to the presence of numerous insertion sequences and other repetitive elements in MDR plasmids, it is often difficult and time-consuming to obtain the complete plasmid sequences by WGS with the short reads generated by Sanger sequencing. With the advent of long read sequencing technology, tracking plasmid diversity by full assembly of plasmids has become possible[5]. To date, single-molecule, real-time (SMRT) sequencing can generate full-sequence plasmids; however, the huge cost and laborious library preparation procedure of this technology renders it inaccessible for most laboratories. Recently, another long read nanopore sequencing technology based on the use of a portable MinION device has been available from Oxford Nanopore Technologies (ONT). Although the accuracy of reads generated by this technique is generally lower than that of short reads, it exhibits promising capability to generate complete chromosome and plasmid sequences[6,7]. With the advent of library preparation techniques and data analysis tools, we found that this technology is amenable for MDR plasmid sequencing. Here, we evaluated the feasibility of decoding the complete sequences of multiple MDR plasmids by the MinION nanopore

sequencing technology, through a run with a reusable flow cell within a short time frame. This workflow shall enable even laboratories equipped with only basic molecular biology techniques to perform detailed MDR plasmids analysis.

Data description

Raw long sequencing data collected after MinION run was demultiplexed by Albacore basecalling software (v1.0.3) to generate fast5 files allocated into twelve samples. Poretools tool was used to extract reads with fasta format and preceded to de novo assembly and hybrid assembly with Canu (v1.3) and Unicycler (v0.3). At last, twenty one plasmids were obtained with single MinION run data efficiently. The detailed procedures for data analysis were described in Methods.

Results

MinION workflow overview

Twelve MDR plasmid samples were prepared according to the MinION library construction protocols, followed by library sequencing. After eight hours of sequencing run, a total of 287,725 reads ranging from dozens to tens of thousands of bases in length were obtained, covering 493Mbp (**Figure 1a**). It was estimated that the data should be enough for de novo assembly, hence the run was stopped manually to save active nanopores for future use. The raw data was subjected to several stages of processing including base-calling, de-multiplexing, fasta sequence extraction and de novo assembly as stated in the Methods section. Upon de-multiplexing, a total of 121,584 reads were allocated into the twelve samples, which ranged from 5,273 to 22,319 in reads number and 18 to 93 Mbp in length (**Figure 1b**). The reads that were excluded from the assembly analysis were unsuccessfully base-called reads and unclassified reads obtained during the de-multiplexing process. By optimizing the parameters of de novo assembly tool, we obtained the complete sequences of all the MDR plasmids recovered from eleven samples except RB08, which was seriously contaminated by chromosomal DNA.

Evaluation of plasmid assembly efficiency

Apart from plasmid RB08, de novo assembly was successfully performed on eleven MDR plasmids samples by Canu. High quality sequences were obtained by using Unicycler combined with short reads data. About one to five plasmids, which ranged from 46Kb to 238Kb in length, were found in each sample, with a total of 21 plasmids being obtained from eleven samples (**Table 1**). To evaluate the accuracy of de novo assembly of rapid 1D sequencing data generated by the MinION nanopore, the RB01 sample was selected for comparison between pair-end

illumina sequencing data and nanopore sequencing data. Sequences of two plasmids, RB01(RB01-LZ135-CTX-128976 and RB01-LZ135-NDM-90845), were selected for evaluation of the nanopore reads quality (**Figure 2**). Without size selection during library preparation, the reads length ranged from 18 to 97206bp and the N50 was 6473bp. Based on the alignment of reads to two reference plasmids, the MinION nanopore long read identity was about 87%.

After obtaining the raw reads quality, plasmids obtained from de novo assembly by Canu based on long reads were compared to the reference plasmids by BLASTn. The overall identity of the completed plasmids was 97% identical to the reference plasmids; the difference was mainly due to deletions (mainly 1-5bp) in plasmids assembled by Canu, resulting in an overall sequence 3043bp and 1949bp shorter than RB01-LZ135-CTX-128976 and RB01-LZ135-NDM-90845 respectively. No major structural variations were observed between the two different de novo assembly methods (**Figure 3**), indicating that nanopore long reads can be used to accurately resolve the mosaic structures frequently found in plasmids.

Characterization of MDR plasmids

The number of resistance genes detectable among the twenty one plasmids sequenced in this study ranged from 0 to 12, insertion sequences from 1 to 10 and replicon genes from 1 to 4 (**Table 2, Figure 4**). This implied that the plasmids tested in this study had complex structures, the complete sequences of which were usually difficult to obtain by short reads sequencing technology due to the presence of numerous repetitive sequences.

To demonstrate the ability of nanopore long reads to resolve the complex structures of MDR plasmids, sample RB01 was investigated in details. Upon de novo assembly, two complete

plasmids were obtained and designated as RB01-LZ135-CTX-128976 and RB01-LZ135-NDM-90845 respectively. This sample was a clinical carbapenem-resistant *E. coli* strain harboring the $bla_{\text{CTX-M-15}}$ and $bla_{\text{NDM-5}}$ gene, which was reported previously[8].

In the IncFII type plasmid RB01-LZ135-NDM-90845, which was 90845bp in length, there was a MDR mosaic region composed of Tn*3* transposon containing the $bla_{\text{TEM-1}}$ and *rmtB* genes, and IS*26*-IS*Aba125*- $bla_{\text{NDM-5}}$ –ble_{MBL}-*traF*-*tat*-IS*CR1*-*sul1*- *qacEdelta1*-*aadA2*-*dfrA12*-*intI1*-IS*26*. Intriguingly, the latter fragment was duplicated in a tandem repeat format (**Figure 5a**). Online BLASTn of this $bla_{\text{NDM-5}}$-bearing plasmid in the NCBI database showed that it was highly similar to the plasmid pMC-NDM, which was recovered from a metallo-beta-lactamase-producing *E. coli* strain in Poland (GeRBank no. HG003695), with 99% identity at 97% coverage. The two major differences include existence of tandem repeats and a region replacement (**Figure 5a**). The $bla_{\text{CTX-M-15}}$ bearing plasmid RB01-LZ135-CTX-128976 was 128976bp in length and had a conserved structure similar to one found in plasmid pECY55, which was harbored by a previously reported *E.coli* strain (GeRBank no. KU043115), with 99% identity at 97% coverage region. The MDR region harboring *tetA*, aac(6')-Ib-cr, $bla_{\text{OXA-1}}$, $bla_{\text{CTX-M-15}}$, *dfrA17*, *aadA5*, *sul1*, *chrA* and *mph*(A) was shared by these two plasmids, and two group II introns were found inserted in the backbone compared to pECY55 (**Figure 5b**). Detailed analysis of longest reads after BWA MEM alignment showed that two long reads spanned the plasmid RB01-LZ135-NDM-90845 end-to-end, and another two long reads could be ligated to generate plasmid RB01-LZ135-CTX-128976 (**Figure 5c** and **5d**). This is the first case in which whole plasmid sequence could be generated by only a single read.

Discussion

The advent of next-generation sequencing technologies revolutionizes the study mode in genomic research[9]. Specifically, it has tremendously facilitated molecular epidemiology studies and research on the diversity and evolution of antimicrobial resistance-encoding elements from both clinical and basic research perspectives[4,10]. Although it is feasible to assess the distribution of resistance genes among single bacterial or metagenomic samples with traditional short reads data, constructing the entire plasmid and chromosome maps that depict the specific location of resistance genes is of vital importance in investigating the evolution features of such genes and track the evolution and transmission routes of MDR plasmids[4,11]. The availability of long read sequencing technologies such as single-molecular real-time (SMRT) and MinION nanopore sequencing has shed light for development of efficient approaches to assemble complete genomes with numerous repetitive elements[12,13]. Owning to high cost and complex library preparation of SMRT technology, it cannot be commonly utilized in clinical settings and basic molecular laboratories although this technology has been commercially available for more than five years. On the contrary, the recently available portable MinION nanopore sequencing technology offers the opportunity to be used anywhere as long as a laptop computer is available. In this study, we evaluated the possibility of MinION nanopore sequencing technology to resolve the mosaic MDR plasmids with the latest R9.4 chemistry.

With the rapid barcoding sequencing kit, complete sequences of twenty one plasmids harbored by eleven samples could be successfully generated within few days (**Figure 6**). Although de novo assembly of only nanopore long reads by Canu exhibited a relatively low quality of only 97% identity to the reference sequences, the assembled plasmids were found to possess high quality structural skeletons with correct arrangements of various mobile elements. With Illumina

short read data, high quality plasmids could be obtained by Unicycler which involved three steps: contigs construction with short reads, scaffolding of contigs with long reads and polishing with short reads. Analysis of the two MDR plasmids in sample RB01 opened up an important observation that single long read can cover the complete plasmid; this finding indicated that the entire plasmid can be sequenced without interruption. In this case, de novo assembly was not necessary since several long reads may cover the whole plasmid. The first antibiotic resistance island resolved by MinION nanopore sequencing was reported in 2015[7].To the best of our knowledge, this is the first report of complete MDR plasmid sequencing without the need to assemble sheared fragments. It should be noted that, although only a few long reads were found to cover the entire plasmid, they were sufficient to cover all the repetitive sequences in the MDR plasmids. With further improvement in MinION sequencing, a plasmid being sequenced end-to-end as a single molecule will become possible in the near future.

Another advantage of MinION sequencing is that it allows an ongoing sequencing run to halt when sufficient data have been generated, saving time and most importantly the flow cell, which accounts for a significant portion of the cost of MinION sequencing. As a result, the flow cell can be reused several times until most of nanopore pores have lost activity. In this work, we finished the run in eight hours, during which we have generated sufficient data for assembling the complete plasmid sequences. Furthermore, the same flow cell was reused in another run and the data generated were of a quality similar to that of the first run (data not shown). The standard MinKNOW protocol involves running the flow cells for 48 hours. If one flow cell can accommodate 3 runs, each lasting for 8, 10 and 12 hours respectively, it infers that 36 MDR plasmid samples can be sequenced in one flow cell using the rapid barcode kit, leading to significant reduction in the cost of producing complete plasmid sequences.

As an extrachromosomal element, plasmids play a dominant role in dissemination of antibiotic resistance genes, virulence genes and other functional genes [14,15]. Obtaining complete plasmid sequences in a wide range of clinical isolates collected over a prolonged period enable in depth studies of plasmid evolution and adaptation, the underlying mechanisms of transmission of resistance genes, as well as tracking major antibiotic resistant pathogenic bacterial strains [5,15,16]. The workflow presented in this work offers for the first time the opportunities to perform these studies in a rapid, cost effective and user-friendly manner.

Methods

Bacterial MDR plasmids extraction

In order to evaluate the efficiency of MDR plasmid sequencing, we selected twelve MDR plasmid-bearing strains including *E. coli*, *Salmonella spp*., *V. parahaemolyticus* and *K. pneumoniae* for plasmids extraction (**Table 1**). Overnight cultures (100 mL) were harvested and subjected to plasmid extraction by using the QIAGEN Plasmid Midi Kit. The extracted plasmids were dissolved in ultrapure distilled water and concentrations were measured by Qubit 3.0 Fluorometer with dsDNA BR Assay Kit (Invitrogen). The plasmids were stored in -20℃ until library preparation.

MinION library preparation and sequencing

Library preparation was performed using Rapid Barcoding Sequencing kit (SQK-RBK001) according to the standard protocol provided by the manufacturer (Oxford Nanopore). Briefly, 7.5μL plasmid templates were combined with 2.5μL Fragmentation Mix Barcode (one barcode for each sample). The mixtures were incubated at 30℃ for 1 minute and at 75℃ for 1 minute. The barcoded libraries were pooled together with designated ratios in 10μL (**Table 1**). 1μL of RAD (Rapid 1D Adapter) was added to the pooled library and mixed gently. 0.2μL of Blunt/TA Ligase Master Mix was added and incubated for 5 minutes at room temperature. The constructed library was loaded into the Flow Cell R9.4 (FLO-MIN106) on a MinION device and run with SQK-RBK001_plus_Basecaller script of MinKNOW 1.5.12 software. The run was stopped after 8 hours and the flow cell was washed by a Wash Kit (EXP-WSH002) and stored in 4℃ for later use.

Illumina sequencing

To obtain high quality short reads data, pair-end (2×150bp) libraries were prepared by the focused acoustic shearing method, the NEBNext Ultra DNA Library Prep Kit and the Multiplex Oligos Kit for Illumina (NEB). The libraries were quantified by employing qPCR with P5-P7 primers, and pooled together and sequenced on the NextSeq 500 platform according to the manufacturer's protocol (Illumina).

Basecalling, demutiplexing, assembly of complete plasmid sequences and data analysis

Although local basecaller script was used during the run, there were still a small amount of reads which skipped being basecalled due to high speed generation of raw data. Albacore basecalling software (version 1.0.3, Oxford Nanopore) was used to generate fast5 files harboring 1D DNA sequence from fast5 files with only raw data in the tmp folder. Also, read_fast5_basecaller.py script in Albacore was used to demultiplex the twelve samples from basecalled fast5 files (except the files in fail folder) based on the twelve barcodes in SQK-RBK001. Poretools toolkit was utilized to extract all the DNA sequences from fast5 to fasta format among the twelve samples respectively[17]. Canu assembly tool (v1.3) was used to perform de novo assembly of complete plasmid sequences based on nanopore 1D long reads in three consecutive stages including correction, trimming and assembly[18]. The parameter genomeSize was set at 0.5m, 1m, 2m and 4m respectively to optimize the assembly results. High quality complete plasmids were constructed by hybrid de novo assembly of illumina short reads and nanopore long reads data using the Unicycler v0.3 tool (https://github.com/rrwick/Unicycler). NanoOK was adopted to evaluate the quality of nanopore long reads[19]. BWA MEM was used to align long reads against reference plasmids and visualized by IGV tool[20].

To assess the distribution of resistance genes, mobile elements and replicon genes, the corresponding databases were downloaded and BLASTn was performed among the finished plasmids. The result was visualized by in-house R script. Easyfig was utilized to compare the detailed structure of the MDR plasmids[21].

Availability of supporting data

Raw MinION sequencing data was deposited in figshare website(https://figshare.com/s/0e6b793aff1e790136e7). The twenty one plasmid sequences of the twelve samples were included as supplementary data. The two plasmids in sample RB01 were deposited in NCBI database with pending accession no.###. The two plasmids assembled by only MinION nanopore long reads in sample RB01 were also attached as supplementary data for reference.

Abbreviations

AMR: antimicrobial resistance; BLAST: basic local aligment tool; MDR: Multi-drug resistance; NCBI: National center for biotechnology information; ONT: Oxford nanopore technologies.

Author contributions

R.L. conceived and initiated the study. M. X., N. D and D. L. performed bacterial isolation and plasmids extraction. R.L., X. Y. and M. H. W. performed MinION and Illumina sequencing and data analysis. R.L. wrote the first draft of the manuscript. E. W. C. revised the manuscript. S.C. supervised the whole project.

Competing interests

The authors declare no competing financial interests.

Acknowledgements

This research was supported by the Chinese National Key Basic Research and Development (973) Program (2013CB127200) and Hong Kong Research Grant Council Collaborative Research Fund (C7038-15G).

Reference

1 Holmes, A. H. *et al.* Understanding the mechanisms and drivers of antimicrobial resistance. *Lancet* **387**, 176-187, doi:10.1016/S0140-6736(15)00473-0 (2016).

2 Marston, H. D., Dixon, D. M., Knisely, J. M., Palmore, T. N. & Fauci, A. S. Antimicrobial Resistance. *JAMA* **316**, 1193-1204, doi:10.1001/jama.2016.11764 (2016).

3 Smillie, C., Garcillan-Barcia, M. P., Francia, M. V., Rocha, E. P. C. & de la Cruz, F. Mobility of Plasmids. *Microbiol Mol Biol R* **74**, 434-452, doi:Doi 10.1128/Mmbr.00020-10 (2010).

4 Beatson, S. A. & Walker, M. J. Microbiology. Tracking antibiotic resistance. *Science* **345**, 1454-1455, doi:10.1126/science.1260471 (2014).

5 Conlan, S. *et al.* Single-molecule sequencing to track plasmid diversity of hospital-associated carbapenemase-producing Enterobacteriaceae. *Sci Transl Med* **6**, 254ra126, doi:10.1126/scitranslmed.3009845 (2014).

6 Bayliss, S. C., Hunt, V. L., Yokoyama, M., Thorpe, H. A. & Feil, E. J. The use of Oxford Nanopore native barcoding for complete genome assembly. *Gigascience*, doi:10.1093/gigascience/gix001 (2017).

7 Ashton, P. M. *et al.* MinION nanopore sequencing identifies the position and structure of a bacterial antibiotic resistance island. *Nat Biotechnol* **33**, 296-300, doi:10.1038/nbt.3103 (2015).

8 Huang, Y. *et al.* Widespread Dissemination of Carbapenem-Resistant Escherichia coli Sequence Type 167 Strains Harboring blaNDM-5 in Clinical Settings in China. *Antimicrobial agents and chemotherapy* **60**, 4364-4368, doi:10.1128/AAC.00859-16 (2016).

9 Goodwin, S., McPherson, J. D. & McCombie, W. R. Coming of age: ten years of next-generation sequencing technologies. *Nat Rev Genet* **17**, 333-351, doi:10.1038/nrg.2016.49 (2016).

10 Punina, N. V., Makridakis, N. M., Remnev, M. A. & Topunov, A. F. Whole-genome sequencing targets drug-resistant bacterial infections. *Hum Genomics* **9**, 19, doi:10.1186/s40246-015-0037-z (2015).

11 Ashton, P. M. *et al.* MinION nanopore sequencing identifies the position and structure of a bacterial antibiotic resistance island. *Nature Biotechnology* **33**, 296-+, doi:10.1038/nbt.3103 (2015).

12 Chin, C. S. *et al.* Nonhybrid, finished microbial genome assemblies from long-read SMRT sequencing data. *Nat Methods* **10**, 563-569, doi:10.1038/nmeth.2474 (2013).

13 Cao, M. D. *et al.* Scaffolding and completing genome assemblies in real-time with nanopore sequencing. *Nat Commun* **8**, 14515, doi:10.1038/ncomms14515 (2017).

14 Johnson, T. J. & Nolan, L. K. Pathogenomics of the virulence plasmids of Escherichia coli. *Microbiology and molecular biology reviews : MMBR* **73**, 750-774, doi:10.1128/MMBR.00015-09 (2009).

15 Conlan, S. *et al.* Plasmid Dynamics in KPC-Positive Klebsiella pneumoniae during Long-Term Patient Colonization. *MBio* **7**, doi:10.1128/mBio.00742-16 (2016).

16 Porse, A., Schonning, K., Munck, C. & Sommer, M. O. Survival and evolution of a large multidrug resistance plasmid in new clinical bacterial hosts. *Mol Biol Evol*, doi:10.1093/molbev/msw163 (2016).

17 Loman, N. J. & Quinlan, A. R. Poretools: a toolkit for analyzing nanopore sequence data. *Bioinformatics* **30**, 3399-3401, doi:10.1093/bioinformatics/btu555 (2014).

18 Koren, S. *et al.* Canu: scalable and accurate long-read assembly via adaptive k-mer weighting and repeat separation. *Genome Res* **27**, 722-736, doi:10.1101/gr.215087.116 (2017).

19 Leggett, R. M., Heavens, D., Caccamo, M., Clark, M. D. & Davey, R. P. NanoOK: multi-reference alignment analysis of nanopore sequencing data, quality and error profiles. *Bioinformatics* **32**, 142-144, doi:10.1093/bioinformatics/btv540 (2016).

20 Thorvaldsdottir, H., Robinson, J. T. & Mesirov, J. P. Integrative Genomics Viewer (IGV): high-performance genomics data visualization and exploration. *Brief Bioinform* **14**, 178-192, doi:10.1093/bib/bbs017 (2013).

21 Sullivan, M. J., Petty, N. K. & Beatson, S. A. Easyfig: a genome comparison visualizer. *Bioinformatics* **27**, 1009-1010, doi:DOI 10.1093/bioinformatics/btr039 (2011).

Table 1. Technical data of twelve MDR plasmid samples used in the single multiplexed MinION run.

Samples	Marker genes	Species	Plasmid profile[a]	7.5μL(ng)[b]	volume (μl)[c]	quantity(ng)[d]
RB01	*bla*$_{\text{NDM-5}}$	*Escherichia coli*	150kb ,100kb	750	0.8	60
RB02	*bla*$_{\text{NDM-5}}$	*Escherichia coli*	160kb,135kb,100kb, 60kb, 40kb	2010	0.4	160.8
RB03	*bla*$_{\text{NDM-1}}$	*Escherichia coli*	330kb,60kb	259.5	1.1	20.76
RB04	*bla*$_{\text{NDM-1}}$	*Escherichia coli*	110kb, 130kb, 230kb	937.5	0.7	75
RB05	*bla*$_{\text{CTX-M-15}}$	*Escherichia coli*	150kb	484.5	0.8	38.76
RB06	*bla*$_{\text{CTX-M-15}}$	*Escherichia coli*	250kb	270	1	21.6
RB07	*bla*$_{\text{CTX-M-15}}$	*Vibrio parahaemolyticus*	120kb	654	0.8	52.32
RB08	*bla*$_{\text{CTX-M-3}}$, *bla*$_{\text{TEM-1}}$	*salmonella spp*	340kb	885	0.8	70.8
RB09	*bla*$_{\text{KPC-2}}$	*Escherichia coli*	70kb	639	0.8	51.12

RB10	*bla*$_{KPC-2}$	*Escherichia coli*	100kb,130kb	346.5	1.1	27.72
RB11	*bla*$_{KPC-2}$	*Klebsiella Pneumoniae*	240kb	1125	0.8	90
RB12	*bla*$_{KPC-2}$	*Escherichia coli*	120kb, 100kb	495	0.9	39.6

[a] Plasmid profile was determined by S1 nuclease Pulsed-field gel electrophoresis(PFGE); the sizes of the plasmids were roughly estimated based on S1-PFGE.

[b] The input quantities of plasmid DNA in 7.5μL library preparation.

[c] The volume of each sample in the pooled library.

[d] The actual quantity of DNA of each sample used in MinION sequencing.

Table 2. Overview of structure and genetic characteristics of twenty one MDR plasmids recovered from eleven samples

Plasmids[a]	Size (bp)	Structural status	No. of Resistance genes	No. of Insertion sequences	No. of Replicon genes
RB01-LZ135-CTX-128976	128976	Circular	8	5	2
RB01-LZ135-NDM-90845	90845	Circular	5	2	1
RB02-JN105-IncF-TET-116277-N	116277	Circular	6	6	2
RB02-JN105-IncN-CTX-139496-N	142307	Circular	9	2	2
RB02-JN105-IncN-NDM6-55342	55342	Circular	3	3	1
RB02-JN105-IncX-NDM5-45823	45823	Circular	1	4	1
RB02-JN105-IncY-CTX-98443	98443	Circular	0	1	1
RB03-WH96T-IncF-OXA-153088	153088	Circular	3	9	4
RB03-WH96T-IncN-NDM1-56215	56215	Circular	2	4	1

RB04-SZ584-1T-IncF-TET-114056	114065	Circular	7	6	2
RB04-SZ584-1T-IncX3-NDM1-56K-NC	55919	Linear	2	4	1
RB04-SZ584-1T-IncY-130821	130821	Circular	0	9	1
RB05-C267-IncA/C-CTX-166467	166467	Circular	10	3	1
RB06-C499-IncA/C-CTX-192739	192739	Circular	11	3	1
RB07-vb0506-IncA/C-CTX-133742	133742	Circular	6	2	1
RB09-IncN-KPC-68571	68571	Circular	7	6	1
RB10-29KPC-IncF-TET-136532	136532	Circular	12	6	3
RB10-29KPC-IncY-KPC-98K-N	95908	Circular	1	2	1
RB11-IncF-IncHI-KPC-238153	238153	Circular	2	10	2
RB12-74T-KPC-IncF-115K-N	115689	Circular	0	6	4

RB12-74T-KPC-IncN-IncX1-KPC-108K-N	107969	Circular	5	4	3

[a] Plasmid names ending with letter N indicated that the plasmids could be assembled by Canu based on MinION nanopore reads, but cannot be assembled using hybrid assembly strategy with Unicycler. Plasmid name ending with NC indicated it was assembled incompletely.

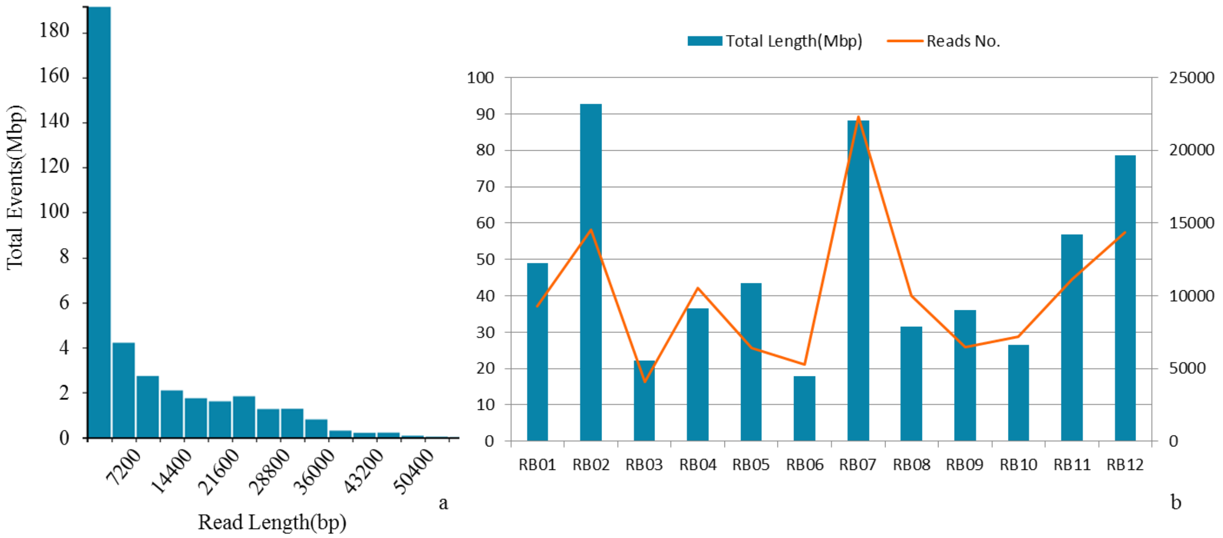

Figure 1. Statistics of an eight-hours MinION nanopore sequencing run using the Rapid Barcoding Sequencing Kit. a, distribution of reads length and data volume generated by the MinION run in eight hours; b, total base length and reads number of the twelve samples after demultiplexing.

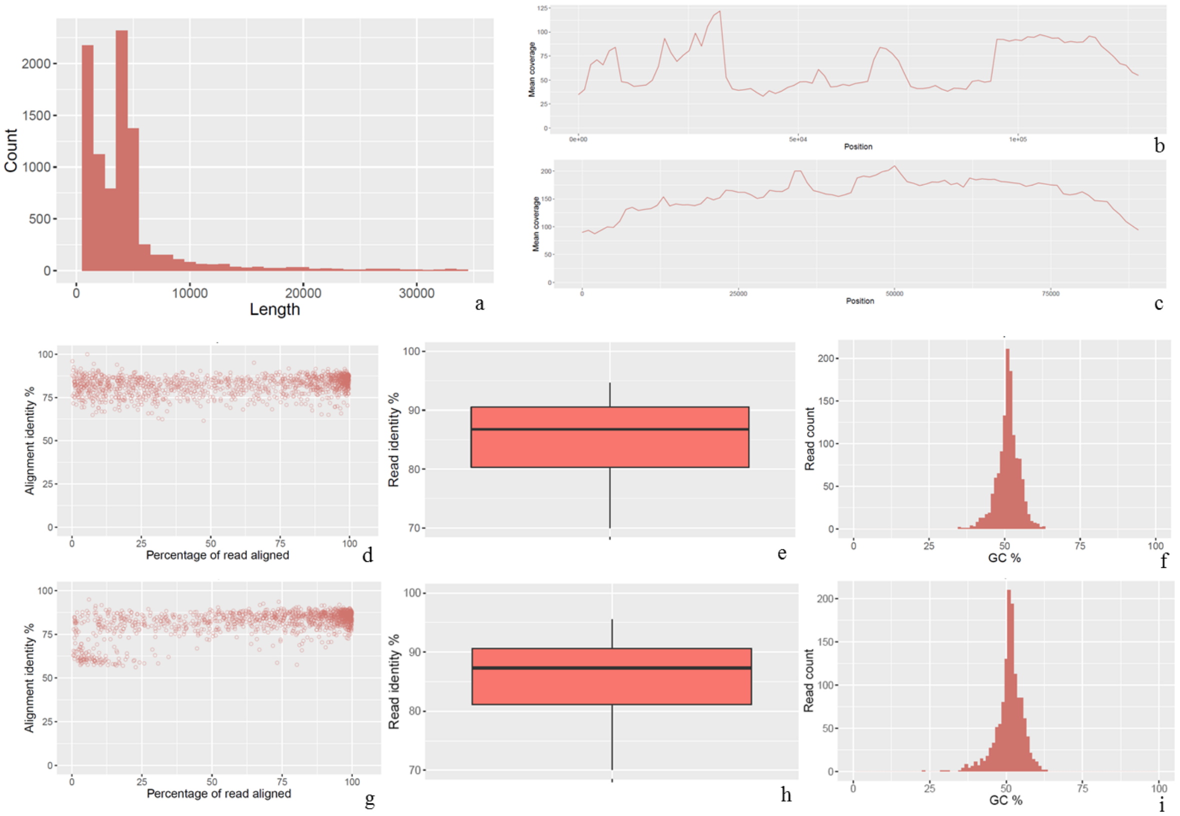

Figure 2. Evaluation of nanopore sequencing long reads quality with nanonet. a, reads counts along with reads length for sample RB01. All the raw reads could be retrieved from the deposited data online. b, nanopore reads coverage with RB01-LZ135-CTX-128976 as reference. c, nanopore reads coverage with RB01-LZ135-NDM-90845 as reference. d, e and f, alignment identity and GC distribution for reads aligned with RB01-LZ135-CTX-128976. g, h and I, alignment identity and GC distribution for reads aligned with RB01-LZ135-NDM-90845.

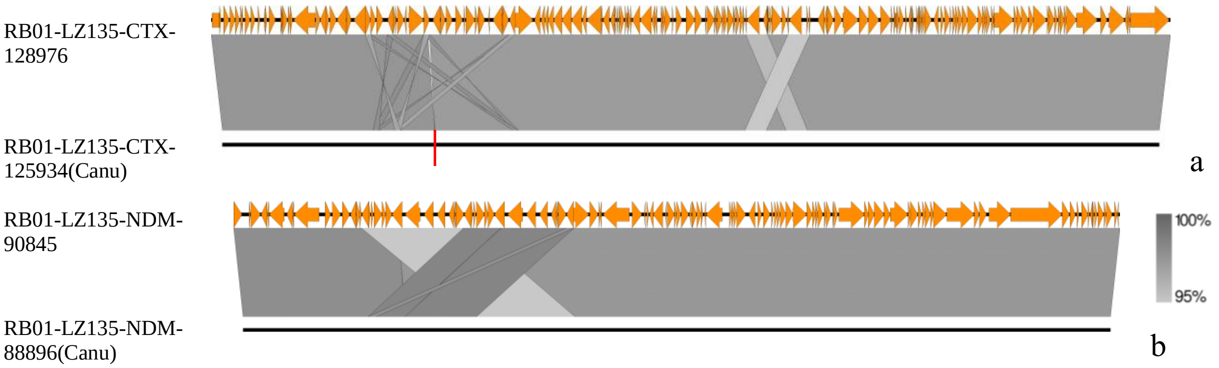

Figure 3. Linear alignment of reference plasmids and corresponding plasmids assembled by Canu based only on MinION nanopore long reads of sample RB01. a, alignment of RB01-LZ135-CTX-128973 and RB01-LZ135-CTX-125934 (Canu). A large deletion that existed in the plasmid RB01-LZ135-CTX-125934 is marked by a red vertical line. b, alignment of RB01-LZ135-NDM-90845 and RB01-LZ135-NDM-88896(Canu). The crossed alignment region indicates that the $bla_{\text{NDM-5}}$ region was duplicated. The two plasmids sequences could be retrieved from the supplementary data 1.

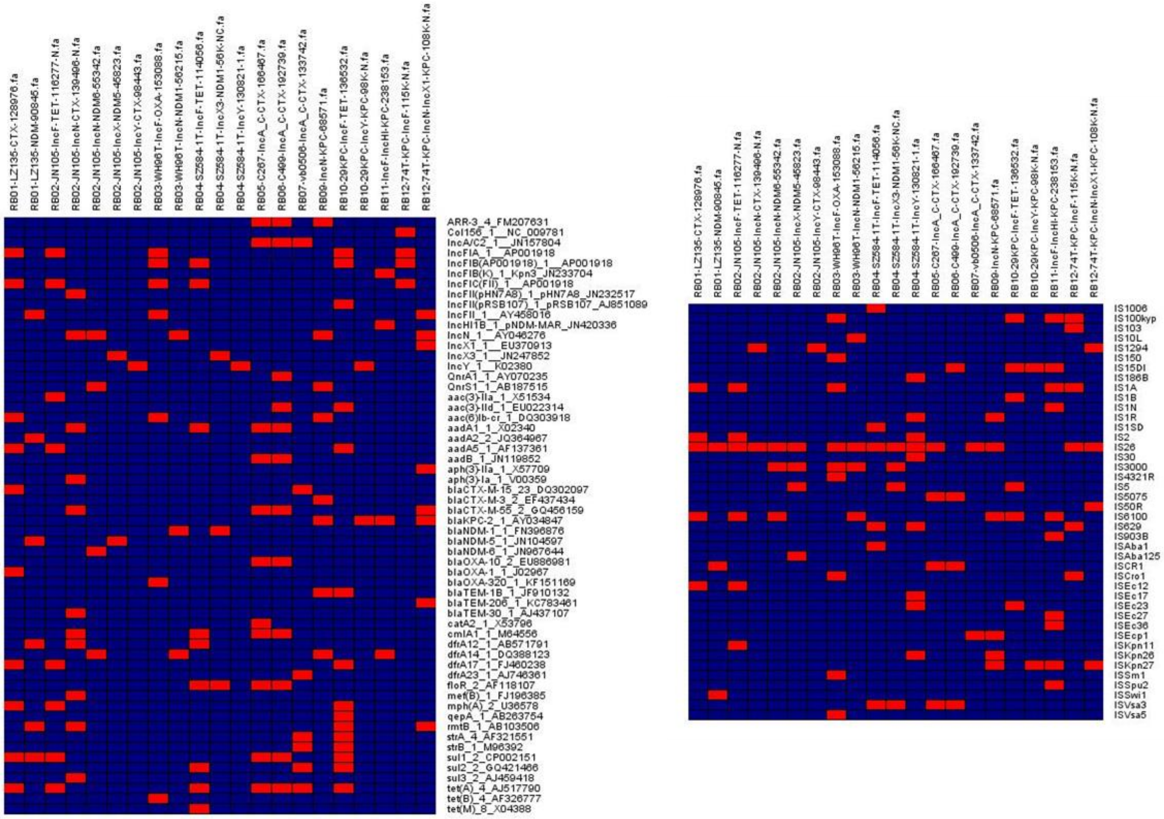

Figure 4. Distribution of resistance genes, replicon genes and insertion sequences among twenty-one plasmids. Red boxes indicate the presence of corresponding genes, and blue boxes indicate absence of the corresponding genes. The twenty one plasmids sequencing could be retrieved from the supplementary data 2.

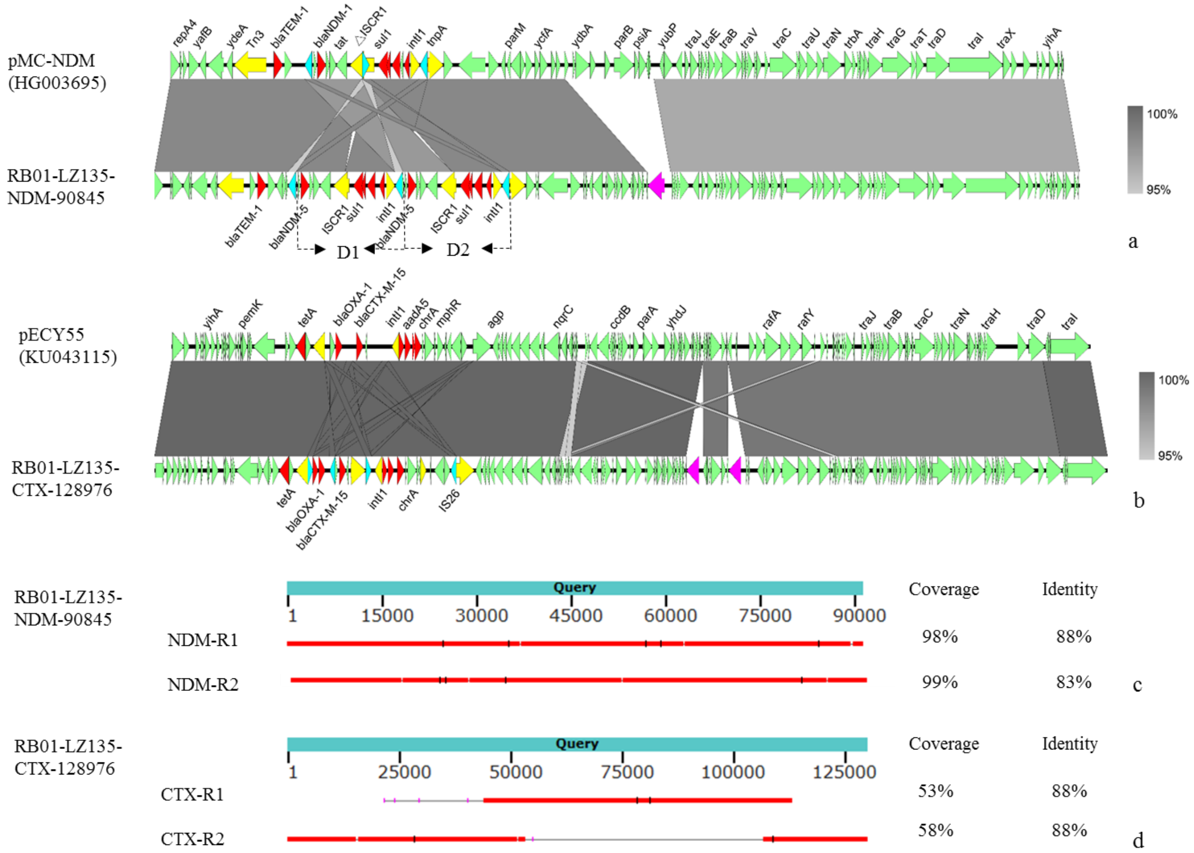

Figure 5. Alignment of plasmids in RB01 against those harboring similar structures in NCBI database and single plasmid sequence representation. a, alignment between pMC-NDM and RB01-LZ135-NDM-90845. The resistance genes were highlighted in red, transposase genes in yellow, IS*26* in cyan, group II intron gene in pink and other CDSs in light green. The sequence contained a large duplication region (ca.10kbp) designated as D1 and D2, each harboring a class 1 integron and a *bla*$_{\text{NDM-1}}$ cluster; b, alignment between pECY55 and RB01-LZ135-CTX-128976. The CDSs were labelled according to the labeling scheme in Fig. a. The same group II intron gene were inserted and duplicated in RB01-LZ135-CTX-128976 and compared with pECY55. c, BLASTn of two MinION long reads against RB01-LZ135-NDM-90845. The results indicated that the whole plasmid could be sequenced end-to-end. d, BLASTn

of two MinION long reads against RB01-LZ135-CTX-128976. The results indicated that two MinION long reads could cover the entire plasmid. The four long reads could be retrieved from the supplementary data 3.

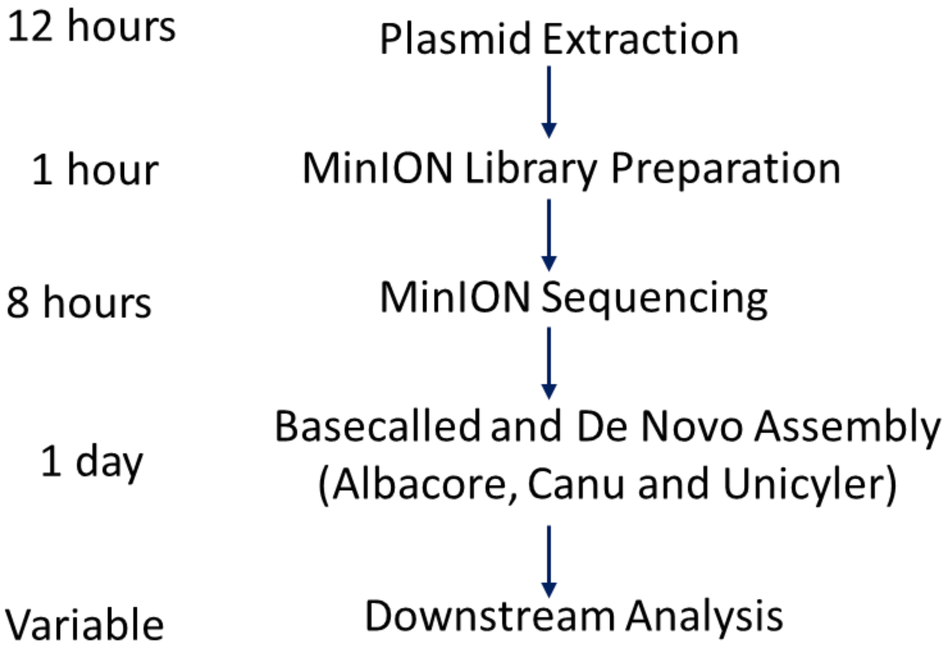

Figure 6. Workflow and timeframe overview of MinION sequencing and assembly process. This workflow was based on the rapid barcoding sequencing kit which could pool twelve samples in a single run. The time for basecalling and de novo assembly depended on the computational performance of computer utilized, and Illumina short reads were needed if Unicycler was used to obtain high quality assembled plasmids.

Click here to access/download
Supplementary Material
supplementary data 1-RB01 plasmids by Canu.fa

Click here to access/download
Supplementary Material
supplementary data 2-twenty one plasmids.fa

Click here to access/download
Supplementary Material
supplementary data 3-four long reads.fa

Click here to access/download
Supplementary Material
RB01-LZ135-CTX-128976-GBK(For review).gbk

Click here to access/download
Supplementary Material
RB01-LZ135-NDM-90845-GBK(For review).gbk
