# Supplementary material for: Efficient generation of complete sequences of MDR-encoding plasmids by rapid assembly of MinION barcoding sequencing data
Source: Gigascience. 2018 Jan 9;7(3):gix132. doi: 10.1093/gigascience/gix132 (PMC5848804; doi:10.1093/gigascience/gix132)
Supplement: GIGA-D-17-00150_Revision_1.pdf [file gix132_giga-d-17-00150_revision_1.pdf]

GigaScience

Efficient generation of complete sequences of MDR-encoding plasmids by rapid assembly of MinION barcoding sequencing data

--Manuscript Draft--

**Manuscript Number:**	GIGA-D-17-00150R1
**Full Title:**	Efficient generation of complete sequences of MDR-encoding plasmids by rapid assembly of MinION barcoding sequencing data
**Article Type:**	Research
**Funding Information:**	973 (2013CB127200) — Dr Sheng CHEN<br>CRF (C7038-15G) — Dr Sheng CHEN<br>CRF (C5026-16G) — Dr Sheng CHEN
**Abstract:**	Background: Multidrug resistance (MDR)-encoding plasmids are considered major molecular vehicles responsible for transmission of antibiotic resistance genes among bacteria of the same or different species. Delineating the complete sequences of such plasmids could provide valuable insight into the evolution and transmission mechanisms underlying bacterial antibiotic resistance development. However, due to the presence of multiple repeats of mobile elements, complete sequencing of MDR plasmids remains technically complicated, expensive and time-consuming.<br><br>Results: Here, we demonstrate a rapid and efficient approach to obtain multiple MDR plasmid sequences through the use of the MinION nanopore sequencing platform, which is incorporated in a portable device. By assembling the long sequencing reads generated by a single MinION run according to a rapid barcoding sequencing protocol, we obtained the complete sequence of twenty-one plasmids harbored by multiple bacterial strains. Importantly, single long reads covering a plasmid end-to-end were recorded, indicating that de novo assembly may be unnecessary if the single reads exhibit high accuracy.<br><br>Conclusions: This workflow represents a convenient and cost-effective approach for systematic assessment of MDR plasmids responsible for treatment failure of bacterial infections, offering the opportunity to perform detailed molecular epidemiological studies to probe the evolutionary and transmission mechanisms of MDR-encoding elements.
**Corresponding Author:**	Sheng CHEN<br>Hong Kong Polytechnic University<br>Hung Hom, HONG KONG
**Corresponding Author Secondary Information:**	
**Corresponding Author's Institution:**	Hong Kong Polytechnic University
**Corresponding Author's Secondary Institution:**	
**First Author:**	Ruichao Li
**First Author Secondary Information:**	
**Order of Authors:**	Ruichao Li<br>Miaomiao Xie<br>Ning Dong<br>Dachuan Lin<br>Xuemei Yang<br>Marcus Wong<br>Edward Chan

Powered by Editorial Manager® and ProduXion Manager® from Aries Systems Corporation

	Sheng CHEN
**Order of Authors Secondary Information:**	
**Response to Reviewers:**	Responses to reviewers<br><br>Reviewer reports:<br><br>Reviewer #1:<br>Q1. This study is about a method to obtain completed MDR-encoding plasmids while minimizing the turnaround time. The idea is to use MinION barcode sequencing on extracted plasmids and then use computational tools to assembly them. Although there are several studies conceived with similar approach (Sion C. Bayliss et al 2017, Ryan R. Wick et al 2017), they're focusing on completing the whole bacteria genomes rather than MDR-plasmids of interest alone. The question is how this study serves its purpose more efficient than aforementioned methods, when the turn-around time and cost are about similar?<br><br>Response: Our study describes an approach to obtain completed MDR-encoding plasmids in a cost-effective and and a much more efficient manner when compared with the previous methods (Illumina plus PCR mapping, PacBio, etc.). Although there have been a few studies on producing Oxford Nanopore long read data, the research focus, turn-around time and cost in the previous studies (Sion C. Bayliss et al 2017, Ryan R. Wick et al 2017) are quite different from ours.<br><br>Silon C. Bayliss and colleagues (Sion C. Bayliss et al 2017) reported the use of Oxford Nanopore native barcoding method for complete genome assembly. However, the method they used was native barcoding method, which takes more time and needs various costly consumables. Although they sequenced a multiplexed library of 12 samples, only one successfully assembled sample, namely Staphylococcus aureus MHO_00,1 was presented. Most importantly, the MinION 2D technology they used in the study has been replaced by 1D technology by Oxford Nanopore technologies. To some extent, the workflow in their study was ‘old-fashioned’. Compared with the pilot study conducted by Silon C. Bayliss, our study presents a much more efficient approach to assemble MDR-encoding plasmids with less lab equipments and consumables. Also, the flow cell was found to be reusable for at least three multiplexed libraries, which result in dramatic decrease of cost incurred. Users will find these information highly practical.<br><br>Ryan R. Wick and colleagues (Ryan R. Wick et al 2017) developed a very useful tool Unicycler, which is a new tool for assembling bacterial genomes from a combination of short and long reads. In their study, they focus on evaluation of the tool performance based on simulated and real data compared with other assembly tools. In our study, we utilize this useful Unicycler tool to perform hybrid assembly and generate highly accurate data in specially designed workflow for finishing MDR-encoding plasmids, which is a read-to-use approach (from samples to complete sequences) highly suitable for conventional molecular labs. The research purposes are different between ours and previous studies. With the advance of Oxford Nanopore long reads accuracy, hybrid assembly may not be necessary and long reads will be sufficient for generating high quality complete sequences with Canu or other tools.<br><br>Q2. The plasmids are constructed eventually by using hybrid assembly with Illumina data so the idea of using MinION alone is not valid. Instead a post-polishing step can be done for Canu assembly to improve its quality. Have authors tried to do that? If Illumina data involved, in combination with real-time property of MinION (mentioned at line 86-87 and last paragraph of Dicussion), should it be a hybrid real-time assembly a more reasonable way to go?<br><br>Response: The reason for constructing full plasmid sequences by using hybrid assembly combining Illumina short read data and Oxford Nanopore long read data is the low accuracy of long read data. The accuracy of single long read is determined to be about 87% using a reference sequence. With Canu assembly along with the correction process based only on the long reads, the assembled plasmids can be 97% identical to the reference plasmids. Until now, Nanopore long read data cannot

Powered by Editorial Manager® and ProduXion Manager® from Aries Systems Corporation

generate high quality assembled result without polishing with high quality short read data. This error is largely due to the inability of the present MinION platform to resolve the long homopolymers issue.

The hybrid real-time assembly is a great idea to obtain complete genome assemblies, and this idea has been validated with npScarf tool (Minh D. Cao et al. 2017). The hybrid real-time assembly of MDR-encoding should be feasible. However, hybrid real-time assembly is more appropriate in applications in clinical settings. Actually, we have tried to utilize jspsa tools for real-time analysis our data and found that the process required professional knowledge in bioinformatics and is too difficult to be used in conventional labs without dedicated bioinformatician. However, the workflow presented in our study is easy to be applied in most labs using laptops, without professional bioinformaticians. The time span of our workflow is ideal for studies on MDR-encoding plasmids (data generated within a few days), so a hybrid real-time assembly may not be necessary. However, we have also discussed the possible application of tools like npScarf for real-time assembly in the Discussion section.

Q3. Nanopore raw and Illumina data should be made public also; try to put everything (Illumina, Nanopore, plasmid assemblies...) into one location (e.g. a project in NCBI)

Response: We have deposited all the raw read data in the NCBI database with a project number PRJNA398365. All the supplemented data was deposited in the GigaDB repository.

4. Minor: table 1 samples ID (column 1) and table 2 plasmid size (column 2) shouldn't be broken.

Response: The formats of table 1 (column 1) and table 2 (column 2) have been revised to remove the broken lines.

Reviewer #2:

Q1: The authors present the use of oxford nanopore technology to sequence 12 plasmid extract samples and assemble these 21 complete plasmid sequences. They go on to compare the Canu derived assemblies with a hyrbrid Illumina assembly generated with UniCycler. The paper presents the methodologies used and the results clearly and this is a useful example of the use of this technology. The paper requires considerable edits in terms of use of the English language and clarity of phrase.

Response: The English language has been revised throughout the manuscript. All the phrases are also rechecked to keep clarity.

Abstract.
Q2: Line 34 - repetitive use of the name MinION.

Response: The sentence has been revised into “Here, we demonstrate a rapid and efficient approach to obtain multiple MDR plasmid sequences through the use of the MinION nanopore sequencing platform, which is incorporated in a portable device.”

Q3: Line 41 - What is novel here?

Response: The workflow presented in this study is novel in several aspects: (i) the application of Oxford Nanopore rapid barcoding method in MDR-encoding plasmids is investigated for the first time; (ii) a flow cell can be re-used for plasmids samples which do not require a lot of sequencing data for assembly; (iii) Oxford Nanopore long read can cover all the plasmid sequence, hence de novo assembly may not necessary. However, due to the rapid development of sequencing technologies and related workflows, we delete the word “novel” here.

Introduction.
Q4: Line 51 - What do you mean by drivers in the is context. The main driver is overuse of antibiotics? Do you mean mechanisms?

Response: Drivers indicate the mechanisms responsible for widespread dissemination

Powered by Editorial Manager® and ProduXion Manager® from Aries Systems Corporation

	of AMR. To be concise, the sentence has been revised into “Identification of key mechanisms responsible for AMR transmission is crucial to combat the threats imposed by AMR.”<br><br>Q3: Line 59 - I guess you mean Illumina and not Sanger in this context<br><br>Response: The conventional method to obtain complete plasmid sequences is to conduct de novo assembly using short read data (Illumina etc.) to generate contigs. PCR mapping based on the Sanger sequencing method will be used to close the gaps to finish the complete sequences. This sentence has been revised to enhance clarity.<br><br>Q4: Line 67 - Very vague sentence - what do you mean by advent of library preparation techniques and data analysis tools<br><br>Response: We tried to emphasize that the library preparation techniques and data analysis tools developed for Oxford Nanopore technologies in this study make it a very suitable technique for rapid and accurate MDR plasmid sequencing. This sentence has been revised.<br><br>Results.<br>Q5: Line 95 - Use of seriously is not good English in this context<br><br>Response: The word “seriously” was replaced by “severely”.<br><br>Q6: Line 99 - What do you mean by high quality sequences here? % > Q20/30?<br><br>Response: High quality sequences mean the assembled plasmid sequences, not the raw reads. The hybrid assembly using Unicycler, when combined with short reads data, can generate high quality assembled plasmid sequences (regarded as reference sequences), compared with the Illumina de novo assembly results.<br><br>Q7: Line 100 - Use of About not needed<br><br>Response: The word “About” was deleted.<br><br>Discussion<br>Q8: Line 108 - How were the reference plasmids selected / created?<br><br>Response: The reference plasmids refer to complete plasmids assembled by Unicycler. As Illumina data is regarded as data with high accuracy, plasmid sequences assembled by Unicycler using Illumina data are regarded as reference plasmids in this study.<br><br>Q9: Line 110 - This sentence doesn't make sense<br><br>Response: “After obtaining the raw reads quality” was deleted. The sentence was revised into “Complete plasmids sequences obtained from de novo assembly by Canu, based on long reads, were compared to the reference plasmids (assembled by Unicycler) using BLASTN.”<br><br>We would like to thank the reviewers for taking time to assess our manuscript and share their insightful comments and suggestions. Based on the constructive reports, we have modified the manuscript and provide point-by-point responses to the comments:
**Additional Information:**	
**Question**	**Response**
Are you submitting this manuscript to a special series or article collection?	No
**Experimental design and statistics**	Yes

Powered by Editorial Manager® and ProduXion Manager® from Aries Systems Corporation

Full details of the experimental design and statistical methods used should be given in the Methods section, as detailed in our Minimum Standards Reporting Checklist. Information essential to interpreting the data presented should be made available in the figure legends.<br><br>Have you included all the information requested in your manuscript?	
**Resources**<br><br>A description of all resources used, including antibodies, cell lines, animals and software tools, with enough information to allow them to be uniquely identified, should be included in the Methods section. Authors are strongly encouraged to cite Research Resource Identifiers (RRIDs) for antibodies, model organisms and tools, where possible.<br><br>Have you included the information requested as detailed in our Minimum Standards Reporting Checklist?	Yes
**Availability of data and materials**<br><br>All datasets and code on which the conclusions of the paper rely must be either included in your submission or deposited in publicly available repositories (where available and ethically appropriate), referencing such data using a unique identifier in the references and in the "Availability of Data and Materials" section of your manuscript.<br><br>Have you have met the above requirement as detailed in our Minimum Standards Reporting Checklist?	Yes

Powered by Editorial Manager® and ProduXion Manager® from Aries Systems Corporation

Manuscript
Click here to download Manuscript Efficient MDR plasmid sequencing R1.docx

Efficient generation of complete sequences of MDR-encoding plasmids by rapid assembly of MinION barcoding sequencing data

Ruichao Li[1,2], Miaomiao Xie[1], Ning Dong[1], Dachuan Lin[1,2], Xuemei Yang[1], Marcus Ho Yin Wong[1], Edward Wai-Chi Chan[2], Sheng Chen[1,2]*

[1] Shenzhen Key Lab for Food Biological Safety Control, Food Safety and Technology Research Center, Hong Kong PolyU Shen Zhen Research Institute, Shenzhen, P. R. China;

[2] The State Key Lab of Chirosciences, Department of Applied Biology and Chemical Technology, The Hong Kong Polytechnic University, Hung Hom, Kowloon, Hong Kong SAR;

Running title: Rapid assembly of plasmids by MinION sequencing data

Keywords: Multidrug resistance (MDR) plasmids, *de novo* assembly, nanopore sequencing, long reads

Abstract

Background: Multidrug resistance (MDR)-encoding plasmids are considered major molecular vehicles responsible for transmission of antibiotic resistance genes among bacteria of the same or different species. Delineating the complete sequences of such plasmids could provide valuable insight into the evolution and transmission mechanisms underlying bacterial antibiotic resistance development. However, due to the presence of multiple repeats of mobile elements, complete sequencing of MDR plasmids remains technically complicated, expensive and time-consuming.

Results: Here, we demonstrate a rapid and efficient approach to obtain multiple MDR plasmid sequences through the use of the MinION nanopore sequencing platform, which is incorporated in a portable device. By assembling the long sequencing reads generated by a single MinION run according to a rapid barcoding sequencing protocol, we obtained the complete sequence of twenty-one plasmids harbored by multiple bacterial strains. Importantly, single long reads covering a plasmid end-to-end were recorded, indicating that *de novo* assembly may be unnecessary if the single reads exhibit high accuracy.

Conclusions: This workflow represents a convenient and cost-effective approach for systematic assessment of MDR plasmids responsible for treatment failure of bacterial infections, offering the opportunity to perform detailed molecular epidemiological studies to probe the evolutionary and transmission mechanisms of MDR-encoding elements.

Introduction

The emergence and increasing prevalence of antimicrobial resistance (AMR) among bacterial pathogens pose increasing public health challenges worldwide by drastically reducing the number of antimicrobials that can be effectively used in treatment of bacterial infections [1, 2]. Identification of key mechanisms responsible for AMR transmission is crucial to combat the threats imposed by AMR. Plasmids, especially the MDR-encoding plasmids, are now considered a major vector that facilitates AMR transmission among bacteria via horizontal transfer [3, 4]. Delineating the full length of plasmids and genetic structures of other MDR-encoding mobile elements are vital for understanding how such elements undergo evolutionary changes and horizontal transmission, and adapt to new host [4]. However, due to the presence of numerous insertion sequences and other repetitive elements in MDR plasmids, it is often difficult and time-consuming to obtain the complete plasmid sequences by next-generation sequencing with the short reads and PCR mapping by Sanger sequencing. With the development of long read sequencing technology, tracking plasmid diversity by full assembly of plasmids has become possible [5]. To date, single-molecule, real-time sequencing (SMRT) can generate full-sequence plasmids. However, the huge cost and laborious library preparation procedure of this technology renders it inaccessible for most laboratories.

Recently, another long read sequencing technology based on the use of a portable MinION device has been available from Oxford Nanopore Technologies (ONT). Although the accuracy of reads generated by this technique is generally lower than that of short reads, it exhibits promising capability to generate complete chromosome and plasmid sequences [6, 7]. With the advance of library preparation techniques and data analysis tools, we found that this technology is feasible

for MDR plasmids sequencing. Here, we evaluated the feasibility of decoding the complete sequences of multiple MDR plasmids by the MinION nanopore sequencing technology, through a run with a reusable flow cell within a short time frame. This workflow shall enable laboratories equipped with only basic molecular biology techniques to perform detailed MDR plasmids analysis.

Data description

Raw long sequencing data collected after MinION run was de-multiplexed by Albacore basecalling software (v1.0.3) to generate fast5 files allocated into twelve samples. Poretools tool was used to extract reads with fasta format and preceded to *de novo* assembly and hybrid assembly with Canu (v1.3) and Unicycler (v0.3). At last, twenty-one plasmids were obtained with single MinION run data efficiently. The detailed procedures for data analysis were described in Methods.

Results

MinION workflow overview

Twelve MDR plasmids samples were prepared according to the MinION library construction protocols, followed by library sequencing. After eight hours of sequencing run, a total of 287,725 reads ranging from dozens to tens of thousands of bases in length were obtained, covering a total of 493Mbp (**Fig. 1a**). It was estimated that the data should be enough for *de novo* assembly; hence the run was stopped manually to save active nanopores for future use. The raw data was subjected to several stages of processing including base-calling, de-multiplexing, fasta sequence extraction and *de novo* assembly as stated in the Methods section. Upon de-multiplexing, a total of 121,584 reads were allocated into the twelve samples, which ranged from 5,273 to 22,319 in reads number and 18 to 93 Mbp in total (**Fig. 1b**). The reads were unsuccessfully base-called and unclassified reads during the de-multiplexing process were excluded from the assembly analysis. By optimizing the parameters of *de novo* assembly tool, we obtained the complete sequences of all the MDR plasmids recovered from eleven samples except RB08, which was severely contaminated by chromosomal DNA.

Evaluation of plasmid assembly efficiency

Apart from plasmid RB08, *de novo* assembly was successfully performed on eleven MDR plasmids samples by Canu. High quality assembled sequences were obtained using Unicycler combined with short reads data. One to five plasmids, which ranged from 46Kb to 238Kb in length, were found in samples, with a total of twenty-one plasmids being obtained from eleven samples (**Table 1**). To evaluate the accuracy of *de novo* assembly of rapid 1D sequencing data generated by the MinION platform, the RB01 sample was selected for comparison between pair-

end Illumina sequencing data and nanopore sequencing data. Sequences of two plasmids, RB01-LZ135-CTX-128976 and RB01-LZ135-NDM-90845, were selected for evaluation of the nanopore reads quality (**Fig. 2**). Without size selection during library preparation, the reads length ranged from 18 to 97206 bp and the N50 was 6473 bp. Based on the alignment of reads to two reference plasmids, the MinION nanopore long reads identity was about 87%.

Complete plasmid sequences obtained from *de novo* assembly by Canu based on long reads were compared to the reference plasmids (assembled by Unicycler) by BLASTN. The overall identity of the completed plasmids by Canu was 97% identical to the reference plasmids; the difference was mainly due to fabricated deletions in plasmids assembled by Canu, resulting in an overall sequence 3043 bp and 1949 bp shorter than RB01-LZ135-CTX-128976 and RB01-LZ135-NDM-90845 respectively. No major structural variations were observed between the two different *de novo* assembly methods (**Fig. 3**), indicating that nanopore long reads can be used to accurately resolve the mosaic structures frequently found in plasmids.

Characterization of MDR plasmids

The number of resistance genes detectable among the twenty-one plasmids sequenced in this study ranged from 0 to 12, insertion sequences from 1 to 10 and replicon genes from 1 to 4 (**Table 2, Fig. 4**). This implied that the plasmids tested in this study had complex structures, the complete sequences of which were usually difficult to obtain by short reads sequencing technology due to the presence of numerous repetitive sequences.

To demonstrate the ability of nanopore long reads to resolve the complex structures of MDR plasmids, sample RB01 was investigated in detail. Upon *de novo* assembly, two complete

plasmids were obtained and designated as RB01-LZ135-CTX-128976 and RB01-LZ135-NDM-90845 respectively. This sample was a clinical carbapenem-resistant *E. coli* strain harboring the *bla*$_{\text{CTX-M-15}}$ and *bla*$_{\text{NDM-5}}$ gene, which was reported previously [8].

In the IncFII type plasmid RB01-LZ135-NDM-90845, which was 90845 bp in length, there was a MDR mosaic region composed of Tn*3* transposon containing the *bla*$_{\text{TEM-1}}$ and *rmtB* genes, and IS*26*-IS*Aba125*-*bla*$_{\text{NDM-5}}$-*ble*$_{\text{MBL}}$-*traF*-*tat*-IS*CR1*-*sul1*-*qacEdelta1*-*aadA2*-*dfrA12*-*intI1*-IS*26*. Intriguingly, the latter fragment was duplicated in a tandem repeat format (**Fig. 5a**). Online BLASTN of this *bla*$_{\text{NDM-5}}$-bearing plasmid in the NCBI database showed that it was highly similar to the plasmid pMC-NDM, which was recovered from a metallo-beta-lactamase-producing *E. coli* strain in Poland (GenBank no. HG003695), with 99% identity at 97% coverage. The two major differences include existence of tandem repeats and a region replacement (**Fig. 5a**). The *bla*$_{\text{CTX-M-15}}$ bearing plasmid RB01-LZ135-CTX-128976 was 128,976 bp in length and had a conserved structure similar to one found in plasmid pECY55, which was harbored by a previously reported *E.coli* strain (GenBank no. KU043115), with 99% identity at 97% coverage region. The MDR region harboring *tetA*, *aac(6')-Ib-cr*, *bla*$_{\text{OXA-1}}$, *bla*$_{\text{CTX-M-15}}$, *dfrA17*, *aadA5*, *sul1*, *chrA* and *mph*(A) was shared by these two plasmids, and two group II introns were found inserted in the backbone compared to pECY55 (**Fig. 5b**). Detailed analysis of longest reads after BWA MEM alignment showed that two long reads spanned the plasmid RB01-LZ135-NDM-90845 end-to-end, and another two long reads could be aligned to generate plasmid RB01-LZ135-CTX-128976 (**Fig. 5c** and **5d**). This is the first case in which whole plasmid sequence could be generated by only a single read.

Discussion

The advent of next-generation sequencing technologies revolutionizes the study mode in genomic research [9]. Specifically, it has tremendously facilitated molecular epidemiology studies and research on the diversity and evolution of MDR-encoding elements from both clinical and basic research perspectives [4, 10]. Although it is feasible to assess the distribution of resistance genes among single bacterial or metagenomic samples with traditional short reads data, constructing the entire plasmid and chromosome maps that depict the specific location of resistance genes is of vital importance in investigating the evolution features of such genes and track the evolution and transmission routes of MDR plasmids [4, 11]. The availability of long read sequencing technologies such as SMRT and MinION nanopore sequencing has shed light for development of efficient approaches to assemble complete genomes with numerous repetitive elements [12, 13]. Owning to high cost and complex library preparation of SMRT technology, it cannot be commonly utilized in clinical settings and basic molecular laboratories although this technology has been commercially available for more than five years. On the contrary, the recently available portable MinION nanopore sequencing technology offers the opportunity to be used anywhere as long as a laptop computer is available. In this study, we evaluated the possibility of MinION nanopore sequencing technology to resolve the mosaic MDR plasmids with the latest R9.4 chemistry.

With the rapid barcoding sequencing kit, complete sequences of twenty-one plasmids harbored by eleven samples could be successfully generated within few days (**Fig. 6**). Although *de novo* assembly of only nanopore long reads by Canu exhibited a relatively low quality of only 97% identity to the reference sequences, the assembled plasmids were found to possess high quality

structural skeletons with correct arrangements of various mobile elements. With Illumina short reads data, accurate complete sequences of plasmids could be obtained by Unicycler which involved three steps: contigs construction with short reads, scaffolding of contigs with long reads and polishing with short reads [14]. Importantly, analysis of the two MDR plasmids in sample RB01 indicated that single long reads could cover the complete plasmid; this finding inferred that the entire plasmid can be sequenced without interruption. In this case, *de novo* assembly was not necessary since several long reads may cover the whole plasmid. The first antibiotic resistance island resolved by MinION nanopore sequencing was reported in 2015 [7]. To the best of our knowledge, this is the first report of complete MDR plasmids sequencing without the need to assemble sheared fragments. It should be noted that, although only a few long reads were found to cover the entire plasmid, they were sufficient to cover all the repetitive sequences in the MDR plasmids. With further improvement in MinION sequencing, a plasmid being sequenced end-to-end as a single molecule will become possible in the near future.

Another advantage of MinION sequencing is that it allows an ongoing sequencing run to halt when sufficient data have been generated, saving time and most importantly the flow cell, which accounts for a significant portion of the cost of MinION sequencing. As a result, the flow cell can be reused several times until most of nanopore pores have lost activity. In this work, we finished the run in eight hours, during which we have generated sufficient data for assembling the complete plasmid sequences. Furthermore, the same flow cell was reused in another run and the data generated were of a quality similar to that of the first run. The standard MinKNOW protocol involves running the flow cells for 48 hours. If one flow cell can accommodate 3 runs, each lasting for 8, 10 and 12 hours respectively, it infers that 36 MDR plasmids samples can be

sequenced in one flow cell using the rapid barcode kit, leading to significant reduction in the cost of producing complete plasmid sequences. Furthermore, real-time hybrid genome assembly approach was reported with npScarf tool, which can overcome over-sequencing issue and shorten analysis timeline [13]. This real-time analysis workflow has the potential to be combined with the plasmids assembly workflow described in this study.

As an extrachromosomal element, plasmids play a dominant role in dissemination of antibiotic resistance genes, virulence genes and other functional genes [15, 16]. Obtaining complete plasmid sequences in a wide range of clinical isolates collected over a prolonged period enable in depth studies of plasmid evolution and adaptation, the underlying mechanisms of transmission of resistance genes, as well as tracking major antibiotic resistant pathogenic bacterial strains [5, 16, 17]. The workflow presented in this work offers for the first time the opportunities to perform these studies in a rapid, cost effective and user-friendly manner.

Methods

Bacterial MDR plasmids extraction

To evaluate the efficiency of MDR plasmids sequencing by MinION platform, we selected twelve MDR plasmids-bearing strains including *E. coli*, *Salmonella spp.*, *V. parahaemolyticus* and *K. pneumoniae* for plasmids extraction (**Table 1**). Overnight cultures (100 mL) were harvested and subjected to plasmid extraction by using the QIAGEN Plasmid Midi Kit. The extracted plasmids were dissolved in ultrapure distilled water and concentrations were measured by Qubit 3.0 Fluorometer with dsDNA BR Assay Kit. The plasmids were stored in -20℃ until library preparation.

MinION library preparation and sequencing

Library preparation was performed using Rapid Barcoding Sequencing kit (SQK-RBK001) according to the standard protocol provided by the manufacturer (Oxford Nanopore). Briefly, 7.5μL plasmid templates were combined with 2.5μL Fragmentation Mix Barcode (one barcode for each sample). The mixtures were incubated at 30℃ for 1 minute and at 75℃ for 1 minute. The barcoded libraries were pooled together with designated ratios in 10μL (**Table 1**). 1μL of RAD (Rapid 1D Adapter) was added to the pooled library and mixed gently. 0.2μL of Blunt/TA Ligase Master Mix was added and incubated for 5 minutes at room temperature. The constructed library was loaded into the Flow Cell R9.4 (FLO-MIN106) on a MinION device and run with SQK-RBK001_plus_Basecaller script of MinKNOW 1.5.12 software. The run was stopped after 8 hours and the flow cell was washed by a Wash Kit (EXP-WSH002) and stored in 4℃ for later use.

Illumina sequencing

To obtain high quality short reads data, pair-end (2×150bp) libraries were prepared by the focused acoustic shearing method with the NEBNext Ultra DNA Library Prep Kit and the Multiplex Oligos Kit for Illumina (NEB). The libraries were quantified by employing qPCR with P5-P7 primers, and pooled together and sequenced on the NextSeq 500 platform according to the manufacturer's protocol (Illumina).

Basecalling, de-mutiplexing, assembly of complete plasmid sequences and data analysis

Although local basecaller script was used during the run, there were still a small amount of reads which skipped being basecalled due to high speed generation of raw data. Albacore basecalling software (v1.0.3) was used to generate fast5 files harboring 1D DNA sequence from fast5 files with only raw data in the tmp folder. Also, read_fast5_basecaller.py script in Albacore was used to de-multiplex the twelve samples from basecalled fast5 files (except the files in fail folder) based on the twelve barcodes in SQK-RBK001. Poretools toolkit was utilized to extract all the DNA sequences from fast5 to fasta format among the twelve samples respectively [18]. Canu assembly tool (v1.3) [14] was used to perform *de novo* assembly of complete plasmid sequences based on nanopore 1D long reads in three consecutive stages including correction, trimming and assembly [14]. The parameter genomeSize was set at 0.5m, 1m, 2m and 4m respectively to optimize the assembly results. High quality complete plasmids were constructed by hybrid *de novo* assembly of Illumina short reads and nanopore long reads data using the Unicycler v0.3 tool [19]. NanoOK was adopted to evaluate the quality of nanopore long reads [20]. BWA MEM was used to align long reads against reference plasmids and visualized by IGV tool [21].

To assess the distribution of resistance genes, mobile elements and replicon genes, the corresponding databases were downloaded [22-24] and BLASTN was performed among the finished plasmids. The result was visualized by in-house R script. Easyfig was utilized to compare the detailed structure of the MDR plasmids [25].

Availability of supporting data

Raw MinION and Illumina sequencing data was deposited in NCBI database with a BioProject PRJNA398365. The twenty-one plasmid sequences of the twelve samples were included as supplementary data in GigaDB repository. The two plasmids in sample RB01 were deposited in NCBI database with accession no. MF353155 and MF353156. The two plasmids assembled by only MinION nanopore long reads in sample RB01 were also attached as supplementary data for reference in GigaDB repository.

Abbreviations

MDR: Multidrug resistance; AMR: antimicrobial resistance; BLAST: The Basic Local Alignment Search Tool; NCBI: National center for biotechnology information; ONT: Oxford nanopore technologies; SMRT: single-molecule, real-time sequencing.

Author contributions

RL conceived and initiated the study. MX, ND and DL performed bacterial isolation and plasmids extraction. RL, XY and MHW performed MinION and Illumina sequencing and data analysis. RL wrote the first draft of the manuscript. EWC revised the manuscript. SC supervised the whole project.

Competing interests

The authors declare no competing financial interests.

Acknowledgements

This research was supported by the Chinese National Key Basic Research and Development (973) Program (2013CB127200) and the Collaborative Research Fund of the Hong Kong Research Grant Council (C7038-15G and C5026-16G).

Reference

1. Holmes AH, Moore LS, Sundsfjord A, Steinbakk M, Regmi S, Karkey A, et al. Understanding the mechanisms and drivers of antimicrobial resistance. Lancet. 2016;387 10014:176-87. doi:10.1016/S0140-6736(15)00473-0.
2. Marston HD, Dixon DM, Knisely JM, Palmore TN and Fauci AS. Antimicrobial Resistance. JAMA. 2016;316 11:1193-204. doi:10.1001/jama.2016.11764.
3. Smillie C, Garcillan-Barcia MP, Francia MV, Rocha EPC and de la Cruz F. Mobility of Plasmids. Microbiol Mol Biol R. 2010;74 3:434-52. doi:Doi 10.1128/Mmbr.00020-10.
4. Beatson SA and Walker MJ. Microbiology. Tracking antibiotic resistance. Science. 2014;345 6203:1454-5. doi:10.1126/science.1260471.
5. Conlan S, Thomas PJ, Deming C, Park M, Lau AF, Dekker JP, et al. Single-molecule sequencing to track plasmid diversity of hospital-associated carbapenemase-producing Enterobacteriaceae. Sci Transl Med. 2014;6 254:254ra126. doi:10.1126/scitranslmed.3009845.
6. Bayliss SC, Hunt VL, Yokoyama M, Thorpe HA and Feil EJ. The use of Oxford Nanopore native barcoding for complete genome assembly. Gigascience. 2017; doi:10.1093/gigascience/gix001.
7. Ashton PM, Nair S, Dallman T, Rubino S, Rabsch W, Mwaigwisya S, et al. MinION nanopore sequencing identifies the position and structure of a bacterial antibiotic resistance island. Nat Biotechnol. 2015;33 3:296-300. doi:10.1038/nbt.3103.
8. Huang Y, Yu X, Xie M, Wang X, Liao K, Xue W, et al. Widespread Dissemination of Carbapenem-Resistant Escherichia coli Sequence Type 167 Strains Harboring blaNDM-5 in Clinical Settings in China. Antimicrobial agents and chemotherapy. 2016;60 7:4364-8. doi:10.1128/AAC.00859-16.
9. Goodwin S, McPherson JD and McCombie WR. Coming of age: ten years of next-generation sequencing technologies. Nat Rev Genet. 2016;17 6:333-51. doi:10.1038/nrg.2016.49.
10. Punina NV, Makridakis NM, Remnev MA and Topunov AF. Whole-genome sequencing targets drug-resistant bacterial infections. Hum Genomics. 2015;9:19. doi:10.1186/s40246-015-0037-z.
11. Ashton PM, Nair S, Dallman T, Rubino S, Rabsch W, Mwaigwisya S, et al. MinION nanopore sequencing identifies the position and structure of a bacterial antibiotic resistance island. Nature Biotechnology. 2015;33 3:296-+. doi:10.1038/nbt.3103.
12. Chin CS, Alexander DH, Marks P, Klammer AA, Drake J, Heiner C, et al. Nonhybrid, finished microbial genome assemblies from long-read SMRT sequencing data. Nat Methods. 2013;10 6:563-9. doi:10.1038/nmeth.2474.
13. Cao MD, Nguyen SH, Ganesamoorthy D, Elliott AG, Cooper MA and Coin LJ. Scaffolding and completing genome assemblies in real-time with nanopore sequencing. Nat Commun. 2017;8:14515. doi:10.1038/ncomms14515.
14. Koren S, Walenz BP, Berlin K, Miller JR, Bergman NH and Phillippy AM. Canu: scalable and accurate long-read assembly via adaptive k-mer weighting and repeat separation. Genome Res. 2017;27 5:722-36. doi:10.1101/gr.215087.116.
15. Johnson TJ and Nolan LK. Pathogenomics of the virulence plasmids of Escherichia coli. Microbiology and molecular biology reviews : MMBR. 2009;73 4:750-74. doi:10.1128/MMBR.00015-09.
16. Conlan S, Park M, Deming C, Thomas PJ, Young AC, Coleman H, et al. Plasmid Dynamics in KPC-Positive Klebsiella pneumoniae during Long-Term Patient Colonization. MBio. 2016;7 3 doi:10.1128/mBio.00742-16.
17. Porse A, Schonning K, Munck C and Sommer MO. Survival and evolution of a large multidrug resistance plasmid in new clinical bacterial hosts. Mol Biol Evol. 2016; doi:10.1093/molbev/msw163.

18. Loman NJ and Quinlan AR. Poretools: a toolkit for analyzing nanopore sequence data. Bioinformatics. 2014;30 23:3399-401. doi:10.1093/bioinformatics/btu555.
19. Wick RR, Judd LM, Gorrie CL and Holt KE. Unicycler: Resolving bacterial genome assemblies from short and long sequencing reads. PLoS Comput Biol. 2017;13 6:e1005595. doi:10.1371/journal.pcbi.1005595.
20. Leggett RM, Heavens D, Caccamo M, Clark MD and Davey RP. NanoOK: multi-reference alignment analysis of nanopore sequencing data, quality and error profiles. Bioinformatics. 2016;32 1:142-4. doi:10.1093/bioinformatics/btv540.
21. Thorvaldsdottir H, Robinson JT and Mesirov JP. Integrative Genomics Viewer (IGV): high-performance genomics data visualization and exploration. Brief Bioinform. 2013;14 2:178-92. doi:10.1093/bib/bbs017.
22. Carattoli A, Zankari E, Garcia-Fernandez A, Larsen MV, Lund O, Villa L, et al. In Silico Detection and Typing of Plasmids using PlasmidFinder and Plasmid Multilocus Sequence Typing. Antimicrobial agents and chemotherapy. 2014;58 7:3895-903. doi:Doi 10.1128/Aac.02412-14.
23. Zankari E, Hasman H, Cosentino S, Vestergaard M, Rasmussen S, Lund O, et al. Identification of acquired antimicrobial resistance genes. The Journal of antimicrobial chemotherapy. 2012;67 11:2640-4. doi:10.1093/jac/dks261.
24. Siguier P, Perochon J, Lestrade L, Mahillon J and Chandler M. ISfinder: the reference centre for bacterial insertion sequences. Nucleic Acids Res. 2006;34 Database issue:D32-6. doi:10.1093/nar/gkj014.
25. Sullivan MJ, Petty NK and Beatson SA. Easyfig: a genome comparison visualizer. Bioinformatics. 2011;27 7:1009-10. doi:DOI 10.1093/bioinformatics/btr039.

Table 1. Technical data of twelve MDR plasmids samples used in the single multiplexed MinION run.

Samples	Marker genes	Species	Plasmid profile[a]	7.5μL(ng)[b]	volume(μl)[c]	quantity(ng)[d]
RB01	*bla*$_{NDM-5}$	*Escherichia coli*	150kb ,100kb	750	0.8	60
RB02	*bla*$_{NDM-5}$	*Escherichia coli*	160kb,135kb,100kb,60kb, 40kb	2010	0.4	160.8
RB03	*bla*$_{NDM-1}$	*Escherichia coli*	330kb,60kb	259.5	1.1	20.76
RB04	*bla*$_{NDM-1}$	*Escherichia coli*	110kb, 130kb, 230kb	937.5	0.7	75
RB05	*bla*$_{CTX-M-15}$	*Escherichia coli*	150kb	484.5	0.8	38.76
RB06	*bla*$_{CTX-M-15}$	*Escherichia coli*	250kb	270	1	21.6
RB07	*bla*$_{CTX-M-15}$	*Vibrio parahaemolyticus*	120kb	654	0.8	52.32
RB08	*bla*$_{CTX-M-3}$, *bla*$_{TEM-1}$	*Salmonella typhimurium*	340kb	885	0.8	70.8
RB09	*bla*$_{KPC-2}$	*Escherichia coli*	70kb	639	0.8	51.12
RB10	*bla*$_{KPC-2}$	*Escherichia coli*	100kb,130kb	346.5	1.1	27.72
RB11	*bla*$_{KPC-2}$	*Klebsiella pneumoniae*	240kb	1125	0.8	90
RB12	*bla*$_{KPC-2}$	*Escherichia coli*	120kb, 100kb	495	0.9	39.6

[a] Plasmid profile was determined by S1 nuclease Pulsed-field gel electrophoresis(PFGE); the sizes of the plasmids were roughly estimated based on S1-PFGE.

[b] The input quantities of plasmid DNA in 7.5μL during library preparation.

[c] The volume of each sample in the pooled library.

[d] The actual quantity of DNA of each sample used in MinION sequencing.

Table 2. Overview of structure and genetic characteristics of twenty one MDR plasmids recovered from eleven samples

Plasmids[a]	Size(bp)	Structural status	No. of resistance genes	No. of insertion sequences	No. of replicon genes
RB01-LZ135-CTX-128976	128976	Circular	8	5	2
RB01-LZ135-NDM-90845	90845	Circular	5	2	1
RB02-JN105-IncF-TET-116277-N	116277	Circular	6	6	2
RB02-JN105-IncN-CTX-139496-N	142307	Circular	9	2	2
RB02-JN105-IncN-NDM6-55342	55342	Circular	3	3	1
RB02-JN105-IncX-NDM5-45823	45823	Circular	1	4	1
RB02-JN105-IncY-CTX-98443	98443	Circular	0	1	1
RB03-WH96T-IncF-OXA-153088	153088	Circular	3	9	4
RB03-WH96T-IncN-NDM1-56215	56215	Circular	2	4	1
RB04-SZ584-1T-IncF-TET-114056	114065	Circular	7	6	2
RB04-SZ584-1T-IncX3-NDM1-56K-NC	55919	Linear	2	4	1
RB04-SZ584-1T-IncY-130821	130821	Circular	0	9	1
RB05-C267-IncA/C-CTX-166467	166467	Circular	10	3	1
RB06-C499-IncA/C-CTX-192739	192739	Circular	11	3	1
RB07-vb0506-IncA/C-CTX-133742	133742	Circular	6	2	1
RB09-IncN-KPC-68571	68571	Circular	7	6	1
RB10-29KPC-IncF-TET-136532	136532	Circular	12	6	3
RB10-29KPC-IncY-KPC-98K-N	95908	Circular	1	2	1

RB11-IncF-IncHI-KPC-238153	238153	Circular	2	10	2
RB12-74T-KPC-IncF-115K-N	115689	Circular	0	6	4
RB12-74T-KPC-IncN-IncX1-KPC-108K-N	107969	Circular	5	4	3

[a] Plasmid names ending with letter N indicated that the plasmids could be assembled by Canu based on MinION nanopore reads, but cannot be assembled using hybrid assembly strategy with Unicycler. Plasmid name ending with NC indicated it was assembled incompletely.

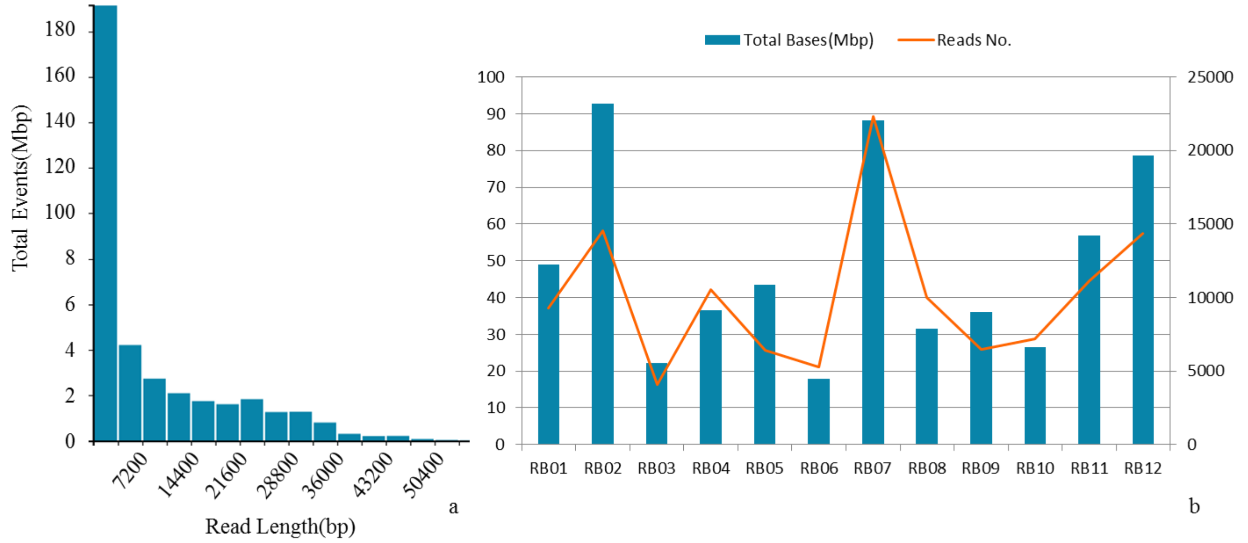

Figure 1. Statistics of an eight-hour MinION nanopore sequencing run using the Rapid Barcoding Sequencing Kit. a, distribution of reads length and data volume generated by the MinION run in eight hours; b, total base length and reads number of the twelve samples after de-multiplexing.

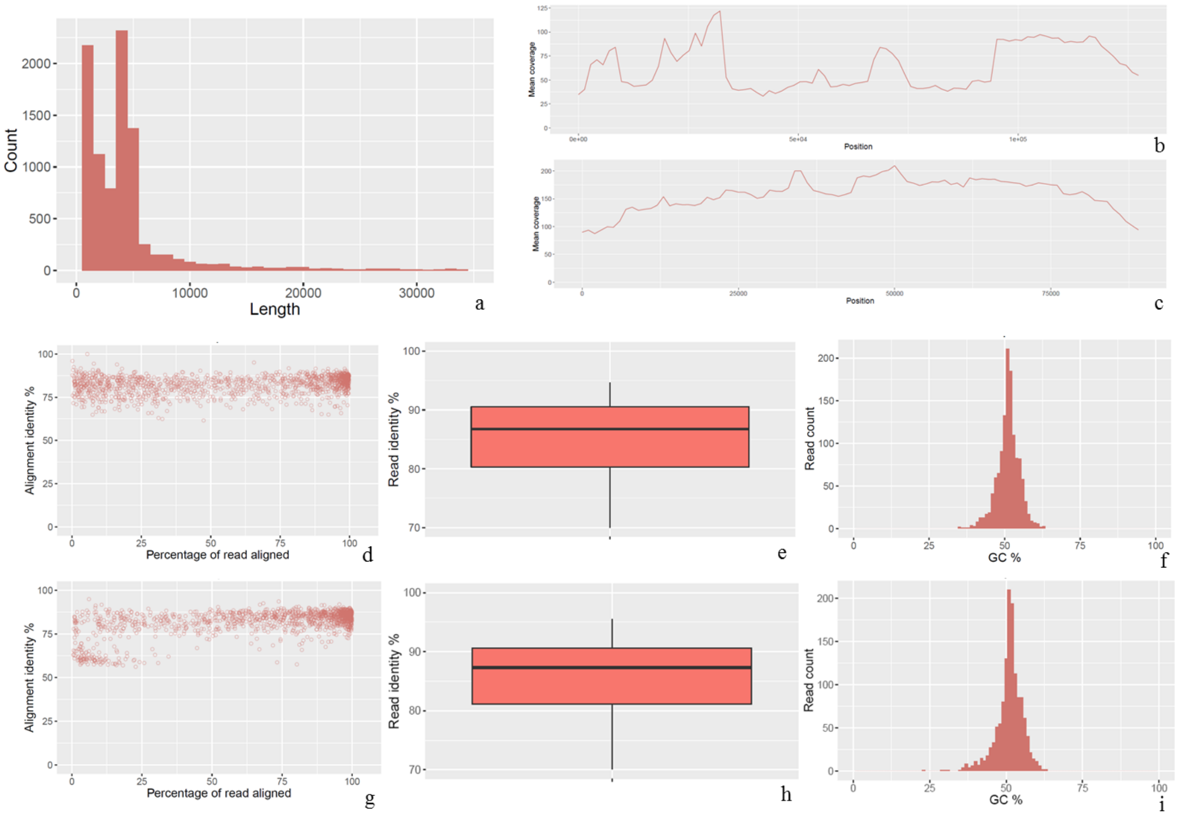

Figure 2. Evaluation of MinION nanopore sequencing long reads quality with nanonet. a, reads counts along with reads length for sample RB01. All the raw reads could be retrieved from the supplemented data. b, nanopore reads coverage with RB01-LZ135-CTX-128976 as reference. c, nanopore reads coverage with RB01-LZ135-NDM-90845 as reference. d, e and f, alignment identity and GC distribution for reads aligned with RB01-LZ135-CTX-128976. g, h and I, alignment identity and GC distribution for reads aligned with RB01-LZ135-NDM-90845.

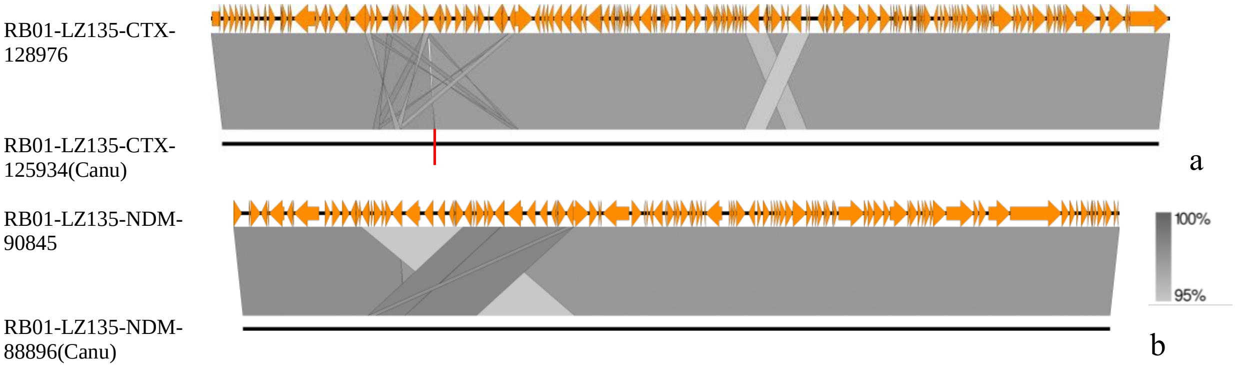

Figure 3. Linear alignment of reference plasmids and corresponding plasmids assembled by Canu based only on MinION nanopore long reads of sample RB01. a, alignment of RB01-LZ135-CTX-128973 and RB01-LZ135-CTX-125934 (Canu). A large deletion that existed in the plasmid RB01-LZ135-CTX-125934 is marked by a red vertical line. b, alignment of RB01-LZ135-NDM-90845 and RB01-LZ135-NDM-88896(Canu). The crossed alignment region indicates that the *bla*$_{NDM-5}$ region was duplicated. The two plasmid sequences assembled by Canu could be retrieved from the supplementary data. The two reference plasmids were deposited in NCBI database.

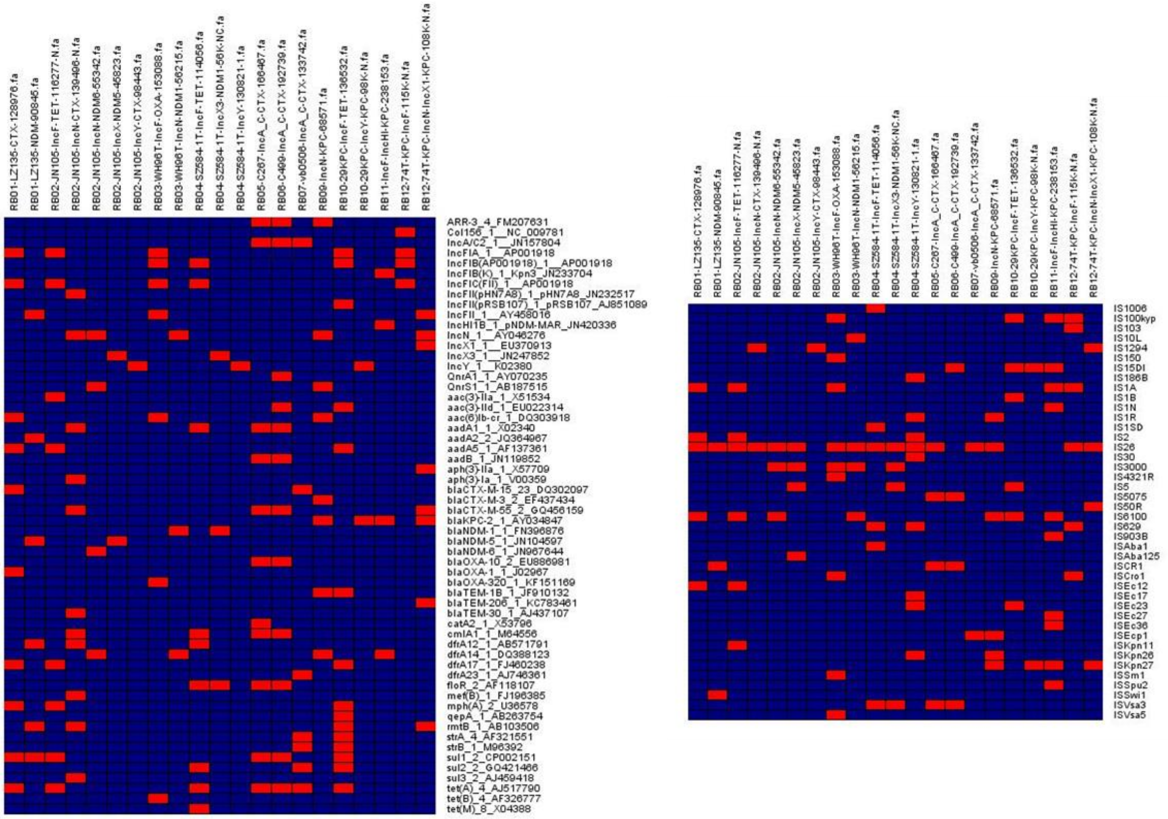

Figure 4. Distribution of resistance genes, replicon genes and insertion sequences among twenty-one plasmids. Red boxes indicate the presence of corresponding genes, and blue boxes indicate absence of the corresponding genes. The twenty-one plasmids sequencing could be retrieved from the supplementary data.

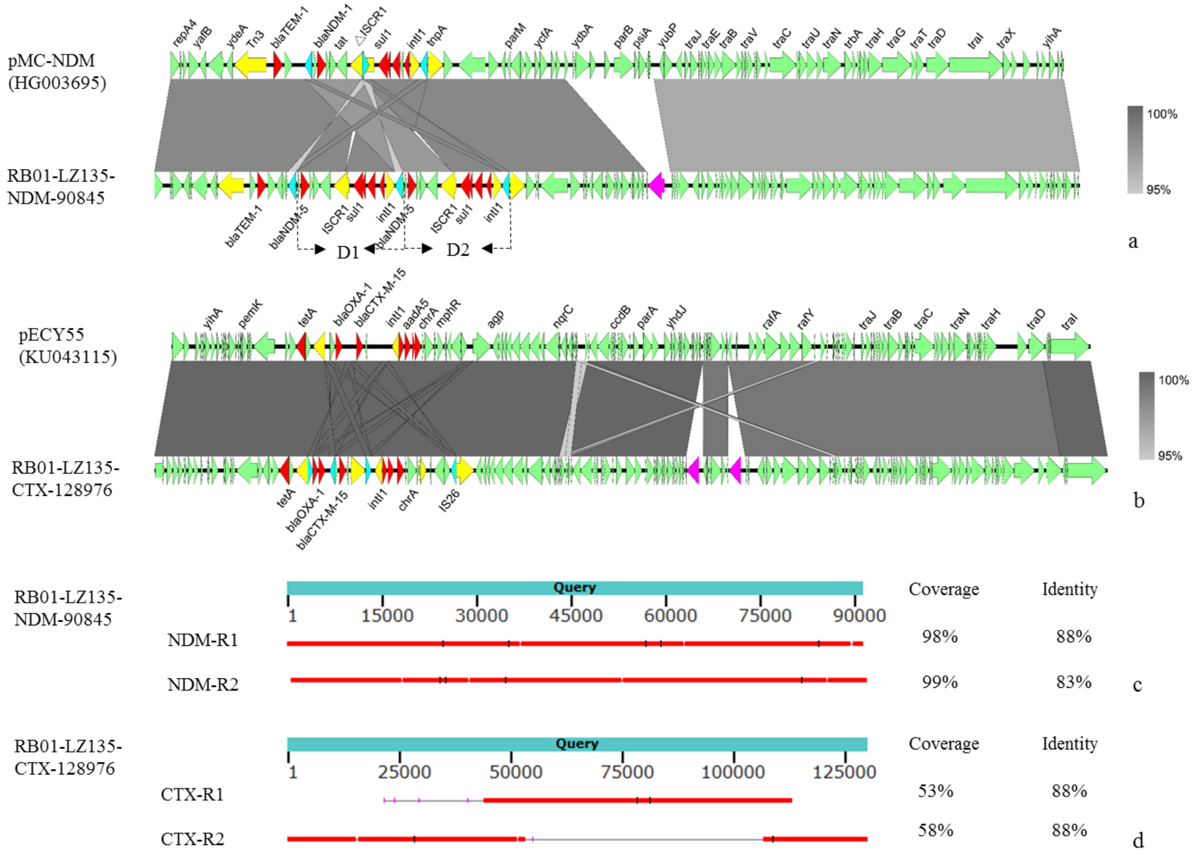

Figure 5. Alignment of plasmids in RB01 with similar structures in NCBI database and MinION nanopore long reads alignment with complete plasmids. a) alignment between pMC-NDM and RB01-LZ135-NDM-90845. The resistance genes were highlighted in red, transposase genes in yellow, IS*26* in cyan, group II intron gene in pink and other CDSs in light green. The sequence contained a large duplication region (ca.10kbp) designated as D1 and D2, each harboring a class 1 integron and a *bla*$_{\text{NDM-1}}$ cluster. b) alignment between pECY55 and RB01-LZ135-CTX-128976. The CDSs were labeled according to the labeling scheme in the Figure. The same group II intron gene were inserted and duplicated in RB01-LZ135-CTX-128976 and compared with pECY55. c) BLASTN of two MinION long reads against RB01-LZ135-NDM-90845. The results indicated that the whole plasmid could be sequenced end-to-

end. d) BLASTN of two MinION long reads against RB01-LZ135-CTX-128976. The results indicated that two MinION long reads could cover the entire plasmid. The four long reads could be retrieved from the supplementary data.

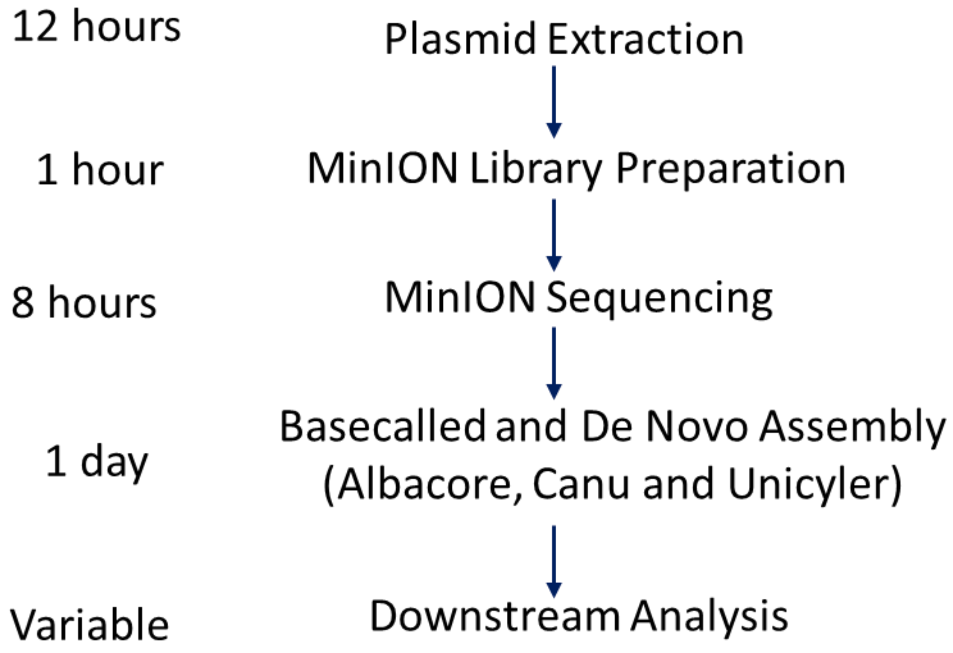

Figure 6. Workflow and time span overview of the MinION nanopore sequencing and assembly process. This workflow was based on the rapid barcoding sequencing kit which could pool twelve samples in a single run. The time for basecalling and *de novo* assembly depended on the computational performance of the computer utilized, and Illumina short reads were needed if Unicycler was used to obtain high quality assembled plasmids.

Click here to access/download
Supplementary Material
supplementary data 1-RB01 plasmids by Canu.fa

Click here to access/download
Supplementary Material
supplementary data 2-twenty one plasmids.fa

Click here to access/download
Supplementary Material
supplementary data 3-four long reads.fa

Click here to access/download
Supplementary Material
RB01-LZ135-CTX-128976-GBK(For review).gbk

Click here to access/download
Supplementary Material
RB01-LZ135-NDM-90845-GBK(For review).gbk
