# Supplementary material for: Efficient generation of complete sequences of MDR-encoding plasmids by rapid assembly of MinION barcoding sequencing data
Source: Gigascience. 2018 Jan 9;7(3):gix132. doi: 10.1093/gigascience/gix132 (PMC5848804; doi:10.1093/gigascience/gix132)
Supplement: GIGA-D-17-00150_Revision_2.pdf [file gix132_giga-d-17-00150_revision_2.pdf]

GigaScience

Efficient generation of complete sequences of MDR-encoding plasmids by rapid assembly of MinION barcoding sequencing data

--Manuscript Draft--

**Manuscript Number:**	GIGA-D-17-00150R2
**Full Title:**	Efficient generation of complete sequences of MDR-encoding plasmids by rapid assembly of MinION barcoding sequencing data
**Article Type:**	Research
**Funding Information:**	973 (2013CB127200) — Dr Sheng CHEN<br>CRF (C7038-15G) — Dr Sheng CHEN<br>CRF (C5026-16G) — Dr Sheng CHEN
**Abstract:**	Background: Multidrug resistance (MDR)-encoding plasmids are considered major molecular vehicles responsible for transmission of antibiotic resistance genes among bacteria of the same or different species. Delineating the complete sequences of such plasmids could provide valuable insight into the evolution and transmission mechanisms underlying bacterial antibiotic resistance development. However, due to the presence of multiple repeats of mobile elements, complete sequencing of MDR plasmids remains technically complicated, expensive and time-consuming.<br><br>Results: Here, we demonstrate a rapid and efficient approach to obtain multiple MDR plasmid sequences through the use of the MinION nanopore sequencing platform, which is incorporated in a portable device. By assembling the long sequencing reads generated by a single MinION run according to a rapid barcoding sequencing protocol, we obtained the complete sequences of twenty plasmids harbored by multiple bacterial strains. Importantly, single long reads covering a plasmid end-to-end were recorded, indicating that de novo assembly may be unnecessary if the single reads exhibit high accuracy.<br><br>Conclusions: This workflow represents a convenient and cost-effective approach for systematic assessment of MDR plasmids responsible for treatment failure of bacterial infections, offering the opportunity to perform detailed molecular epidemiological studies to probe the evolutionary and transmission mechanisms of MDR-encoding elements.
**Corresponding Author:**	Sheng CHEN<br>Hong Kong Polytechnic University<br>Hung Hom, HONG KONG
**Corresponding Author Secondary Information:**	
**Corresponding Author's Institution:**	Hong Kong Polytechnic University
**Corresponding Author's Secondary Institution:**	
**First Author:**	Ruichao Li
**First Author Secondary Information:**	
**Order of Authors:**	Ruichao Li<br>Miaomiao Xie<br>Ning Dong<br>Dachuan Lin<br>Xuemei Yang<br>Marcus Wong<br>Edward Chan

Powered by Editorial Manager® and ProduXion Manager® from Aries Systems Corporation

	Sheng CHEN
**Order of Authors Secondary Information:**	
**Response to Reviewers:**	Responses to reviewers<br>Reviewer reports:<br>Reviewer #1: The response from author is quite clear and adequate. The point of using only Nanopore data to complete genomes of interest in a productive way is made valid through 2 plasmids from sample RB01. However, authors should also include comparison of other Canu assemblies to make clear the pros and cons of using this approach.<br>Another piece of information that would be very helpful to include beside the main manuscript is the detail of using Canu software in this study. Canu relies on a required parameter, namely genomeSize, which is important and the choice of this parameter can vary the results significantly. Interestingly, the authors mentioned they set this parameter at 0.5m, 1m, 2m and 4m for optimization purpose. Regarding this, those who want to apply this method should want to know more about the optimization step that has been conducted, e.g. what exactly is the effect of changing parameter like that and how the final assembly being selected out of all possibilities (without hybrid assemblies or other data as reference).<br><br>Response: Based on the data of RB01, the plasmid sequence assembled by Canu using only Nanopore data showed 97% identity to the reference plasmids assembled by Unicycler using hybrid data of Nanopore and illumine reads. We further conducted similar analysis for other samples as shown in supplementary table 1 showing similar results as RB01. Our data indicated that the hybrid assembly performed by Unicycler was the accurate way to obtain complete genome sequences. For the sequences that could not be assembled by Unicycler, Canu was an option. To summarize, our data suggested that the pros of using Canu assemblies are high efficiency, potential for real-time sequencing and cost effective, while the disadvantage is lower accuracy when compared to hybrid assembly method. With the development of ONT technologies to improve the accuracy of single reads, it is suggested that Canu assemblies should be the best choice in the future. We have included this information in Line 120~128 of Results section.<br><br>Due to the variation of plasmid sizes and the contamination of chromosome in our samples, it is hard to select one fixed size for genomeSize for Canu analysis. We therefore used 0.5m, 1m, 2m and 4m as parameters for genomeSize for optimization purpose. In our study, numbers and sizes of the MDR plasmids in each sample were determined by S1-PFGE method prior to Nanopore sequencing, which greatly help the analysis and pick up right plasmids from the assembled data. Line 265~273.
**Additional Information:**	
**Question**	**Response**
Are you submitting this manuscript to a special series or article collection?	No
**Experimental design and statistics**<br><br>Full details of the experimental design and statistical methods used should be given in the Methods section, as detailed in our Minimum Standards Reporting Checklist. Information essential to interpreting the data presented should be made available in the figure legends.<br><br>Have you included all the information requested in your manuscript?	Yes

Powered by Editorial Manager® and ProduXion Manager® from Aries Systems Corporation

**Resources**<br><br>A description of all resources used, including antibodies, cell lines, animals and software tools, with enough information to allow them to be uniquely identified, should be included in the Methods section. Authors are strongly encouraged to cite Research Resource Identifiers (RRIDs) for antibodies, model organisms and tools, where possible.<br><br>Have you included the information requested as detailed in our Minimum Standards Reporting Checklist?	Yes
**Availability of data and materials**<br><br>All datasets and code on which the conclusions of the paper rely must be either included in your submission or deposited in publicly available repositories (where available and ethically appropriate), referencing such data using a unique identifier in the references and in the “Availability of Data and Materials” section of your manuscript.<br><br>Have you have met the above requirement as detailed in our Minimum Standards Reporting Checklist?	Yes

Manuscript

Click here to download Manuscript Efficient MDR plasmid sequencing R2_SCE_R.docx

Efficient generation of complete sequences of MDR-encoding plasmids by rapid assembly of MinION barcoding sequencing data

Ruichao Li[1,2], Miaomiao Xie[1], Ning Dong[1], Dachuan Lin[1,2], Xuemei Yang[1], Marcus Ho Yin Wong[1], Edward Wai-Chi Chan[2], Sheng Chen[1,2]*

[1] Shenzhen Key Lab for Food Biological Safety Control, Food Safety and Technology Research Center, Hong Kong PolyU Shen Zhen Research Institute, Shenzhen, P. R. China;

[2]The State Key Lab of Chirosciences, Department of Applied Biology and Chemical Technology, The Hong Kong Polytechnic University, Hung Hom, Kowloon, Hong Kong SAR;

*Corresponding author, sheng.chen@polyu.edu.hk, ORCID: 0000-0003-3526-7808.

Running title: Rapid assembly of plasmids by MinION sequencing data

Keywords: Multidrug resistance (MDR) plasmids, *de novo* assembly, nanopore sequencing, long reads

Abstract

Background: Multidrug resistance (MDR)-encoding plasmids are considered major molecular vehicles responsible for transmission of antibiotic resistance genes among bacteria of the same or different species. Delineating the complete sequences of such plasmids could provide valuable insight into the evolution and transmission mechanisms underlying bacterial antibiotic resistance development. However, due to the presence of multiple repeats of mobile elements, complete sequencing of MDR plasmids remains technically complicated, expensive and time-consuming.

Results: Here, we demonstrate a rapid and efficient approach to obtain multiple MDR plasmid sequences through the use of the MinION nanopore sequencing platform, which is incorporated in a portable device. By assembling the long sequencing reads generated by a single MinION run according to a rapid barcoding sequencing protocol, we obtained the complete sequences of twenty plasmids harbored by multiple bacterial strains. Importantly, single long reads covering a plasmid end-to-end were recorded, indicating that *de novo* assembly may be unnecessary if the single reads exhibit high accuracy.

Conclusions: This workflow represents a convenient and cost-effective approach for systematic assessment of MDR plasmids responsible for treatment failure of bacterial infections, offering the opportunity to perform detailed molecular epidemiological studies to probe the evolutionary and transmission mechanisms of MDR-encoding elements.

Introduction

The emergence and increasing prevalence of antimicrobial resistance (AMR) among bacterial pathogens pose increasing public health challenges worldwide by drastically reducing the number of antimicrobials that can be effectively used in treatment of bacterial infections[1, 2].Identification of the key mechanisms responsible for AMR transmission is crucial to combat the threats imposed by AMR. Plasmids, especially MDR-encoding plasmids, are now considered a major vector that facilitates AMR transmission among bacteria via horizontal transfer[3, 4].Delineating the full length of plasmids and genetic structures of other MDR mobile elements are vital for understanding how such elements undergo evolutionary changes and horizontal transmission, and adapt to new host[4]. However, due to the presence of numerous insertion sequences and other repetitive elements in MDR plasmids, it is often difficult and time-consuming to obtain the complete plasmid sequences by next-generation sequencing with short reads and PCR mapping by Sanger sequencing. With the development of long read sequencing technology, tracking plasmid diversity by full assembly of plasmids has become possible[5]. To date, single-molecule, real-time sequencing (SMRT) can generate full-sequence plasmids. However, the huge cost and laborious library preparation procedure of this technology renders it inaccessible for most laboratories.

Recently, another long read sequencing technology based on the use of a portable MinION device has been available from Oxford Nanopore Technologies (ONT). Although the accuracy of reads generated by this technique is generally lower than that of short reads, it exhibits promising capability to generate complete chromosome and plasmid sequences[6, 7]. With the advance of library preparation techniques and data analysis tools, we found that this technology is feasible

for MDR plasmids sequencing. Here, we evaluated the feasibility of decoding the complete sequences of multiple MDR plasmids using MinION Nanopore sequencing technology, through a run with a reusable flow cell within a short time frame. This workflow shall enable laboratories equipped with only basic molecular biology techniques to perform detailed MDR plasmids analysis.

Data description

Raw long sequencing data collected after a MinION run was de-multiplexed by Albacore basecalling software (v1.0.3) to generate fast5 files allocated into twelve samples. The Poretools tool suite was used to extract reads with fasta format and preceded to *de novo* assembly and hybrid assembly with Canu (v1.3) and Unicycler (v0.3). The end result being twenty complete plasmids and one near complete plasmid were efficiently obtained with the data from a single MinION run. The detailed procedures for data analysis were described in Methods.

Results

MinION workflow overview

Twelve MDR plasmids harboring samples were prepared according to the MinION library construction protocols, followed by library sequencing. After eight hours of sequencing run, a total of 287,725 reads ranging from dozens to tens of thousands of bases in length were obtained, covering a total of493Mbp (**Fig. 1a**).It was estimated that the data should be enough for *de novo* assembly; hence the run was stopped manually to save active nanopores for future use. The raw data was subjected to several stages of processing including basecalling, de-multiplexing, fasta sequence extraction and *de novo* assembly as stated in the Methods section. Upon de-multiplexing, a total of 121,584 reads were allocated into the twelve samples, which ranged from 5,273 to 22,319 in reads number and 18 to 93 Mbp in total length (**Fig. 1b**). The reads unsuccessfully basecalled and unclassified reads generated during the de-multiplexing process were excluded from the assembly analysis. By optimizing the parameters of *de novo* assembly tool, we obtained the complete sequences of the MDR plasmids recovered from eleven samples except RB08, which was severely contaminated by chromosomal DNA.

Evaluation of plasmid assembly efficiency

Apart from plasmidRB08, *de novo* assembly was successfully performed on eleven MDR plasmids harboring samples by Canu. High quality assembled sequences were obtained using Unicycler by combining with short reads data. One to five plasmids, which ranged from 46Kb to 238Kb in length, were found in each sample, with a total of twenty complete and one near complete plasmids being obtained from eleven samples (**Table 1**). To evaluate the accuracy of *de novo* assembly of rapid 1D sequencing data generated by the MinION platform, the RB01

sample was selected for comparison between pair-end Illumina sequencing data and nanopore sequencing data. Sequences of two plasmids, RB01-LZ135-CTX-128976 and RB01-LZ135-NDM-90845, were selected for evaluation of the nanopore reads quality (**Fig. 2**). Without size selection during library preparation, the read lengths ranged from 18 to 97,206bp and the N50 was 6,473bp. Based on the alignment of reads to two reference plasmids, the MinION nanopore long reads accuracy was about 87%.

Complete plasmid sequences obtained from *de novo* assembly by Canu based on long reads were compared to the reference plasmids (assembled by Unicycler) by BLASTN. The overall identity of the completed plasmids by Canu was 97%identical to the reference plasmids; the difference mainly due to fabricated deletions in plasmids assembled by Canu, resulting in an overall sequence of 3,043bp and 1,949bpshorter than RB01-LZ135-CTX-128976 and RB01-LZ135-NDM-90845 respectively. No major structural variations were observed between the two different *de novo* assembly methods (**Fig. 3**), indicating that nanopore long reads can be used to accurately resolve the mosaic structures frequently found in plasmids.

Using only Nanopore data to complete plasmids of interest was recommended when no short reads data was available. The hybrid assembly using Unicycleris the accurate way to obtain complete genome sequences. For sequences that could not be resolved by Unicycler, Canu can be an option. Detail comparison results using hybrid assembly by Unicycler and Nanopore data based assembly by Canu was described in **Table S1**. It is suggested that the advantages of assembly using Canu include high efficiency, real-time monitoring and cost effective, while the disadvantage is its lower accuracy when compared with hybrid assembly approach using

Unicycler. With the development of ONT technologies that can significantly improve the accuracy of single reads, assembly using Canu is likely the best choice going forward.

Characterization of MDR plasmids

The number of resistance genes detectable among the twenty complete and one near complete plasmids sequenced in this study ranged from 0 to 12, insertion sequences from 1 to 10 and replicon genes from 1 to 4 (**Table 2, Fig. 4**). This implied that the plasmids tested in this study had complex structures, the complete sequences of which were usually difficult to obtain by short reads sequencing technology due to the presence of numerous repetitive sequences.

To demonstrate the ability of nanopore long reads to resolve the complex structures of MDR plasmids, sample RB01 was investigated in detail. Upon *de novo* assembly, two complete plasmids were obtained and designated as RB01-LZ135-CTX-128976 and RB01-LZ135-NDM-90845 respectively. This sample originated from a clinical carbapenem-resistant *E.coli* strain harboring the *bla*$_{CTX-M-15}$ and*bla*$_{NDM-5}$gene, which was reported previously[8].

In the IncFII type plasmid RB01-LZ135-NDM-90845, which was 90,845bp in length, there was a MDR mosaic region composed of a Tn*3* transposon containing the*bla*$_{TEM-1}$ and *rmtB* genes, andIS*26*-IS*Aba125*-*bla*$_{NDM-5}$-*ble*$_{MBL}$-*traF*-*tat*-IS*CR1*-*sul1*-*qacEdelta1*-*aadA2*-*dfrA12*-*intI1*-IS*26*.Intriguingly, the latter fragment was duplicated in a tandem repeat format (**Fig. 5a**). Online BLASTN of this *bla*$_{NDM-5}$-bearing plasmid in the NCBI database showed that it was highly similar to the plasmid pMC-NDM, which was recovered from a metallo-beta-lactamase-producing *E.coli* strain in Poland (GenBank no. HG003695), with 99% identity at 97% coverage.

The two major differences include existence of tandem repeats and a region replacement (**Fig. 5a**).The *bla*$_{CTX-M-15}$ bearing plasmid RB01-LZ135-CTX-128976 was 128,976bp in length and had a conserved structure similar to that found in plasmid pECY55, which was harbored by a previously reported *E.coli* strain (GenBank no. KU043115), with 99% identity at 97% coverage region. The MDR region harboring *tetA*, *aac(6')-Ib-cr*, *bla*$_{OXA-1}$, *bla*$_{CTX-M-15}$, *dfrA17, aadA5, sul1*, *chrA* and *mph*(A) was shared by these two plasmids, and two group II introns were found inserted in the backbone compared to pECY55 (**Fig.5b**). Detailed analysis of longest reads after BWA MEM alignment showed that two long reads spanned the plasmid RB01-LZ135-NDM-90845 end-to-end, and another two long reads could be aligned to generate plasmid RB01-LZ135-CTX-128976 (**Fig. 5c** and **5d**). This is the first case in which whole plasmid sequence could be generated by only one single read.

Discussion

The advent of next-generation sequencing technologies revolutionizes the study mode in genomic research[9]. Specifically, it has tremendously facilitated molecular epidemiology studies and research on the diversity and evolution of MDR-encoding elements from both clinical and basic research perspectives [4, 10]. Although it is feasible to assess the distribution of resistance genes among single bacterial or metagenomic samples with traditional short reads data, constructing the entire plasmid and chromosome maps that depict the specific location of resistance genes is of vital importance in investigating the evolution features of such genes and tracking the evolution and transmission routes of MDR plasmids [4, 11]. The availability of long read sequencing technologies such as SMRT and MinION nanopore sequencing has shed light on development of efficient approaches to assemble complete genomes with numerous repetitive elements [12, 13]. Owning to the high cost and complex library preparation of SMRT technology, it cannot be commonly utilized in clinical settings and basic molecular laboratories although this technology has been commercially available for more than five years. On the contrary, the recently available portable MinION nanopore sequencing technology offers the opportunity to be used anywhere as long asa laptop computer is available. In this study, we evaluated the possibility of MinION nanopore sequencing technology to resolve the mosaic MDR plasmids with the latest R9.4 chemistry.

With the rapid barcoding sequencing kit, complete sequences of twenty complete (and one near complete) plasmids harbored by eleven samples could be successfully generated within few days (**Fig. 6**). Although *de novo* assembly of only nanopore long reads by Canu exhibiteda relatively low quality of only 97% identity to the reference sequences, the assembled plasmids were found

to possess high quality structural skeletons with correct arrangements of various mobile elements. With Illumina short read data, accurate complete sequences of plasmids could be obtained by Unicycler which involved three steps: contig construction with short reads, scaffolding of contigs with long reads and polishing with short reads[14].Importantly, analysis of the two MDR plasmids in sample RB01indicated that single long reads could cover a complete plasmid; this finding inferred that the entire plasmid can be sequenced without interruption. In this case, *de novo* assembly was not necessary since several long reads may cover the whole plasmid. The first antibiotic resistance island resolved by MinION nanopore sequencing was reported in 2015[7].To the best of our knowledge, this is the first report of complete MDR plasmids sequencing without the need to assemble sheared fragments. It should be noted that, although only a few long reads were found to cover the entire plasmid, they were sufficient to cover all the repetitive sequences in the MDR plasmids. With further improvement in MinION sequencing, a plasmid being sequenced end-to-end as a single molecule will become possible in the near future.

Another advantage of MinION sequencing is that it allows to halt an ongoing sequencing run when sufficient data have been achieved, saving time and most importantly the flow cell, which accounts for a significant portion of the cost of MinION sequencing. As a result, the flow cell can be reused several times until most of nanopores have lost activity. In this work, we finished the run in eight hours, during which the MinION generated sufficient data for assembling the complete plasmid sequences. Furthermore, the same flow cell was reused in another run and the data generated were of similar quality to that of the first run. The standard MinKNOW protocol involves running the flow cells for 48 hours. If one flow cell can accommodate 3 runs, each lasting for 8, 10 and 12 hours respectively, it indicates that 36 MDR plasmids harboring samples

can be sequenced in one flow cell using the rapid barcode kit, leading to significant reduction in the cost of producing complete plasmid sequences. Furthermore, real-time hybrid genome assembly approach was reported with npScarf tool, which can overcome over-sequencing issue and shorten analysis timeline[13]. This real-time analysis workflow has the potential to be combined with the plasmids assembly workflow described in this study.

As an extra chromosomal element, plasmids play a dominant role in dissemination of antibiotic resistance genes, virulence genes and other functional genes [15, 16]. Obtaining complete plasmid sequences in a wide range of clinical isolates collected over a prolonged period enable in depth studies of plasmid evolution and adaptation, the underlying mechanisms of transmission of resistance genes, as well as tracking major antibiotic resistant pathogenic bacterial strains [5, 16, 17]. The workflow presented in this work offers for the first time the opportunity to perform these studies in a rapid, cost effective and user-friendly manner.

Methods

Bacterial MDR plasmids extraction

To evaluate the efficiency of MDR plasmids sequencing by MinION platform, we selected twelve MDR plasmids-bearing strains including *E.coli*, *S.*typhimurium, *V. parahaemolyticus* and *K. pneumoniae* for plasmids extraction (**Table 1**). Overnight cultures (100 mL) were harvested and subjected to plasmid extraction by using the QIAGEN Plasmid Midi Kit. The extracted plasmids were dissolved in ultrapure distilled water and concentrations were measured by Qubit 3.0 Fluorometer with dsDNA BR Assay Kit. The plasmids were stored in -20℃ until library preparation.

MinION library preparation and sequencing

Library preparation was performed using Rapid Barcoding Sequencing kit (SQK-RBK001) according to the standard protocol provided by the manufacturer (Oxford Nanopore). Briefly, 7.5μL plasmid templates were combined with 2.5μL Fragmentation Mix Barcode (one barcode for each sample). The mixtures were incubated at 30℃ for 1 minute and at 75℃ for 1 minute. The barcoded libraries were pooled together with designated ratios in 10μL (**Table 1**). 1μL of RAD (Rapid 1D Adapter) was added to the pooled library and mixed gently. 0.2μL of Blunt/TA Ligase Master Mix was added and incubated for 5 minutes at room temperature. The constructed library was loaded into the Flow Cell R9.4 (FLO-MIN106) on a MinION device and run with SQK-RBK001_plus_Basecaller script of MinKNOW1.5.12 software. The run was stopped after 8 hours and the flow cell was washed by a Wash Kit (EXP-WSH002) and stored in 4℃ for later use.

Illumina sequencing

To obtain high quality short reads data, pair-end (2×150bp) libraries were prepared by the focused acoustic shearing method with the NEBNext Ultra DNA Library Prep Kit and the Multiplex Oligos Kit for Illumina (NEB). The libraries were quantified by employing qPCR with P5-P7 primers, and pooled together and sequenced on the NextSeq 500 platform according to the manufacturer's protocol (Illumina).

Basecalling, de-mutiplexing, assembly of complete plasmid sequences and data analysis

Although local basecaller script was used during the run, there were still a small amount of reads which were not basecalled due to the generation of raw data in a rapid mode. Albacore basecalling software (v1.0.3) was used to generate fast5 files harboring 1D DNA sequence from fast5 files with only raw data in the tmp folder. Also, read_fast5_basecaller.py script in Albacore was used to de-multiplex the twelve samples from basecalled fast5 files (except the files in fail folder) based on the twelve barcodes in SQK-RBK001. Poretools toolkit was utilized to extract all the DNA sequences from fast5 to fasta format among the twelve samples respectively (Poretools, RRID:SCR_015879)[18]. Canu assembly tool (v1.3) (Canu, RRID:SCR_015880)[14]was used to perform *de novo* assembly of complete plasmid sequences based on nanopore 1D long reads in three consecutive stages including correction, trimming and assembly[14]. Due to the possibility of the contamination of bacterial chromosomal DNA in the plasmid samples and the large variation of the size of plasmids, the parameter of genomeSize was set at0.5m, 1m, 2m and 4m respectively to optimize the assembly results to obtain circular plasmid sequences of interest. The sizes and the numbers of plasmid determined by S1-PFGE were used to confirm the assembled results. High quality complete plasmids were constructed by

hybrid *de novo* assembly of Illumina short reads and nanopore long reads data using the Unicycler v0.3 tool[19]. NanoOK was adopted to evaluate the quality of nanopore long reads[20]. BWA MEM was used to align long reads against reference plasmids (BWA , RRID:SCR_010910)[21].

To assess the distribution of resistance genes, mobile elements and replicon genes, the corresponding databases were downloaded[21-23] and BLASTN was performed among the finished plasmids (BLASTN, RRID:SCR_001598). The result was visualized by the tool Genesis[24]. Easyfig was utilized to compare the detailed structures of the MDR plasmids(Easyfig, RRID:SCR_013169)[25].

Availability of supporting data

Raw MinION and Illumina sequencing data are availablein NCBI via the BioProject number PRJNA398365. The twenty complete and one near complete plasmid sequences of the twelve samples were included as supplementary data in the *GigaScience* GigaDB repository[26]. The two plasmids in sample RB01 were deposited in NCBI with the accession numbers MF353155 and MF353156. The two plasmids assembled by only MinION nanopore long reads in sample RB01 are also included as supplementary data for reference in GigaDB [26].

Abbreviations

MDR: Multidrug resistance; AMR: antimicrobial resistance; BLAST: The Basic Local Alignment Search Tool; NCBI: National center for biotechnology information; ONT: Oxford nanopore technologies; SMRT: single-molecule, real-time sequencing.

Author contributions

RL conceived and initiated the study. MX, ND and DL performed bacterial isolation and plasmids extraction. RL, XY and MHW performed MinION and Illumina sequencing and data analysis. RL wrote the first draft of the manuscript. EWC revised the manuscript. SC supervised the whole project and edited the manuscript.

Competing interests

The authors declare no competing financial interests.

Acknowledgements

This research was supported by the Chinese National Key Basic Research and Development (973) Program (2013CB127200) and the Collaborative Research Fund of the Hong Kong Research Grant Council (C7038-15G and C5026-16G).

Reference

1. Holmes AH, Moore LS, Sundsfjord A, Steinbakk M, Regmi S, Karkey A, et al. Understanding the mechanisms and drivers of antimicrobial resistance. Lancet. 2016;387 10014:176-87. doi:10.1016/S0140-6736(15)00473-0.
2. Marston HD, Dixon DM, Knisely JM, Palmore TN and Fauci AS. Antimicrobial Resistance. JAMA. 2016;316 11:1193-204. doi:10.1001/jama.2016.11764.
3. Smillie C, Garcillan-Barcia MP, Francia MV, Rocha EPC and de la Cruz F. Mobility of Plasmids. Microbiol Mol Biol Rev. 2010;74 3:434-52. doi:Doi 10.1128/Mmbr.00020-10.
4. Beatson SA and Walker MJ. Microbiology. Tracking antibiotic resistance. Science. 2014;345 6203:1454-5. doi:10.1126/science.1260471.
5. Conlan S, Thomas PJ, Deming C, Park M, Lau AF, Dekker JP, et al. Single-molecule sequencing to track plasmid diversity of hospital-associated carbapenemase-producing Enterobacteriaceae. Sci Transl Med. 2014;6 254:254ra126. doi:10.1126/scitranslmed.3009845.
6. Bayliss SC, Hunt VL, Yokoyama M, Thorpe HA and Feil EJ. The use of Oxford Nanopore native barcoding for complete genome assembly. Gigascience. 2017; doi:10.1093/gigascience/gix001.
7. Ashton PM, Nair S, Dallman T, Rubino S, Rabsch W, Mwaigwisya S, et al. MinION nanopore sequencing identifies the position and structure of a bacterial antibiotic resistance island. Nat Biotechnol. 2015;33 3:296-300. doi:10.1038/nbt.3103.
8. Huang Y, Yu X, Xie M, Wang X, Liao K, Xue W, et al. Widespread Dissemination of Carbapenem-Resistant Escherichia coli Sequence Type 167 Strains Harboring blaNDM-5 in Clinical Settings in China. Antimicrob Agents Chemother. 2016;60 7:4364-8. doi:10.1128/AAC.00859-16.
9. Goodwin S, McPherson JD and McCombie WR. Coming of age: ten years of next-generation sequencing technologies. Nat Rev Genet. 2016;17 6:333-51. doi:10.1038/nrg.2016.49.
10. Punina NV, Makridakis NM, Remnev MA and Topunov AF. Whole-genome sequencing targets drug-resistant bacterial infections. Hum Genomics. 2015;9:19. doi:10.1186/s40246-015-0037-z.
11. Ashton PM, Nair S, Dallman T, Rubino S, Rabsch W, Mwaigwisya S, et al. MinION nanopore sequencing identifies the position and structure of a bacterial antibiotic resistance island. Nat Biotechnol. 2015;33 3:296-+. doi:10.1038/nbt.3103.
12. Chin CS, Alexander DH, Marks P, Klammer AA, Drake J, Heiner C, et al. Nonhybrid, finished microbial genome assemblies from long-read SMRT sequencing data. Nat Methods. 2013;10 6:563-9. doi:10.1038/nmeth.2474.
13. Cao MD, Nguyen SH, Ganesamoorthy D, Elliott AG, Cooper MA and Coin LJ. Scaffolding and completing genome assemblies in real-time with nanopore sequencing. Nat Commun. 2017;8:14515. doi:10.1038/ncomms14515.
14. Koren S, Walenz BP, Berlin K, Miller JR, Bergman NH and Phillippy AM. Canu: scalable and accurate long-read assembly via adaptive k-mer weighting and repeat separation. Genome Res. 2017;27 5:722-36. doi:10.1101/gr.215087.116.
15. Johnson TJ and Nolan LK. Pathogenomics of the virulence plasmids of Escherichia coli. Microbiol Mol Biol Rev. 2009;73 4:750-74. doi:10.1128/MMBR.00015-09.
16. Conlan S, Park M, Deming C, Thomas PJ, Young AC, Coleman H, et al. Plasmid Dynamics in KPC-Positive Klebsiella pneumoniae during Long-Term Patient Colonization. MBio. 2016;7 3 doi:10.1128/mBio.00742-16.
17. Porse A, Schonning K, Munck C and Sommer MO. Survival and evolution of a large multidrug resistance plasmid in new clinical bacterial hosts. Mol Biol Evol. 2016; doi:10.1093/molbev/msw163.
18. Loman NJ and Quinlan AR. Poretools: a toolkit for analyzing nanopore sequence data. Bioinformatics. 2014;30 23:3399-401. doi:10.1093/bioinformatics/btu555.

19. Wick RR, Judd LM, Gorrie CL and Holt KE. Unicycler: Resolving bacterial genome assemblies from short and long sequencing reads. PLoS Comput Biol. 2017;13 6:e1005595. doi:10.1371/journal.pcbi.1005595.
20. Leggett RM, Heavens D, Caccamo M, Clark MD and Davey RP. NanoOK: multi-reference alignment analysis of nanopore sequencing data, quality and error profiles. Bioinformatics. 2016;32 1:142-4. doi:10.1093/bioinformatics/btv540.
21. Carattoli A, Zankari E, Garcia-Fernandez A, Larsen MV, Lund O, Villa L, et al. In Silico Detection and Typing of Plasmids using PlasmidFinder and Plasmid Multilocus Sequence Typing. Antimicrob Agents Chemother. 2014;58 7:3895-903. doi:Doi 10.1128/Aac.02412-14.
22. Zankari E, Hasman H, Cosentino S, Vestergaard M, Rasmussen S, Lund O, et al. Identification of acquired antimicrobial resistance genes. J Antimicrob Chemother. 2012;67 11:2640-4. doi:10.1093/jac/dks261.
23. Siguier P, Perochon J, Lestrade L, Mahillon J and Chandler M. ISfinder: the reference centre for bacterial insertion sequences. Nucleic Acids Res. 2006;34 Database issue:D32-6. doi:10.1093/nar/gkj014.
24. Sturn A, Quackenbush J and Trajanoski Z. Genesis: cluster analysis of microarray data. Bioinformatics. 2002;18 1:207-8.
25. Sullivan MJ, Petty NK and Beatson SA. Easyfig: a genome comparison visualizer. Bioinformatics. 2011;27 7:1009-10. doi:DOI 10.1093/bioinformatics/btr039.

Table 1. Basicdata of twelve MDR plasmids harboring samples used in the single multiplexed MinION run.

Samples	Marker genes	Species	Plasmid profile[a]	7.5μL(ng)[b]	volume(μl)[c]	quantity(ng)[d]
RB01	*bla*$_{\text{NDM-5}}$	*Escherichia coli*	150kb ,100kb	750	0.8	60
RB02	*bla*$_{\text{NDM-5}}$	*Escherichia coli*	160kb,135kb,100kb,60kb, 40kb	2010	0.4	160.8
RB03	*bla*$_{\text{NDM-1}}$	*Escherichia coli*	330kb,60kb	259.5	1.1	20.76
RB04	*bla*$_{\text{NDM-1}}$	*Escherichia coli*	110kb, 130kb, 230kb	937.5	0.7	75
RB05	*bla*$_{\text{CTX-M-15}}$	*Escherichia coli*	150kb	484.5	0.8	38.76
RB06	*bla*$_{\text{CTX-M-15}}$	*Escherichia coli*	250kb	270	1	21.6
RB07	*bla*$_{\text{CTX-M-15}}$	*Vibrio parahaemolyticus*	120kb	654	0.8	52.32
RB08	*bla*$_{\text{CTX-M-3}}$, *bla*$_{\text{TEM-1}}$	*Salmonella* typhimurium	340kb	885	0.8	70.8
RB09	*bla*$_{\text{KPC-2}}$	*Escherichia coli*	70kb	639	0.8	51.12
RB10	*bla*$_{\text{KPC-2}}$	*Escherichia coli*	100kb,130kb	346.5	1.1	27.72
RB11	*bla*$_{\text{KPC-2}}$	*Klebsiella pneumoniae*	240kb	1125	0.8	90
RB12	*bla*$_{\text{KPC-2}}$	*Escherichia coli*	120kb, 100kb	495	0.9	39.6

[a] Plasmid profile was determined by S1 nuclease Pulsed-field gel electrophoresis(PFGE); the sizes of the plasmids were roughly estimated based on S1-PFGE.
[b] The input quantities of plasmid DNA in 7.5μL during library preparation.
[c]The volume of each sample in the 10μLpooled library.
[d]The actual quantity of DNA of each sample used in MinION sequencing.

Table 2. Overview of structures and genetic characteristics of twenty-one MDR plasmids recovered from eleven samples

Plasmids[a]	Size(bp)	Structural status	No. of resistance genes	No. of insertion sequences	No. of replicon genes
RB01-LZ135-CTX-128976	128976	Circular	8	5	2
RB01-LZ135-NDM-90845	90845	Circular	5	2	1
RB02-JN105-IncF-TET-116277-N	116277	Circular	6	6	2
RB02-JN105-IncN-CTX-139496-N	142307	Circular	9	2	2
RB02-JN105-IncN-NDM6-55342	55342	Circular	3	3	1
RB02-JN105-IncX-NDM5-45823	45823	Circular	1	4	1
RB02-JN105-IncY-CTX-98443	98443	Circular	0	1	1
RB03-WH96T-IncF-OXA-153088	153088	Circular	3	9	4
RB03-WH96T-IncN-NDM1-56215	56215	Circular	2	4	1
RB04-SZ584-1T-IncF-TET-114056	114065	Circular	7	6	2
RB04-SZ584-1T-IncX3-NDM1-56K-NC[b]	55919	Linear	2	4	1
RB04-SZ584-1T-IncY-130821	130821	Circular	0	9	1
RB05-C267-IncA/C-CTX-166467	166467	Circular	10	3	1
RB06-C499-IncA/C-CTX-192739	192739	Circular	11	3	1
RB07-vb0506-IncA/C-CTX-133742	133742	Circular	6	2	1
RB09-IncN-KPC-68571	68571	Circular	7	6	1
RB10-29KPC-IncF-TET-136532	136532	Circular	12	6	3
RB10-29KPC-IncY-KPC-98K-N	95908	Circular	1	2	1
RB11-IncF-IncHI-KPC-238153	238153	Circular	2	10	2
RB12-74T-KPC-IncF-115K-N	115689	Circular	0	6	4
RB12-74T-KPC-IncN-IncX1-KPC-108K-N	107969	Circular	5	4	3

[a]Plasmid names ending with a letter N indicated that the plasmids could be assembled by Canu based on MinION nanopore reads, but cannot be assembled using hybrid assembly strategy with Unicycler. Plasmid name ending with NC indicated it was assembled incompletely due to low coverage of reads resulted from low copy number of large plasmids.

[b]Although this plasmid was not fully completed, it was still used to do further analysis together with other plasmids.

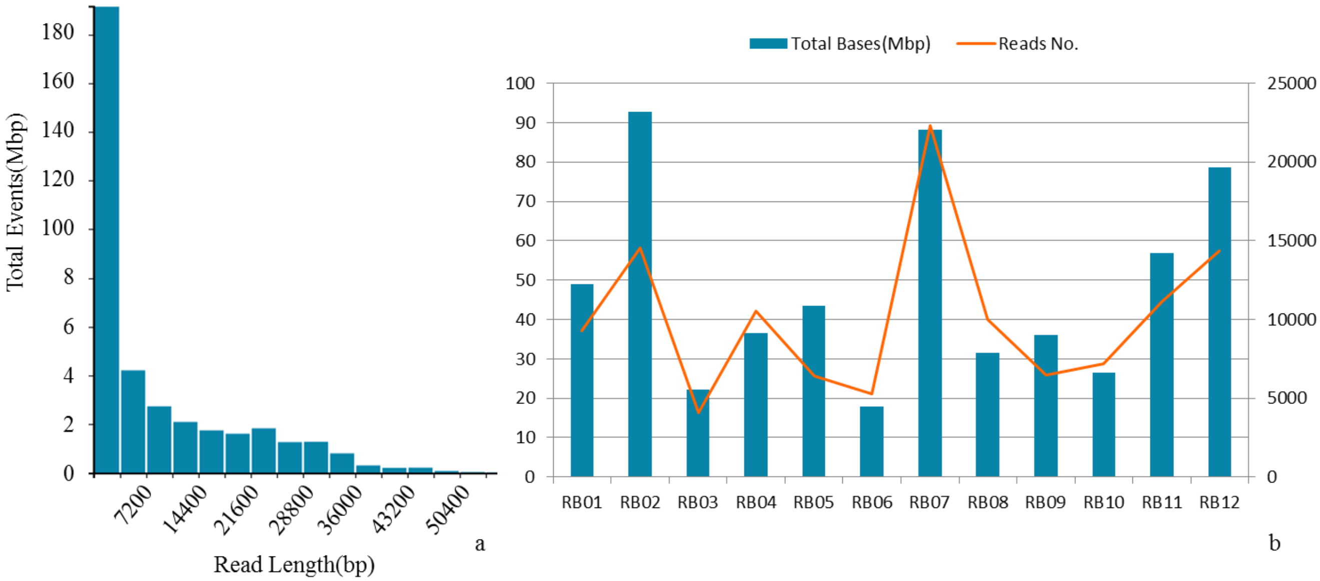

Figure 1.Statistics of an eight-hour MinION nanopore sequencing run using the Rapid Barcoding Sequencing Kit. a)distribution of reads length and data volume generated by the MinION run in eight hours; b) total base length and reads number of the twelve samples after de-multiplexing.

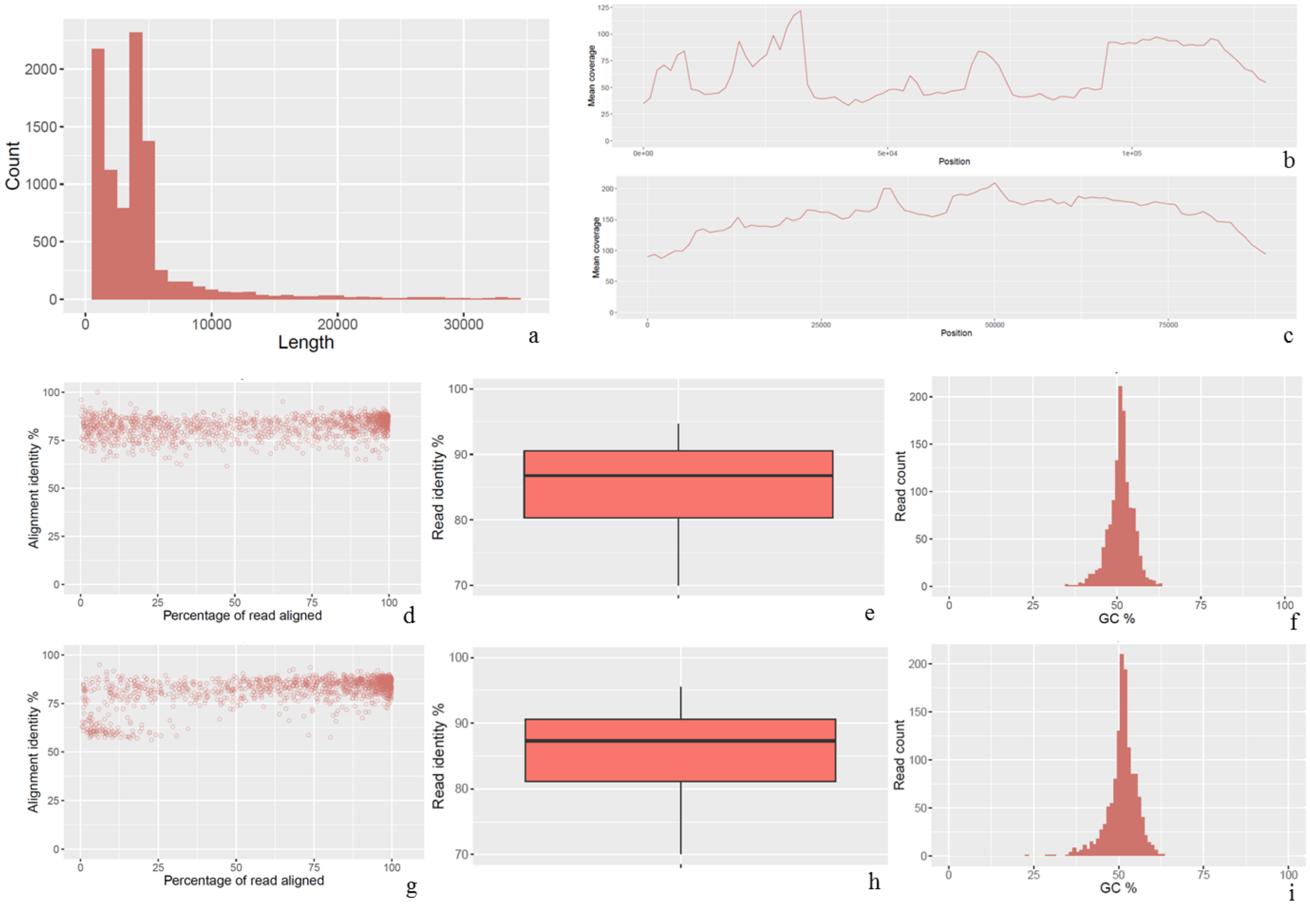

Figure 2.Evaluation of MinION nanopore sequencing long reads quality with nanonet. a) reads counts along with reads length for sample RB01. All the raw reads could be retrieved from the supplementary data. b) nanopore reads coverage with RB01-LZ135-CTX-128976 as reference. c), nanopore reads coverage with RB01-LZ135-NDM-90845 as reference. d), e) and f) alignment identity and GC distribution for reads aligned with RB01-LZ135-CTX-128976. g), h) and I) alignment identity and GC distribution for reads aligned with RB01-LZ135-NDM-90845.

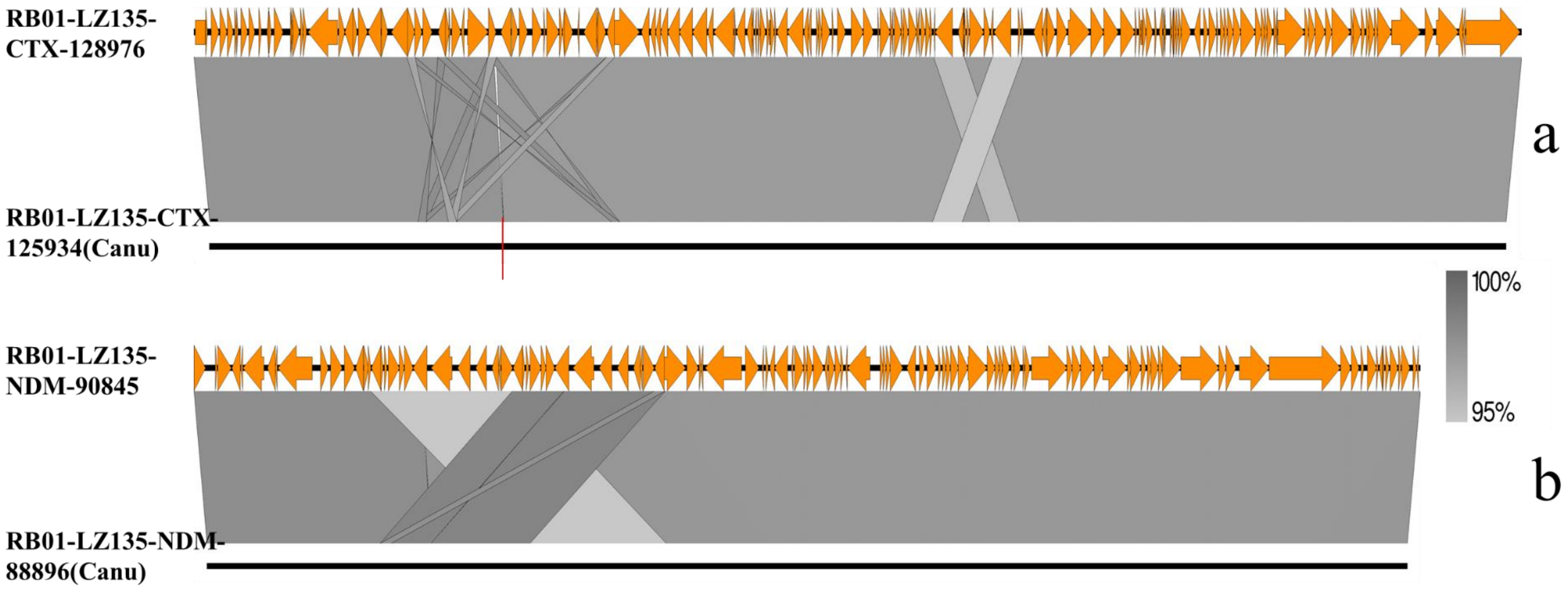

Figure 3. Linear alignment of reference plasmids and corresponding plasmids assembled by Canu based only on MinION nanopore long reads of sample RB01. a)alignment of RB01-LZ135-CTX-128973 and RB01-LZ135-CTX-125934 (Canu). A large deletion that existed in the plasmid RB01-LZ135-CTX-125934 is marked by a red vertical line. b) alignment of RB01-LZ135-NDM-90845 and RB01-LZ135-NDM-88896 (Canu). The crossed alignment region indicates that the $\textit{bla}_{\text{NDM-5}}$region was duplicated. The two plasmid sequences assembled by Canu could be retrieved from the supplementary data. The two reference plasmids were deposited in NCBI database.

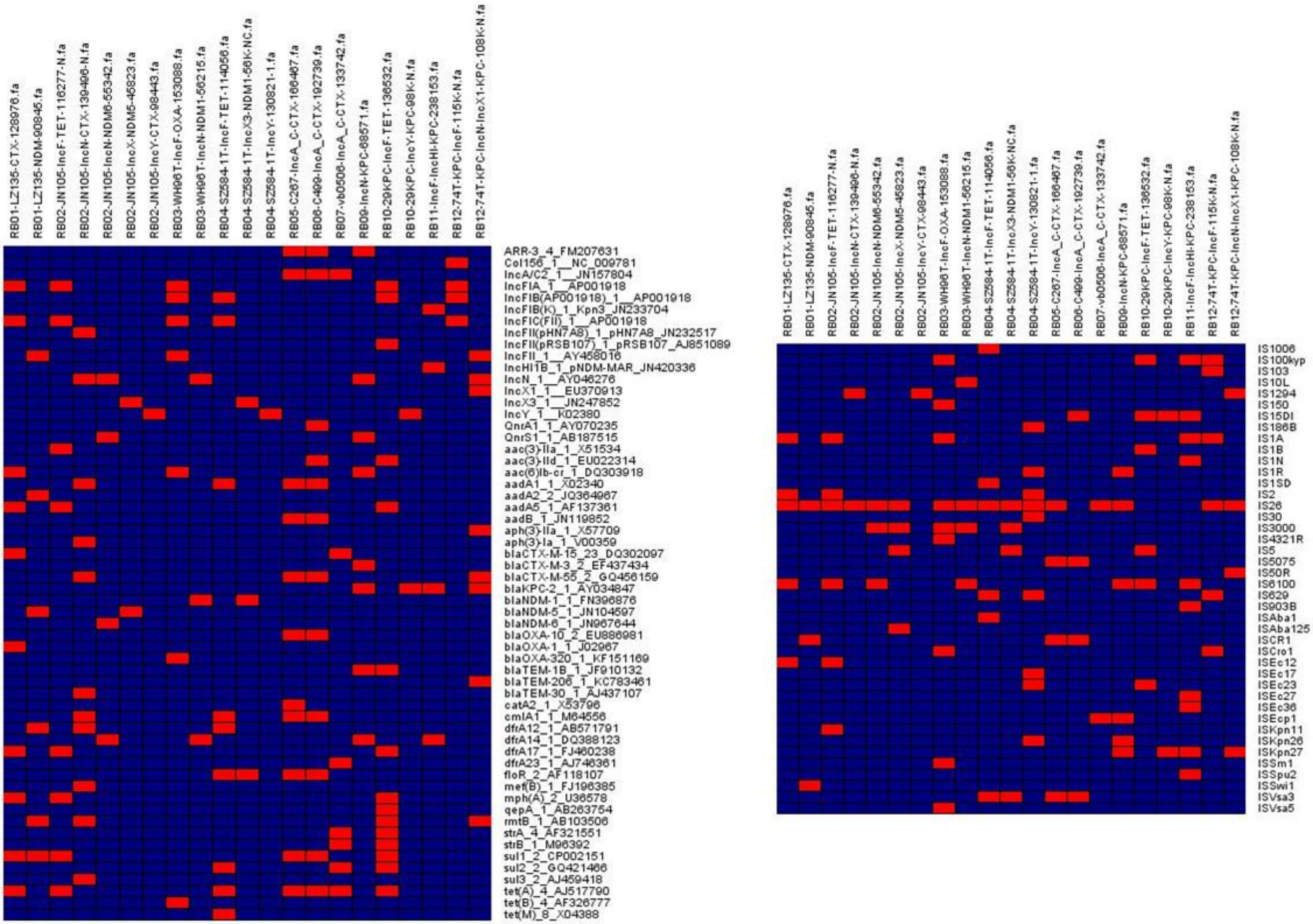
RB01-LZ135-CTX-128976.fa
RB01-LZ135-NDM-90845.fa
RB02-JN105-IncF-TET-116277-N.fa
RB02-JN105-IncN-CTX-139496-N.fa
RB02-JN105-IncN-NDM6-55342.fa
RB02-JN105-IncX-NDM5-45823.fa
RB02-JN105-IncY-CTX-98443.fa
RB03-WH96T-IncF-OXA-153088.fa
RB03-WH96T-IncN-NDM1-56215.fa
RB04-SZ584-1T-IncF-TET-114056.fa
RB04-SZ584-1T-IncX3-NDM1-56K-NC.fa
RB04-SZ584-1T-IncY-130821-1.fa
RB05-C267-IncA_C-CTX-166467.fa
RB06-C499-IncA_C-CTX-192739.fa
RB07-vb0506-IncA_C-CTX-133742.fa
RB09-IncN-KPC-68571.fa
RB10-29KPC-IncF-TET-136532.fa
RB10-29KPC-IncY-KPC-98K-N.fa
RB11-IncF-IncHI-KPC-238153.fa
RB12-74T-KPC-IncF-115K-N.fa
RB12-74T-KPC-IncN-IncX1-KPC-108K-N.fa
ARR-3_4_FM207631
Col156_1__NC_009781
IncA/C2_1__JN157804
IncFIA_1__AP001918
IncFIB(AP001918)_1__AP001918
IncFIB(K)_1_Kpn3_JN233704
IncFIC(FII)_1__AP001918
IncFII(pHN7A8)_1_pHN7A8_JN232517
IncFII(pRSB107)_1_pRSB107_AJ851089
IncFII_1__AY458016
IncHI1B_1_pNDM-MAR_JN420336
IncN_1__AY046276
IncX1_1__EU370913
IncX3_1__JN247852
IncY_1__K02380
QnrA1_1_AY070235
QnrS1_1_AB187515
aac(3)-IIa_1_X51534
aac(3)-IId_1_EU022314
aac(6')Ib-cr_1_DQ303918
aadA1_1_X02340
aadA2_2_JQ364967
aadA5_1_AF137361
aadB_1_JN119852
aph(3')-IIa_1_X57709
aph(3')-Ia_1_V00359
blaCTX-M-15_23_DQ302097
blaCTX-M-3_2_EF437434
blaCTX-M-55_2_GQ456159
blaKPC-2_1_AY034847
blaNDM-1_1_FN396876
blaNDM-5_1_JN104597
blaNDM-6_1_JN967644
blaOXA-10_2_EU886981
blaOXA-1_1_J02967
blaOXA-320_1_KF151169
blaTEM-1B_1_JF910132
blaTEM-206_1_KC783461
blaTEM-30_1_AJ437107
catA2_1_X53796
cmlA1_1_M64556
dfrA12_1_AB571791
dfrA14_1_DQ388123
dfrA17_1_FJ460238
dfrA23_1_AJ746361
floR_2_AF118107
mef(B)_1_FJ196385
mph(A)_2_U36578
qepA_1_AB263754
rmtB_1_AB103506
strA_4_AF321551
strB_1_M96392
sul1_2_CP002151
sul2_2_GQ421466
sul3_2_AJ459418
tet(A)_4_AJ517790
tet(B)_4_AF326777
tet(M)_8_X04388
RB01-LZ135-CTX-128976.fa
RB01-LZ135-NDM-90845.fa
RB02-JN105-IncF-TET-116277-N.fa
RB02-JN105-IncN-CTX-139496-N.fa
RB02-JN105-IncN-NDM6-55342.fa
RB02-JN105-IncX-NDM5-45823.fa
RB02-JN105-IncY-CTX-98443.fa
RB03-WH96T-IncF-OXA-153088.fa
RB03-WH96T-IncN-NDM1-56215.fa
RB04-SZ584-1T-IncF-TET-114056.fa
RB04-SZ584-1T-IncX3-NDM1-56K-NC.fa
RB04-SZ584-1T-IncY-130821-1.fa
RB05-C267-IncA_C-CTX-166467.fa
RB06-C499-IncA_C-CTX-192739.fa
RB07-vb0506-IncA_C-CTX-133742.fa
RB09-IncN-KPC-68571.fa
RB10-29KPC-IncF-TET-136532.fa
RB10-29KPC-IncY-KPC-98K-N.fa
RB11-IncF-IncHI-KPC-238153.fa
RB12-74T-KPC-IncF-115K-N.fa
RB12-74T-KPC-IncN-IncX1-KPC-108K-N.fa
IS1006
IS100kyp
IS103
IS10L
IS1294
IS150
IS15DI
IS186B
IS1A
IS1B
IS1N
IS1R
IS1SD
IS2
IS26
IS30
IS3000
IS4321R
IS5
IS5075
IS50R
IS6100
IS629
IS903B
ISAba1
ISAba125
ISCR1
ISCro1
ISEc12
ISEc17
ISEc23
ISEc27
ISEc36
ISEcp1
ISKpn11
ISKpn26
ISKpn27
ISSm1
ISSpu2
ISSwi1
ISVsa3
ISVsa5

Figure 4. Distribution of resistance genes, replicon genes and insertion sequences among twenty-one plasmids. Red boxes indicate the presence of corresponding genes, and blue boxes indicate absence of the corresponding genes. The twenty one plasmids sequences can be retrieved from the supplementary data. It should be noted that one plasmid RB04-SZ584-1T-IncX3-NDM1-56K-NC was not fully completed.

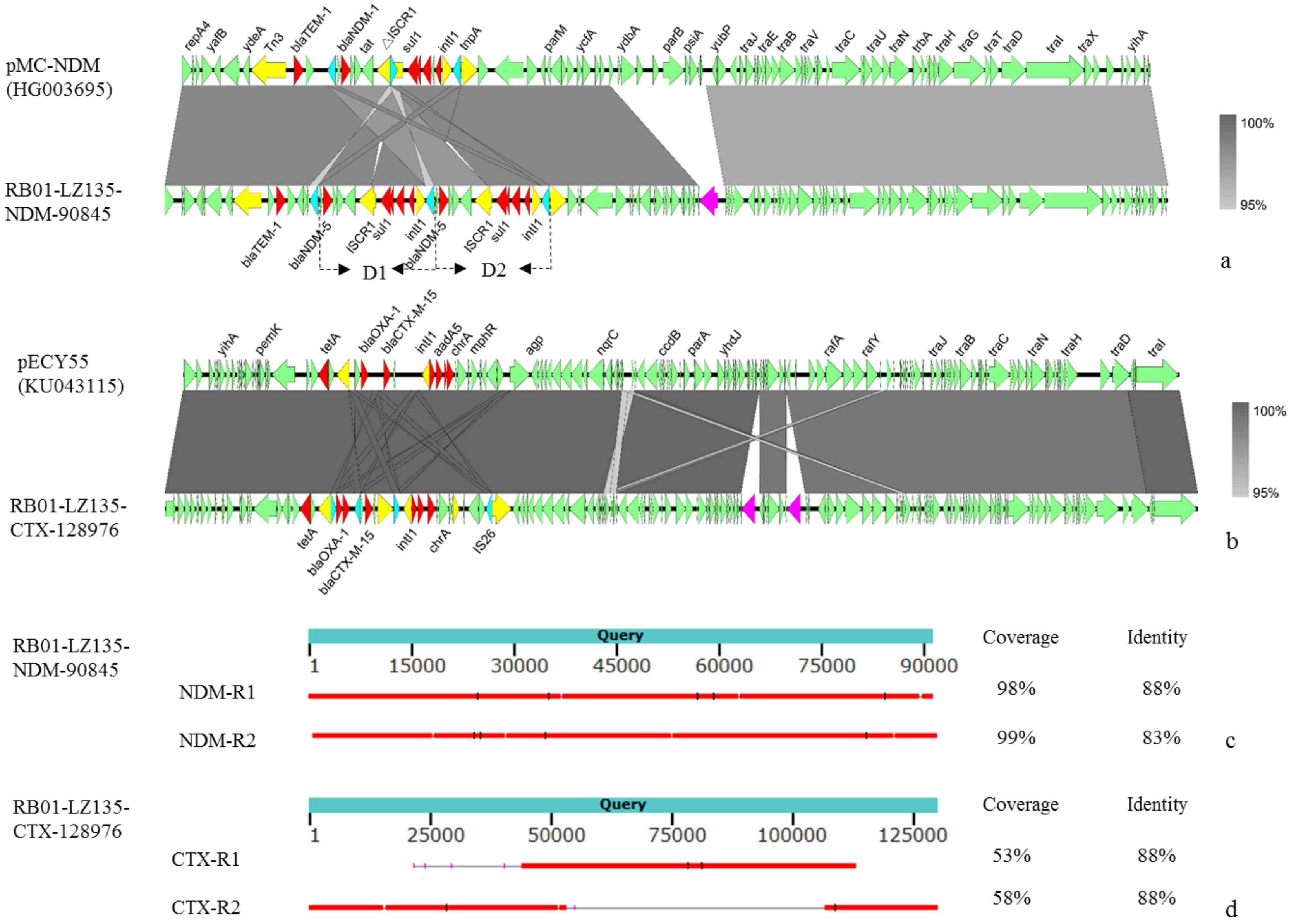

pMC-NDM
(HG003695)
RB01-LZ135-
NDM-90845
repA4
yafB
ydeA
Tn3
blaTEM-1
blaNDM-1
tat
△ISCR1
sul1
intI1
tnpA
parM
ycfA
ydbA
parB
psiA
yubP
traJ
traE
traB
traV
traC
traU
traN
trbA
traH
traG
traT
traD
traI
traX
yihA
blaTEM-1
blaNDM-5
ISCR1
sul1
intI1
blaNDM-5
ISCR1
sul1
intI1
D1
D2
100%
95%
a
pECY55
(KU043115)
RB01-LZ135-
CTX-128976
yihA
pemK
tetA
blaOXA-1
blaCTX-M-15
intI1
aadA5
chrA
mphR
agp
nqrC
ccdB
parA
yhdJ
rafA
rafY
traJ
traB
traC
traN
traH
traD
traI
tetA
blaOXA-1
blaCTX-M-15
intI1
chrA
IS26
100%
95%
b
RB01-LZ135-
NDM-90845
Query
1
15000
30000
45000
60000
75000
90000
Coverage
Identity
NDM-R1
98%
88%
NDM-R2
99%
83%
c
RB01-LZ135-
CTX-128976
Query
1
25000
50000
75000
100000
125000
Coverage
Identity
CTX-R1
53%
88%
CTX-R2
58%
88%
d

Figure 5. Alignment of plasmids in RB01 with similar structures in NCBI database and MinION nanopore long reads alignment with complete plasmids. a) alignment between pMC-NDM and RB01-LZ135-NDM-90845. The resistance genes were highlighted in red, transposase genes in yellow, IS*26* in cyan, group II intron gene in pink and other CDSs in light green. The sequence contained a large duplication region (ca.10kbp) designated as D1 and D2, each harboring a class 1 integron and a*bla*$_{NDM-1}$ cluster. b) alignment between pECY55 and RB01-LZ135-CTX-128976. The CDSs were labeled according to the labeling scheme in the Figure 5a. The same group II intron gene were inserted and duplicated in RB01-LZ135-CTX-128976 compared with pECY55. c) BLASTN of two MinION long reads against RB01-LZ135-NDM-90845. The results indicated that the whole plasmid could be sequenced end-to-end. d) BLASTN of two MinION long reads against RB01-LZ135-CTX-128976. The results indicated that two MinION long reads could cover the entire plasmid. The four long reads could be retrieved from the supplementary data.

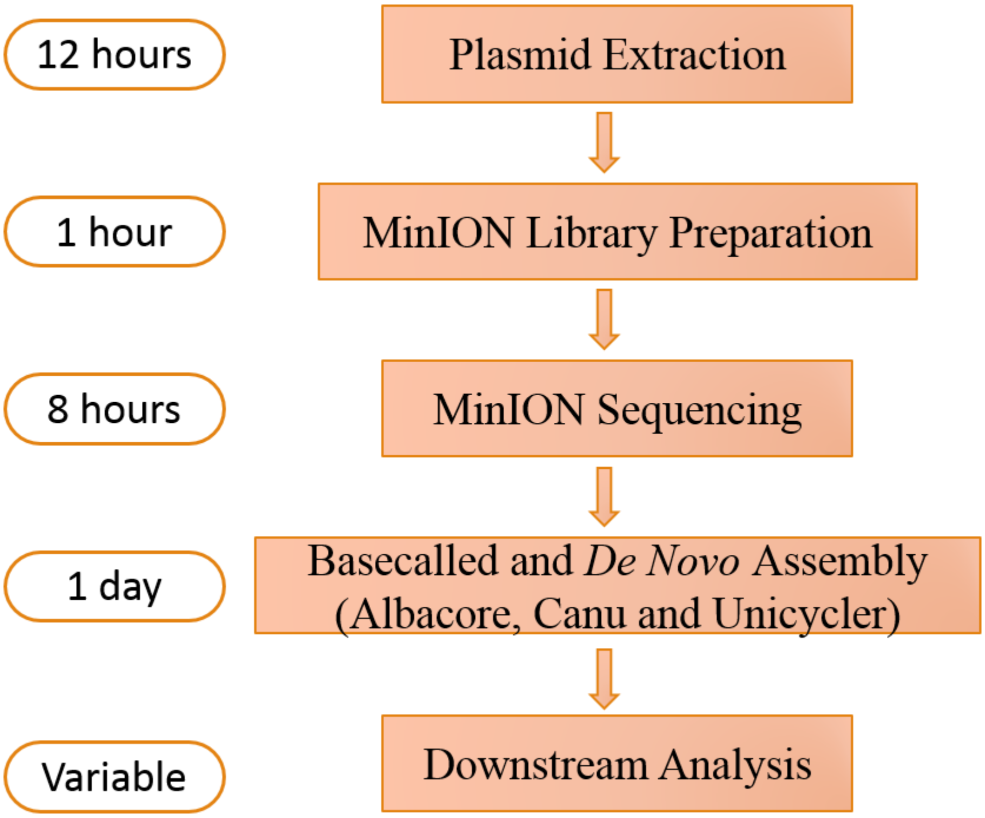

Figure 6. Workflow and time span overview of the MinION nanopore sequencing and assembly process. This workflow was based on the rapid barcoding sequencing kit which could pool twelve samples in a single run. The time for basecalling and *de novo* assembly depended on the computational performance of the computer utilized, and Illumina short reads were needed if Unicycler was used to obtain high quality assembled plasmids.

Supplementary Table 1

Click here to access/download
Supplementary Material
Supplementary Table 1-R2.docx

Click here to access/download
Supplementary Material
supplementary data 1-RB01 plasmids by Canu.fa

Click here to access/download
Supplementary Material
supplementary data 2-twenty one plasmids.fa

Click here to access/download
Supplementary Material
supplementary data 3-four long reads.fa

Click here to access/download
Supplementary Material
RB01-LZ135-CTX-128976-GBK(For review).gbk

Click here to access/download
Supplementary Material
RB01-LZ135-NDM-90845-GBK(For review).gbk
