# Supplementary material for: Efficient generation of complete sequences of MDR-encoding plasmids by rapid assembly of MinION barcoding sequencing data
Source: Gigascience. 2018 Jan 9;7(3):gix132. doi: 10.1093/gigascience/gix132 (PMC5848804; doi:10.1093/gigascience/gix132)
Supplement: Supplemental material [file gix132_supp.zip › Supplementary Table 1-R2.docx]

**Efficient generation of complete sequences of MDR-encoding plasmids by rapid assembly of MinION barcoding sequencing data**

Ruichao Li^1,2^, Miaomiao Xie^1^, Ning Dong^1^, Dachuan Lin^1,2^, Xuemei Yang^1^, Marcus Ho Yin Wong^1^, Edward Wai-Chi Chan^2^, Sheng Chen^1,2^*

^1^ Shenzhen Key Lab for Food Biological Safety Control, Food Safety and Technology Research Center, Hong Kong PolyU Shen Zhen Research Institute, Shenzhen, P. R. China;

^2^ The State Key Lab of Chirosciences, Department of Applied Biology and Chemical Technology, The Hong Kong Polytechnic University, Hung Hom, Kowloon, Hong Kong SAR;

**Supplementary Table 1.** Comparison between plasmid sequences of first four samples assembled by Unicycler using both Illumina and Nanopore data and by Canu using only Nanopore data.

| **Plasmids^a^** | **Unicycler assembly (bp) ^b^** | **Structural status** | **Canu assembly (bp)** | **Identity ^c^** | **No. of replicon genes** |
| --- | --- | --- | --- | --- | --- |
| RB01-LZ135-CTX-128976 | 128976 | Circular | 125934 | 97% | 2 |
| RB01-LZ135-NDM-90845 | 90845 | Circular | 88896 | 97% | 1 |
| RB02-JN105-IncF-TET-116277-N | NA | Circular | 116277 | NA | 2 |
| RB02-JN105-IncN-CTX-139496-N | NA | Circular | 142307 | NA | 2 |
| RB02-JN105-IncN-NDM6-55342 | 55342 | Circular | 58756 | 86% | 1 |
| RB02-JN105-IncX-NDM5-45823 | 45823 | Circular | 44855 | 98% | 1 |
| RB02-JN105-IncY-CTX-98443 | 98443 | Circular | 96693 | 98% | 1 |
| RB03-WH96T-IncF-OXA-153088 | 153088 | Circular | 148950 | 97% | 4 |
| RB03-WH96T-IncN-NDM1-56215 | 56215 | Circular | 55062 | 98% | 1 |
| RB04-SZM584-1T-IncF-TET-114056 | 114065 | Circular | 111703 | 98% | 2 |
| RB04-SZM584-1T-IncX3-NDM1-56K-NC | NA | Linear | 55919 | NA | 1 |
| RB04-SZM584-1T-IncY-130821 | 130821 | Circular | 131934 | 96% | 1 |

^a^ Plasmid with a letter N ending at its name indicated that the plasmid could be assembled by Canu based on MinION nanopore reads, but failed to be assembled using hybrid assembly strategy with Unicycler. Plasmid with a letter NC ending at its name indicated it was assembled incompletely due to low coverage of reads resulted from low copy number of large plasmids.

^b^ NA indicates that the assembled results based on Unicycler are not available due to low data volume of Illumina sequencing raw data.

^c^ Identity indicates the percentage of sequence identity between palsmids assembled by Canu using that assembled by Unicycler as references. The low identity value of plasmid RB02-JN105-IncN-NDM6-55342 implied that the MDR region might not be assembled well either by Unicycler or Canu method, which needs further sequencing data.
