# Supplementary material for: Efficient generation of complete sequences of MDR-encoding plasmids by rapid assembly of MinION barcoding sequencing data
Source: Gigascience. 2018 Jan 9;7(3):gix132. doi: 10.1093/gigascience/gix132 (PMC5848804; doi:10.1093/gigascience/gix132)
Supplement: Response_to_Reviewer_Comments_Original_Submission.pdf [file gix132_response_to_reviewer_comments_original_submission.pdf]

Author's Response To Reviewer Comments

Responses to reviewers

Reviewer reports:

Reviewer #1:
Q1. This study is about a method to obtain completed MDR-encoding plasmids while minimizing the turnaround time. The idea is to use MinION barcode sequencing on extracted plasmids and then use computational tools to assembly them. Although there are several studies conceived with similar approach (Sion C. Bayliss et al 2017, Ryan R. Wick et al 2017), they're focusing on completing the whole bacteria genomes rather than MDR-plasmids of interest alone. The question is how this study serves its purpose more efficient than aforementioned methods, when the turn-around time and cost are about similar?

Response: Our study describes an approach to obtain completed MDR-encoding plasmids in a cost-effective and and a much more efficient manner when compared with the previous methods (Illumina plus PCR mapping, PacBio, etc.). Although there have been a few studies on producing Oxford Nanopore long read data, the research focus, turn-around time and cost in the previous studies (Sion C. Bayliss et al 2017, Ryan R. Wick et al 2017) are quite different from ours.

Silon C. Bayliss and colleagues (Sion C. Bayliss et al 2017) reported the use of Oxford Nanopore native barcoding method for complete genome assembly. However, the method they used was native barcoding method, which takes more time and needs various costly consumables. Although they sequenced a multiplexed library of 12 samples, only one successfully assembled sample, namely Staphylococcus aureus MHO_00,1 was presented. Most importantly, the MinION 2D technology they used in the study has been replaced by 1D technology by Oxford Nanopore technologies. To some extent, the workflow in their study was 'old-fashioned'. Compared with the pilot study conducted by Silon C. Bayliss, our study presents a much more efficient approach to assemble MDR-encoding plasmids with less lab equipments and consumables. Also, the flow cell was found to be reusable for at least three multiplexed libraries, which result in dramatic decrease of cost incurred. Users will find these information highly practical.

Ryan R. Wick and colleagues (Ryan R. Wick et al 2017) developed a very useful tool Unicycler, which is a new tool for assembling bacterial genomes from a combination of short and long reads. In their study, they focus on evaluation of the tool performance based on simulated and real data compared with other assembly tools. In our study, we utilize this useful Unicycler tool to perform hybrid assembly and generate highly accurate data in specially designed workflow for finishing MDR-encoding plasmids, which is a read-to-use approach (from samples to complete sequences) highly suitable for conventional molecular labs. The research purposes are different between ours and previous studies. With the advance of Oxford Nanopore long reads accuracy, hybrid assembly may not be necessary and long reads will be sufficient for generating high quality complete sequences with Canu or other tools.

Q2. The plasmids are constructed eventually by using hybrid assembly with Illumina data so the

idea of using MinION alone is not valid. Instead a post-polishing step can be done for Canu assembly to improve its quality. Have authors tried to do that? If Illumina data involved, in combination with real-time property of MinION (mentioned at line 86-87 and last paragraph of Dicussion), should it be a hybrid real-time assembly a more reasonable way to go?

Response: The reason for constructing full plasmid sequences by using hybrid assembly combining Illumina short read data and Oxford Nanopore long read data is the low accuracy of long read data. The accuracy of single long read is determined to be about 87% using a reference sequence. With Canu assembly along with the correction process based only on the long reads, the assembled plasmids can be 97% identical to the reference plasmids. Until now, Nanopore long read data cannot generate high quality assembled result without polishing with high quality short read data. This error is largely due to the inability of the present MinION platform to resolve the long homopolymers issue.

The hybrid real-time assembly is a great idea to obtain complete genome assemblies, and this idea has been validated with npScarf tool (Minh D. Cao et al. 2017). The hybrid real-time assembly of MDR-encoding should be feasible. However, hybrid real-time assembly is more appropriate in applications in clinical settings. Actually, we have tried to utilize jspsa tools for real-time analysis our data and found that the process required professional knowledge in bioinformatics and is too difficult to be used in conventional labs without dedicated bioinformatician. However, the workflow presented in our study is easy to be applied in most labs using laptops, without professional bioinformaticians. The time span of our workflow is ideal for studies on MDR-encoding plasmids (data generated within a few days), so a hybrid real-time assembly may not be necessary. However, we have also discussed the possible application of tools like npScarf for real-time assembly in the Discussion section.

Q3. Nanopore raw and Illumina data should be made public also; try to put everything (Illumina, Nanopore, plasmid assemblies...) into one location (e.g. a project in NCBI)

Response: We have deposited all the raw read data in the NCBI database with a project number PRJNA398365. All the supplemented data was deposited in the GigaDB repository.

4. Minor: table 1 samples ID (column 1) and table 2 plasmid size (column 2) shouldn't be broken.

Response: The formats of table 1 (column 1) and table 2 (column 2) have been revised to remove the broken lines.

Reviewer #2:

Q1: The authors present the use of oxford nanopore technology to sequence 12 plasmid extract samples and assemble these 21 complete plasmid sequences. They go on to compare the Canu derived assemblies with a hyrbrid Illumina assembly generated with UniCycler. The paper presents the methodologies used and the results clearly and this is a useful example of the use of this technology. The paper requires considerable edits in terms of use of the English language and clarity of phrase.

Response: The English language has been revised throughout the manuscript. All the phrases are also rechecked to keep clarity.

Abstract.
Q2: Line 34 - repetitive use of the name MinION.

Response: The sentence has been revised into "Here, we demonstrate a rapid and efficient approach to obtain multiple MDR plasmid sequences through the use of the MinION nanopore sequencing platform, which is incorporated in a portable device."

Q3: Line 41 - What is novel here?

Response: The workflow presented in this study is novel in several aspects: (i) the application of Oxford Nanopore rapid barcoding method in MDR-encoding plasmids is investigated for the first time; (ii) a flow cell can be re-used for plasmids samples which do not require a lot of sequencing data for assembly; (iii) Oxford Nanopore long read can cover all the plasmid sequence, hence de novo assembly may not necessary. However, due to the rapid development of sequencing technologies and related workflows, we delete the word "novel" here.

Introduction.
Q4: Line 51 - What do you mean by drivers in the is context. The main driver is overuse of antibiotics? Do you mean mechanisms?

Response: Drivers indicate the mechanisms responsible for widespread dissemination of AMR. To be concise, the sentence has been revised into "Identification of key mechanisms responsible for AMR transmission is crucial to combat the threats imposed by AMR."

Q3: Line 59 - I guess you mean Illumina and not Sanger in this context

Response: The conventional method to obtain complete plasmid sequences is to conduct de novo assembly using short read data (Illumina etc.) to generate contigs. PCR mapping based on the Sanger sequencing method will be used to close the gaps to finish the complete sequences. This sentence has been revised to enhance clarity.

Q4: Line 67 - Very vague sentence - what do you mean by advent of library preparation techniques and data analysis tools

Response: We tried to emphasize that the library preparation techniques and data analysis tools developed for Oxford Nanopore technologies in this study make it a very suitable technique for rapid and accurate MDR plasmid sequencing. This sentence has been revised.

Results.
Q5: Line 95 - Use of seriously is not good English in this context

Response: The word "seriously" was replaced by "severely".

Q6: Line 99 - What do you mean by high quality sequences here? % > Q20/30?

Response: High quality sequences mean the assembled plasmid sequences, not the raw reads. The hybrid assembly using Unicycler, when combined with short reads data, can generate high quality assembled plasmid sequences (regarded as reference sequences), compared with the Illumina de novo assembly results.

Q7: Line 100 - Use of About not needed

Response: The word “About” was deleted.

Discussion

Q8: Line 108 - How were the reference plasmids selected / created?

Response: The reference plasmids refer to complete plasmids assembled by Unicycler. As Illumina data is regarded as data with high accuracy, plasmid sequences assembled by Unicycler using Illumina data are regarded as reference plasmids in this study.

Q9: Line 110 - This sentence doesn't make sense

Response: “After obtaining the raw reads quality” was deleted. The sentence was revised into “Complete plasmids sequences obtained from de novo assembly by Canu, based on long reads, were compared to the reference plasmids (assembled by Unicycler) using BLASTN.”

We would like to thank the reviewers for taking time to assess our manuscript and share their insightful comments and suggestions. Based on the constructive reports, we have modified the manuscript and provide point-by-point responses to the comments:
