# Supplementary material for: Efficient generation of complete sequences of MDR-encoding plasmids by rapid assembly of MinION barcoding sequencing data
Source: Gigascience. 2018 Jan 9;7(3):gix132. doi: 10.1093/gigascience/gix132 (PMC5848804; doi:10.1093/gigascience/gix132)
Supplement: Response_to_Reviewer_Comments_Revision_1.pdf [file gix132_response_to_reviewer_comments_revision_1.pdf]

Author's Response To Reviewer Comments

Responses to reviewers
Reviewer reports:
Reviewer #1: The response from author is quite clear and adequate. The point of using only Nanopore data to complete genomes of interest in a productive way is made valid through 2 plasmids from sample RB01. However, authors should also include comparison of other Canu assemblies to make clear the pros and cons of using this approach.
Another piece of information that would be very helpful to include beside the main manuscript is the detail of using Canu software in this study. Canu relies on a required parameter, namely genomeSize, which is important and the choice of this parameter can vary the results significantly. Interestingly, the authors mentioned they set this parameter at 0.5m, 1m, 2m and 4m for optimization purpose. Regarding this, those who want to apply this method should want to know more about the optimization step that has been conducted, e.g. what exactly is the effect of changing parameter like that and how the final assembly being selected out of all possibilities (without hybrid assemblies or other data as reference).

Response: Based on the data of RB01, the plasmid sequence assembled by Canu using only Nanopore data showed 97% identity to the reference plasmids assembled by Unicycler using hybrid data of Nanopore and illumine reads. We further conducted similar analysis for other samples as shown in supplementary table 1 showing similar results as RB01. Our data indicated that the hybrid assembly performed by Unicycler was the accurate way to obtain complete genome sequences. For the sequences that could not be assembled by Unicycler, Canu was an option. To summarize, our data suggested that the pros of using Canu assemblies are high efficiency, potential for real-time sequencing and cost effective, while the disadvantage is lower accuracy when compared to hybrid assembly method. With the development of ONT technologies to improve the accuracy of single reads, it is suggested that Canu assemblies should be the best choice in the future. We have included this information in Line 120~128 of Results section.

Due to the variation of plasmid sizes and the contamination of chromosome in our samples, it is hard to select one fixed size for genomeSize for Canu analysis. We therefore used 0.5m, 1m, 2m and 4m as parameters for genomeSize for optimization purpose. In our study, numbers and sizes of the MDR plasmids in each sample were determined by S1-PFGE method prior to Nanopore sequencing, which greatly help the analysis and pick up right plasmids from the assembled data. Line 265~273.
