# Supplementary material for: Efficient generation of complete sequences of MDR-encoding plasmids by rapid assembly of MinION barcoding sequencing data
Source: Gigascience. 2018 Jan 9;7(3):gix132. doi: 10.1093/gigascience/gix132 (PMC5848804; doi:10.1093/gigascience/gix132)
Supplement: Reviewer_1_Report_(Original_Submission) -- Son Hoang Nguyen — 21 Jul 2017 Reviewed [file gix132_reviewer_1_report_(original_submission).pdf]

Reviewer Report

Title: Efficient generation of complete sequences of MDR-encoding plasmids by rapid assembly of MinION barcoding sequencing data

Version: Original Submission **Date:** 21 Jul 2017

Reviewer name: Son Hoang Nguyen

Reviewer Comments to Author:

1. This study is about a method to obtain completed MDR-encoding plasmids while minimizing the turnaround time. The idea is to use MinION barcode sequencing on extracted plasmids and then use computational tools to assembly them.

Although there are several studies conceived with similar approach (Sion C. Bayliss et al 2017, Ryan R. Wick et al 2017), they're focusing on completing the whole bacteria genomes rather than MDR-plasmids of interest alone.

The question is how this study serves its purpose more efficient than aforementioned methods, when the turn-around time and cost are about similar?

2. The plasmids are constructed eventually by using hybrid assembly with Illumina data so the idea of using MinION alone is not valid. Instead a post-polishing step can be done for Canu assembly to improve its quality. Have authors tried to do that?

If Illumina data involved, in combination with real-time property of MinION (mentioned at line 86-87 and last paragraph of Dicussion), should it be a hybrid real-time assembly a more reasonable way to go?

3. Nanopore raw and Illumina data should be made public also; try to put everything (Illumina, Nanopore, plasmid assemblies...) into one location (e.g. a project in NCBI)
4. Minor: table 1 samples ID (column 1) and table 2 plasmid size (column 2) shouldn't be broken.

Methods

Are the methods appropriate to the aims of the study, are they well described, and are necessary controls included? No

Conclusions

Are the conclusions adequately supported by the data shown? Yes

Reporting Standards

Does the manuscript adhere to the journal's guidelines on minimum standards of reporting? No

Statistics

Are you able to assess all statistics in the manuscript, including the appropriateness of statistical tests used? No, I do not feel adequately qualified to assess the statistics.

Quality of Written English

Please indicate the quality of language in the manuscript: Acceptable

Declaration of Competing Interests

Please complete a declaration of competing interests, considering the following questions:

- Have you in the past five years received reimbursements, fees, funding, or salary from an organisation that may in any way gain or lose financially from the publication of this manuscript, either now or in the future?
- Do you hold any stocks or shares in an organisation that may in any way gain or lose financially from the publication of this manuscript, either now or in the future?
- Do you hold or are you currently applying for any patents relating to the content of the manuscript?
- Have you received reimbursements, fees, funding, or salary from an organization that holds or has applied for patents relating to the content of the manuscript?
- Do you have any other financial competing interests?
- Do you have any non-financial competing interests in relation to this paper?

If you can answer no to all of the above, write 'I declare that I have no competing interests' below. If your reply is yes to any, please give details below.

I declare that I have no competing interests

I agree to the open peer review policy of the journal. I understand that my name will be included on my report to the authors and, if the manuscript is accepted for publication, my named report including any

attachments I upload will be posted on the website along with the authors' responses. I agree for my report to be made available under an Open Access Creative Commons CC-BY license (http://creativecommons.org/licenses/by/4.0/). I understand that any comments which I do not wish to be included in my named report can be included as confidential comments to the editors, which will not be published.

I agree to the open peer review policy of the journal

To further support our reviewers, we have joined with Publons, where you can gain additional credit to further highlight your hard work (see: https://publons.com/journal/530/gigascience). On publication of this paper, your review will be automatically added to Publons, you can then choose whether or not to claim your Publons credit. I understand this statement.

Yes
