# Supplementary material for: Efficient generation of complete sequences of MDR-encoding plasmids by rapid assembly of MinION barcoding sequencing data
Source: Gigascience. 2018 Jan 9;7(3):gix132. doi: 10.1093/gigascience/gix132 (PMC5848804; doi:10.1093/gigascience/gix132)
Supplement: Reviewer_1_Report_(Revision_1) -- Son Hoang Nguyen — 10 Oct 2017 Reviewed [file gix132_reviewer_1_report_(revision_1).pdf]

Reviewer Report

Title: Efficient generation of complete sequences of MDR-encoding plasmids by rapid assembly of MinION barcoding sequencing data

Version: Revision 1 **Date:** 10 Oct 2017

Reviewer name: Son Hoang Nguyen

Reviewer Comments to Author:

The response from author is quite clear and adequate. The point of using only Nanopore data to complete genomes of interest in a productive way is made valid through 2 plasmids from sample RB01. However, authors should also include comparison of other Canu assemblies to make clear the pros and cons of using this approach.
Another piece of information that would be very helpful to include beside the main manuscript is the detail of using Canu software in this study. Canu relies on a required parameter , namely genomeSize, which is important and the choice of this parameter can vary the results significantly. Interestingly, the authors mentioned they set this parameter at 0.5m, 1m, 2m and 4m for optimization purpose. Regarding this, those who want to apply this method should want to know more about the optimization step that has been conducted, e.g. what exactly is the effect of changing parameter like that and how the final assembly being selected out of all possibilities (without hybrid assemblies or other data as reference).

Methods

Are the methods appropriate to the aims of the study, are they well described, and are necessary controls included? Yes

Conclusions

Are the conclusions adequately supported by the data shown? Yes

Reporting Standards

Does the manuscript adhere to the journal's guidelines on minimum standards of reporting? Yes

Statistics

Are you able to assess all statistics in the manuscript, including the appropriateness of statistical tests used? Yes, and I have assessed the statistics in my report.

Quality of Written English

Please indicate the quality of language in the manuscript: Acceptable

Declaration of Competing Interests

Please complete a declaration of competing interests, considering the following questions:

- Have you in the past five years received reimbursements, fees, funding, or salary from an organisation that may in any way gain or lose financially from the publication of this manuscript, either now or in the future?
- Do you hold any stocks or shares in an organisation that may in any way gain or lose financially from the publication of this manuscript, either now or in the future?
- Do you hold or are you currently applying for any patents relating to the content of the manuscript?
- Have you received reimbursements, fees, funding, or salary from an organization that holds or has applied for patents relating to the content of the manuscript?
- Do you have any other financial competing interests?
- Do you have any non-financial competing interests in relation to this paper?

If you can answer no to all of the above, write 'I declare that I have no competing interests' below. If your reply is yes to any, please give details below.

I declare that I have no competing interests

I agree to the open peer review policy of the journal. I understand that my name will be included on my report to the authors and, if the manuscript is accepted for publication, my named report including any attachments I upload will be posted on the website along with the authors' responses. I agree for my report to be made available under an Open Access Creative Commons CC-BY license (http://creativecommons.org/licenses/by/4.0/). I understand that any comments which I do not wish to be included in my named report can be included as confidential comments to the editors, which will not be published.

I agree to the open peer review policy of the journal

To further support our reviewers, we have joined with Publons, where you can gain additional credit to further highlight your hard work (see: https://publons.com/journal/530/gigascience). On publication of this paper, your review will be automatically added to Publons, you can then choose whether or not to claim your Publons credit. I understand this statement.

Yes
