# Supplementary material for: Efficient generation of complete sequences of MDR-encoding plasmids by rapid assembly of MinION barcoding sequencing data
Source: Gigascience. 2018 Jan 9;7(3):gix132. doi: 10.1093/gigascience/gix132 (PMC5848804; doi:10.1093/gigascience/gix132)
Supplement: Reviewer_2_Report_(Original_Submission) -- Timothy Dallman — 08 Aug 2017 Reviewed [file gix132_reviewer_2_report_(original_submission).pdf]

Reviewer Report

Title: Efficient generation of complete sequences of MDR-encoding plasmids by rapid assembly of MinION barcoding sequencing data

Version: Original Submission **Date:** 08 Aug 2017

Reviewer name: Timothy Dallman

Reviewer Comments to Author:

The authors present the use of oxford nanopore technology to sequence 12 plasmid extract samples and assemble these 21 complete plasmid sequences.
They go on to compare the Canu derived assemblies with a hyrbrid Illumina assembly generated with UniCycler.
The paper presents the methodologies used and the results clearly and this is a useful example of the use of this technology.

The paper requires considerable edits in terms of use of the English language and clarity of phrase.

Abstract.
Line 34 - repetitive use of the name MinION.
Line 41 - What is novel here?

Introduction.
Line 51 - What do you mean by drivers in the is context. The main driver is overuse of antibiotics? Do you mean mechanisms?
Line 59 - I guess you mean Illumina and not Sanger in this context
Line 67 - Very vague sentence - what do you mean by advent of library preparation techniques and data analysis tools

Results.
Line 95 - Use of seriously is not good English in this context
Line 99 - What do you mean by high quality sequences here? % > Q20/30?
Line 100 - Use of About not needed

Discussion

Line 108 - How were the reference plasmids selected / created?
Line 110 - This sentence doesn't make sense

Methods

Are the methods appropriate to the aims of the study, are they well described, and are necessary controls included? Yes

Conclusions

Are the conclusions adequately supported by the data shown? Yes

Reporting Standards

Does the manuscript adhere to the journal's guidelines on minimum standards of reporting? Yes

Statistics

Are you able to assess all statistics in the manuscript, including the appropriateness of statistical tests used? There are no statistics in the manuscript.

Quality of Written English

Please indicate the quality of language in the manuscript: Needs some language corrections before being published

Declaration of Competing Interests

Please complete a declaration of competing interests, considering the following questions:

- Have you in the past five years received reimbursements, fees, funding, or salary from an organisation that may in any way gain or lose financially from the publication of this manuscript, either now or in the future?
- Do you hold any stocks or shares in an organisation that may in any way gain or lose financially from the publication of this manuscript, either now or in the future?
- Do you hold or are you currently applying for any patents relating to the content of the manuscript?
- Have you received reimbursements, fees, funding, or salary from an organization that holds or has applied for patents relating to the content of the manuscript?
- Do you have any other financial competing interests?
- Do you have any non-financial competing interests in relation to this paper?

If you can answer no to all of the above, write 'I declare that I have no competing interests' below. If your reply is yes to any, please give details below.

none

I agree to the open peer review policy of the journal. I understand that my name will be included on my report to the authors and, if the manuscript is accepted for publication, my named report including any attachments I upload will be posted on the website along with the authors' responses. I agree for my report to be made available under an Open Access Creative Commons CC-BY license (http://creativecommons.org/licenses/by/4.0/). I understand that any comments which I do not wish to be included in my named report can be included as confidential comments to the editors, which will not be published.

I agree to the open peer review policy of the journal

To further support our reviewers, we have joined with Publons, where you can gain additional credit to further highlight your hard work (see: https://publons.com/journal/530/gigascience). On publication of this paper, your review will be automatically added to Publons, you can then choose whether or not to claim your Publons credit. I understand this statement.

Yes
